# Targeting Drp1 inhibits ESCC progression via the ROS-PGC1-α-Nrf1/2 pathway

**DOI:** 10.1186/s12967-025-06697-8

**Published:** 2025-06-17

**Authors:** Zhixiong Jiang, Yating Yang, Jinchi Zhou, Xin Li, Qingqing Meng, Xiangyi Yu, Yaxin Xue, Mengyu Li, Yichen Cai, Pengchun Han, Mingjun Jiang, Huizhen Wang, Congrong Liu, Jing Zhao, Lixin Wan, Dengke Bao

**Affiliations:** 1 Laboratory of Cancer Biomarkers and Liquid Biopsy, School of Pharmacy, Henan University, Kaifeng, 475000 Henan China; 2 The Zhongzhou Laboratory for Integrative Biology, Henan University, Zhengzhou, 450000 Henan China; 3 First Affiliated Hospital of Henan University, Kaifeng, 475000 Henan China; 4 Department of Gastroenterology, 962 Hospital, Joint Logistic Support Force of PLA, Harbin City, 150080 China; 5 Zhumadian Preschool Education College, Zhumadian, 463000 Henan China; 6 Henan Engineering Research Center for Molecular Diagnosis and Therapy of Esophageal Cancer Nanyang Central Hospital, Henan University, Nanyang, 473000 Henan China

**Keywords:** Esophageal squamous cell carcinoma, Drp1, ROS, Metastasis, MiR-203a-3p

## Abstract

**Background:**

Esophageal squamous cell carcinoma (ESCC) ranks among the most prevalent malignancies of the digestive tract. Due to the absence of obvious symptoms in patients with early-stage ESCC, most cases are diagnosed at advanced stages, highlighting the urgent need to investigate the specific mechanisms underlying ESCC progression. Mitochondrial dysfunction plays a pivotal role in tumor progression by regulating multiple biological processes. Dynamin-related protein 1 (Drp1), which is involved in the regulation of mitochondrial fission, is closely associated with tumor progression. However, its role in the metastasis of ESCC remains to be fully elucidated.

**Methods:**

This study utilized database analysis and immunohistochemistry to evaluate the expression of Drp1 in ESCC tissues. Functional cell experiments and mouse models were performed to elucidate the mechanisms by which Drp1 influences ESCC cell growth and metastasis. Furthermore, the TargetScan online platform was employed to predict microRNAs that may interact with Drp1 to further explore the specific mechanism of Drp1 on the progression of ESCC.

**Results:**

We found that high expression of Drp1 was correlated with poor prognosis of ESCC patients. Furthermore, Drp1 overexpression significantly enhanced the growth and metastasis of ESCC cell both in vitro and in vivo. Mechanistically, we showed that Drp1 overexpression activated the PGC1-α-Nrf1/2 signaling and promoted the process of epithelial–mesenchymal transition (EMT) in ESCC cells, thereby facilitating tumor cell metastasis. Additionally, miR-203a-3p targeted and down-regulated Drp1 expression in ESCC cells, effectively inhibiting Drp1-mediated metastasis through the ROS-PGC1-α-Nrf1/2 pathway.

**Conclusions:**

These findings uncover that Drp1 overexpression drived the growth and metastasis of ESCC via ROS-PGC1-α-Nrf1/2 signaling pathway, while miR-203a-3p significantly inhibited Drp1 expression and its capacity to mediate the malignant progression of ESCC cells. Our results provide potential novel therapeutic targets for the treatment of ESCC.

**Supplementary Information:**

The online version contains supplementary material available at 10.1186/s12967-025-06697-8.

## Introduction

Esophageal cancer ranks among the most prevalent malignancies of the digestive tract, characterized by substantial morbidity and mortality. Esophageal squamous cell carcinoma (ESCC) constitutes 90% of all esophageal cancers and is particularly predominant in developing nations [1]. Due to the absence of distinct early symptoms, patients often receive a diagnosis at advanced stages, frequently accompanied by extensive metastasis [2]. Therefore, elucidating the molecular mechanisms underlying ESCC metastasis is of significant importance.

Mitochondrial dysfunction is intricately linked to tumorigenesis and cancer progression. Mitochondria serve as crucial sites for the generation of intracellular reactive oxygen species (ROS), which influence cancer pathogenesis [1]. Several studies have demonstrated that ROS can activate peroxisome proliferator-activated receptor gamma coactivator-1 alpha (PGC1-α), nuclear factor E2 related factor 1 (Nrf1) or nuclear factor E2 related factor 2 (Nrf2). These activations affecting mitochondrial biosynthesis and subsequently impact the malignant progression of various cancers, including glioblastoma, non-small cell lung cancer and hepatocellular carcinoma [3–5].

Dynamin-related protein 1 (Drp1), a member of the guanosine triphosphatase (GTPase) superfamily [6], features domains including the N-terminal GTPase domain, dynein-like intermediate domain, and C-terminal GTPase effector domain [7]. Relevant studies have pointed out that Drp1 deficiency or mutation-mediated changes in mitochondrial morphology result in mitochondrial dysfunction, which can affect the malignant progression of endometrial cancer, lung cancer and colorectal cancer by impacting the cell cycle, epithelial-mesenchymal transition and other processes [8–11]. Nonetheless, the mechanisms that link Drp1 and the metastasis of ESCC remain incompletely understood.

MicroRNAs (miRNAs) are single-stranded RNAs that arise from the transcription and processing of endogenous long precursor RNAs. MiRNAs regulate gene expression by binding to the 3'-UTRs of mRNAs, thereby significantly influencing normal biological processes, as well as tumour invasion and migration [12]. Several studies have demonstrated that the expression levels of tumor suppressor miRNAs in ESCC tissues are significantly reduced. These miRNAs play a crucial role in influencing the metastatic potential of ESCC [13]. For instance, miRNAs such as miR-138, miR-193a-5p, miR-206, miR-451, and miR-718 are notably decreased in ESCC tissues, and correlates with lymph node metastasis (LNM) and the TNM stage of ESCC [14–18]. Among these, miR-101 has been shown to inhibit the migration and invasion of ESCC by activating its target genes, COX-2, MALAT1, or EZH2 [18–20]. Additionally, the downregulation of the miR-574-3p target genes FAM3C/MAPK1, COX-2, MALAT1, or EZH2 can significantly inhibit the migration and invasion of ESCC cells both in vitro and in vivo [21]. Other studies have indicated that miR-203a-3p is associated with poor prognosis in soft tissue sarcoma [22] and colorectal cancer [23]. Furthermore, it can influence the growth and metastasis of non-small cell lung cancer [24], pancreatic cancer [25], hepatocellular carcinoma [26], and ovarian cancer [27] by activating pathways such as PI3K/Akt and Akt/GSK-3β/Snail. We demonstrated that miR-203a-3p can target and regulate the expression of Drp1 in ESCC tissues, thereby inhibiting the growth and metastasis of ESCC cells, although the specific mechanism remains to be elucidated.

Our findings indicated that Drp1 promotes the metastasis of ESCC cells by activating the ROS-PGC1-α-Nrf1/2 pathway, with its expression regulated by miR-203a-3p. These insights into the underlying mechanisms provide a novel therapeutic target for managing ESCC metastasis.

## Materials and methods

### Tissue samples

ESCC tissue samples, comprising 102 pairs of primary ESCC tissues and adjacent normal tissues samples from ESCC patients who underwent surgery treatment were obtained from Nanyang central Hospital of Henan University in Kaifeng, China. There were no biases in selecting patients, and none of the patients had received any prior treatment, such as radiotherapy or chemotherapy. These samples were promptly preserved at − 20 °C using RNA later reagent and subsequently embedded in paraffin. The demographic variables, and clinical and follow-up data of each patient were summarized in Supplementary Table S1 in Supplementary Material 1. The latest follow-up date was July 2017. Ethical approval for all procedures was obtained from the Henan University Protection of Human Subjects Committee (Approval Number: HUSOM2022-234). Prior to their inclusion in this study, written informed consent was provided by all participating patients.

### Cell lines

The human ESCC cell lines (KYSE-30, KYSE-70, and EC9706) were obtained from Cobioer (Nanjing, China). These cell lines were cultured in Dulbecco’s Modified Eagle Medium (DMEM, CORNING) supplemented with 10% fetal bovine serum (BI) in a humidified 5% CO_2_, 37 °C. For ectopic expression, gene knockdown using shRNA, and miR-203a-3p mimic transfection, overexpression Drp1 oligos were synthesized and cloned into the V3 vector. Lentivirus production involved co-transfecting HEK293T cells with the respective plasmids alongside psPAX2 and pMD2.G. After 48 h, lentivirus was harvested through filtration (0.45 μm pore size). The collected lentivirus was used immediately for cell infection with polybrene or stored at −80 °C. Infected cells were selected using puromycin (APE × BIO) at a concentration of 3 μg/mL. The miR-203a-3p mimic was custom-designed and synthesized by Ribobio (Guangzhou, China). Lentivirus and the miR-203a-3p mimic were transfected into cells following established protocols. Detailed information regarding lentivirus, the miR-203a-3p mimic, shRNAs, siRNAs and qRT-PCR primer sequences can be found in Supplementary Table S2 and table S3 in Supplementary Material 1.

### Colony-forming assay

Cells in the logarithmic growth phase were dissociated into a single-cell suspension and subsequently centrifuged at 1000 rpm for 5 min. Following resuspension in 1 mL of DMEM, the cells were enumerated, and 2000 cells were seeded per well in a 6-well plate. After incubation, the cells were fixed using 4% paraformaldehyde and then stained with crystal violet. Clone formation efficiency was assessed using ImageJ software for quantification.

### EdU cell proliferation

The EdU Kit (Ribobio, Guangzhou, China) was utilized for assessing cell proliferation. Briefly, cells were seeded at a density of 1 × 10^3^ cells per well in 96-well plates and allowed to adhere for 24 h. Subsequently, EdU reagent (100 μL) was added to each well and incubated for 2 h. Cells were then fixed with 4% paraformaldehyde for 40 min, followed by glycine treatment for 5 min to neutralize paraformaldehyde. After permeabilization with Triton-X 100, cells were stained with Apollo. Finally, Hoechst 33,342 was used for nuclear staining. Cell counting and analysis were performed using fluorescence microscopy.

### Cell cycle assay

1 × 10^6^ cells were centrifuged and then fixed with precooled absolute ethanol and PBS overnight. After washed with PBS, cells were centrifuged to remove the supernatant and added 0.5 mL propidium iodide staining solution, for 30 min at room temperature in darkness. Cell cycle analysis was performed by Flow J software was applied for flow cytometry analysis.

### Wound healing assay

For wound healing assay, 2 × 10^5^ cells were seeded in 6-well. After 24 h, a wound was made in each well with a 10 μL pipette tip. Wells were imaged as healed and after 24 h interval. Wound width was measured and analyzed by Image J software.

### Transwell assays

Invasion and migration assays of the cells were conducted using Transwell chambers (Corning, USA), which were either coated or uncoated with Matrigel (Corning, USA). Cells resuspended at a concentration of 2 × 10^5^ cells/mL in 200 μL of serum-free medium supplemented with 1% BSA were seeded onto the apical side of the chamber. The basolateral side of the chamber was filled with 500 μL of medium containing 10% fetal bovine serum (FBS). After a 48-h incubation period, cells that had invaded or migrated through the membrane were fixed with 4% paraformaldehyde and stained with 0.1% crystal violet solution for 30 min. The invaded or migrated cells were subsequently quantified using a phase-contrast microscope (Olympus, Japan).

### Quantitative real-time PCR (qRT-PCR)

Total RNA was extracted from cells using TRIzol LS Reagent (Thermo Fisher Company, USA) following the manufacturer's instructions. The extracted RNAs were reverse-transcribed using a reverse transcription kit (Takara, Japan). Quantitative real-time PCR was conducted using SYBR Green Master Mix (Accurate Biology, China) on a real-time quantitative PCR system (BioRad, USA). The relative expression levels of various gene sets were normalized to GAPDH mRNA. Primer sequences used in this study were synthesized by Tsingke Biotechnology Co., Ltd. (China) and are listed in Supplementary Table S3 in Supplementary Material 1.

### Western blotting

All cells were lysed using radioimmunoprecipitation assay (RIPA) lysis buffer (Beyotime, China) supplemented with 1% phosphatase inhibitor cocktail II and 1% protease inhibitor cocktail (Roche, China), followed by centrifugation at 4 °C for 15 min. Protein concentrations were determined using a BCA protein assay kit (Solarbio, China). Subsequently, proteins were separated by 10% sodium dodecyl sulfate polyacrylamide gel electrophoresis (SDS-PAGE), transferred to PVDF membranes (MILLIPORE, USA), and blocked with 5% defatted milk at room temperature for 1 h. Membranes were then incubated overnight at 4 °C with diluted primary antibodies. Immunodetection was performed using the ECL Plus chemiluminescence detection system (Solarbio, China), and band intensities were analyzed using Image Lab software (Bio-Rad Laboratories, USA). The primary antibodies used in this study and their working concentrations are listed in Supplementary Table S4 in Supplementary Material 1.

### Mitochondrial morphology by transmission electron microscopy and confocal microscopy

ESCC cells were fixed with 4% glutaraldehyde and post fixed with 1% OsO_4_ in 0.1 M cacodylate buffer containing 0.1% CaCl_2_ for 2 h at 4 °C. After staining with 1% uranyl acetate, samples were dehydrated through an ethanol gradient and embedded in araldite resin. Thin sections were stained with uranyl acetate and lead citrate and analyzed with an electron microscope (Tecnai G2, FEI), at 11,500 × magnification.

TOM20-antibody was used to monitor mitochondrial morphology according to the manufacturer’s instructions. Briefly, 2 × 10^4^ ESCC cells were cultured for 48 h, fixed with 4% paraformaldehyde, permeabilized with 0.1% Triton X-100, and stained with TOM-20 antibody for 1.5 h at 37 °C. After washing with PBS for 3 times, ESCC cells were imaged using a Nikon N-SIM Structured Illumination microscope (Nikon, Nikon/A1 + N-SIM). For morphometric analysis, Image J software was used to measure the length of mitochondria. The primary antibodies used in this study and their working concentrations are listed in Supplementary Table S4 in Supplementary Material 1.

### Detection of reactive oxygen species in cells

Cellular reactive oxygen species (ROS) levels were detected by the fluorescent probe DCFH-DA (S0033S, Beyotime Biotechnology) following the manufacturer’s protocols. Briefly, ESCC cells were seeded in 6-well plates at a density of 2 × 10^5^ cells/mL and cultured for 48 h. The cells were digested using EDTA-free trypsin, centrifuged, and resuspended in 10 μM DCFH-DA diluted with serum-free medium. Following this, the cells were incubated for 20 min in an incubator. Then cells were centrifuged and resuspended by washing with PBS. Fluorescence intensity was measured by flow cytometry.

### Detection of mitochondrial membrane potential

JC-1 dye (C2006, Beyotime Biotechnology) was employed to assess detect the mitochondrial membrane potential. Cells were adjusted to a density of 2 × 10^5^/ml and stained with 5 mg/L JC-1 dye for 20 min at 37 ℃. After washing with the dye buffer three time, the cells were centrifuged and resuspended in PBS. The fluorescence intensity was measured by flow cytometry.

### Detection of ATP measurement

Plate 2 × 10^5^/ml cells in a 6-well plate and incubate overnight. Measure the ATP level in the cells according to the instruction manual of the ATP Assay Kit (Beyotime, S0026). The relative luminescence units (RLU) were determined using the luminometer according to the manufacturer's instructions. The data were normalized to the number of cells.

### Immunohistochemistry

The slides were placed in a 60 °C heating incubator for 1 h to facilitate uniform temperature exposure. Subsequently, they underwent deparaffinization, hydration, and antigen retrieval procedures, followed by inactivation of endogenous peroxidase activity. The slides were then incubated with primary antibodies against Drp1. Afterward, they were treated with horseradish peroxidase (HRP)-conjugated secondary antibodies derived from goat anti-rabbit. DAB reagent was employed for chromogenic staining of the slides.

The IHC staining was quantitatively analyzed using ImageJ software. Initially, the IHC-stained images were imported into ImageJ. The color deconvolution plugin was employed to separate the DAB staining from the hematoxylin counterstain. Thresholds were then set to differentiate the positive staining areas from the background. The positive staining areas were measured using the measurement function in ImageJ and expressed as a percentage of the total tissue area. The analysis was performed on at least three representative areas from each sample to ensure accuracy and reliability.

### In vivo tumor formation and metastasis assays

Six-week-old male BALB/c-nude mice were procured from Beijing Vital River Laboratory Animal Technology Co., Ltd., and maintained under specific pathogen-free (SPF) conditions in the Experimental Animal Center of Henan University. All experimental procedures involving animals were approved by the Henan University Animal Care Committee (approval number: HUSOM2022-234). For in vivo metastasis assays, a suspension of 1 × 10^6^ cells in 200 μL of PBS was injected into the tail veins of nude mice (n = 10 per group). Two weeks following the tail vein injection of EC9706 cells, intraperitoneal injections of DMSO (10%) and Mdivi-1 were administered to observe the inhibitory effects of Mdivi-1 on Drp1-mediated ESCC metastasis. Six weeks post-injection, the mice were euthanized, and their livers and lungs were dissected to examine metastatic lesions using histological analysis and hematoxylin–eosin staining.

## Results

### Upregulated Drp1 expression is associated with malignant progression and poor prognosis of ESCC patients

**Fig. 1:**
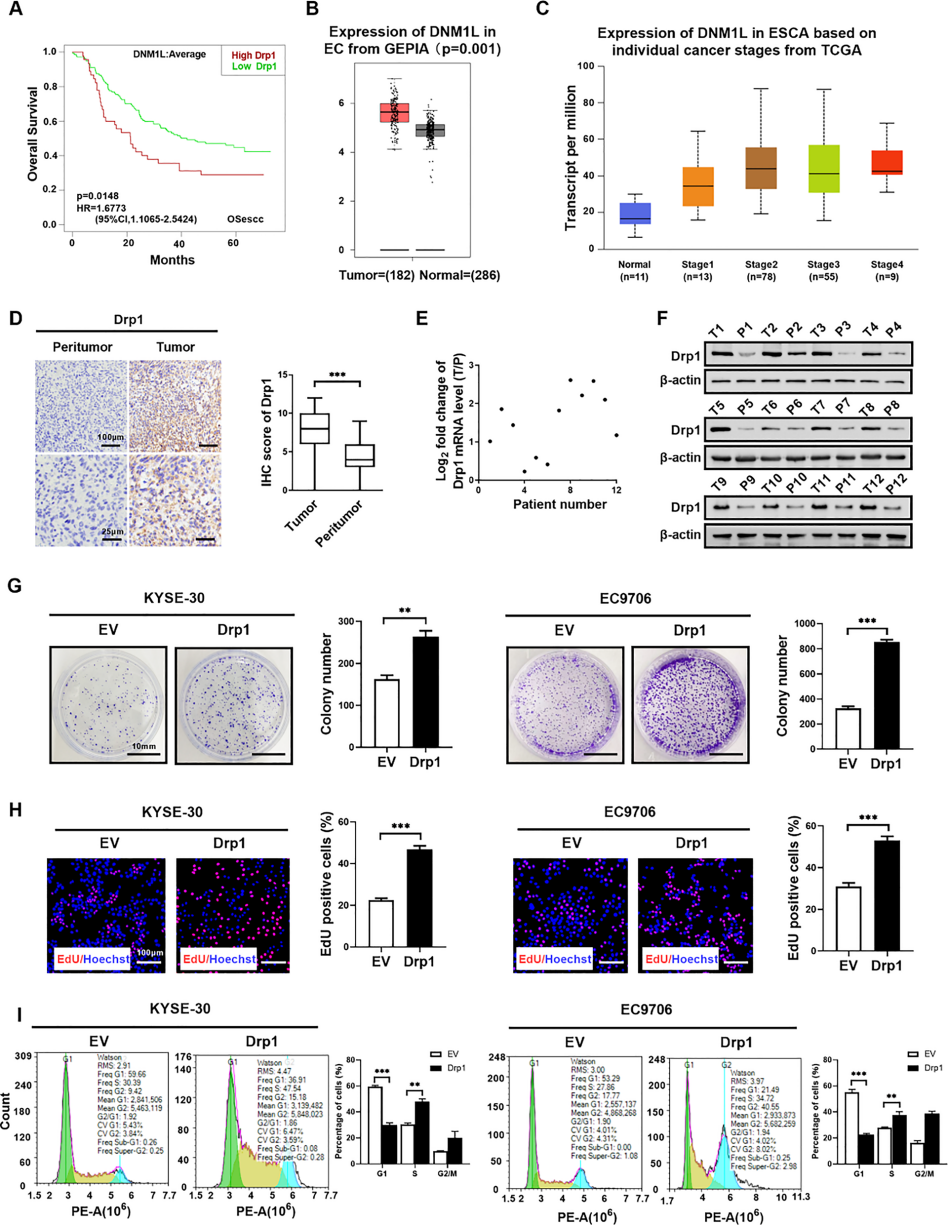
Upregulated Drp1 expression is associated with malignant progression and poor prognosis of ESCC patients. **A** LOGpc (Biomedical Informatics Institute) database analysed the Kaplan–Meier curve of overall survival in ESCC patients by the expression of Drp1 in tumour tissues. **B** GEPIA assessed the level of Drp1 in tissues derived from patients with ESCC. **C** TCGA database analysed the expression of Drp1 in ESCC based on individual cancer stages. **D** Representative immunohistochemistry (IHC) images depict the staining intensity of Drp1 (left) and quantified Drp1 levels (right) in tumour tissues of patients with ESCC. (n = 102). Scale bars: 25 μm. **E** and **F** QRT-PCR assay (**E**) and Western blotting assay (**F**) for the expression level of Drp1 in paired tissues from ESCC patients. The relative expression ratio of tumor to peritumor tissues was log2-transformed for subsequent statistical analysis (n = 12). **G** and **H** Colony-forming assay (**G**) and EdU incorporation assay (**H**) for the proliferation of ESCC cells with Drp1 overexpression and control cells (n = 3 independent experiments). Scale bars: 100 μm. EV, empty vector; Drp1, expression vector encoding Drp1. **I** Representative flow cytometry images of cell cycle in ESCC cells with treatment as indicated (n = 3 independent experiments). Data presentation: Graphs represent mean ± SEM. Statistical analyses included two-tailed unpaired t-tests for (**D**, **E**, **H**) and one-way ANOVA for (**E** and **I**). p-values from t-tests are denoted as follows: *p < 0.05; **p < 0.01; ***p < 0.001

To investigate the clinical relevance of Drp1 in ESCC, we analyzed the effect of Drp1 on survival rates among ESCC patients and compared its expression between cancerous tissues and adjacent non-cancerous tissues using data from the Cancer Genome Atlas (TCGA) (https://ualcan.path.uab.edu/cgi-bin/TCGAExResultNew2.pl?genenam=DNM1L&ctype=ESCA) and LOGpc (Biomedical Informatics Institute) databases. Our results revealed that patients exhibiting higher Drp1 expression showed significantly shorter overall survival rates (Fig. 1A). Moreover, Drp1 expression was significantly upregulated in ESCC tissues compared to adjacent normal tissues (p = 0.001) (Fig. 1B), with no significant differences observed among different tumor stages (Fig. 1C). Immunohistochemical (IHC) assays further confirmed the increased expression of Drp1 in ESCC tissues compared to adjacent non-cancerous tissues (n = 102) (Fig. 1D). Furthermore, both mRNA and protein levels of Drp1 were notably increased in ESCC tissues relative to paired non-cancerous tissues (Fig. 1E, F and Fig. S1A).

The above findings prompts us to investigate the molecular functions of Drp1 in the progression of ESCC. Consequently, we established ESCC cell models with Drp1 overexpression and knockdown to elucidate its functional role in ESCC progression (Fig. S1B–S1G). Specifically, Drp1 overexpression in ESCC cells led to a significant increase in colony formation (Fig. 1G), a higher proportion of EdU-positive cells (Fig. 1H), and an accelerated cell cycle (Fig. 1I). Collectively, these findings suggested that elevated Drp1 expression was associated with poorer overall survival in ESCC patients and highlighted its role in promoting ESCC cell proliferation.

### Overexpression of Drp1 promotes the metastasis of ESCC cells both in vitro and in vivo

**Fig. 2:**
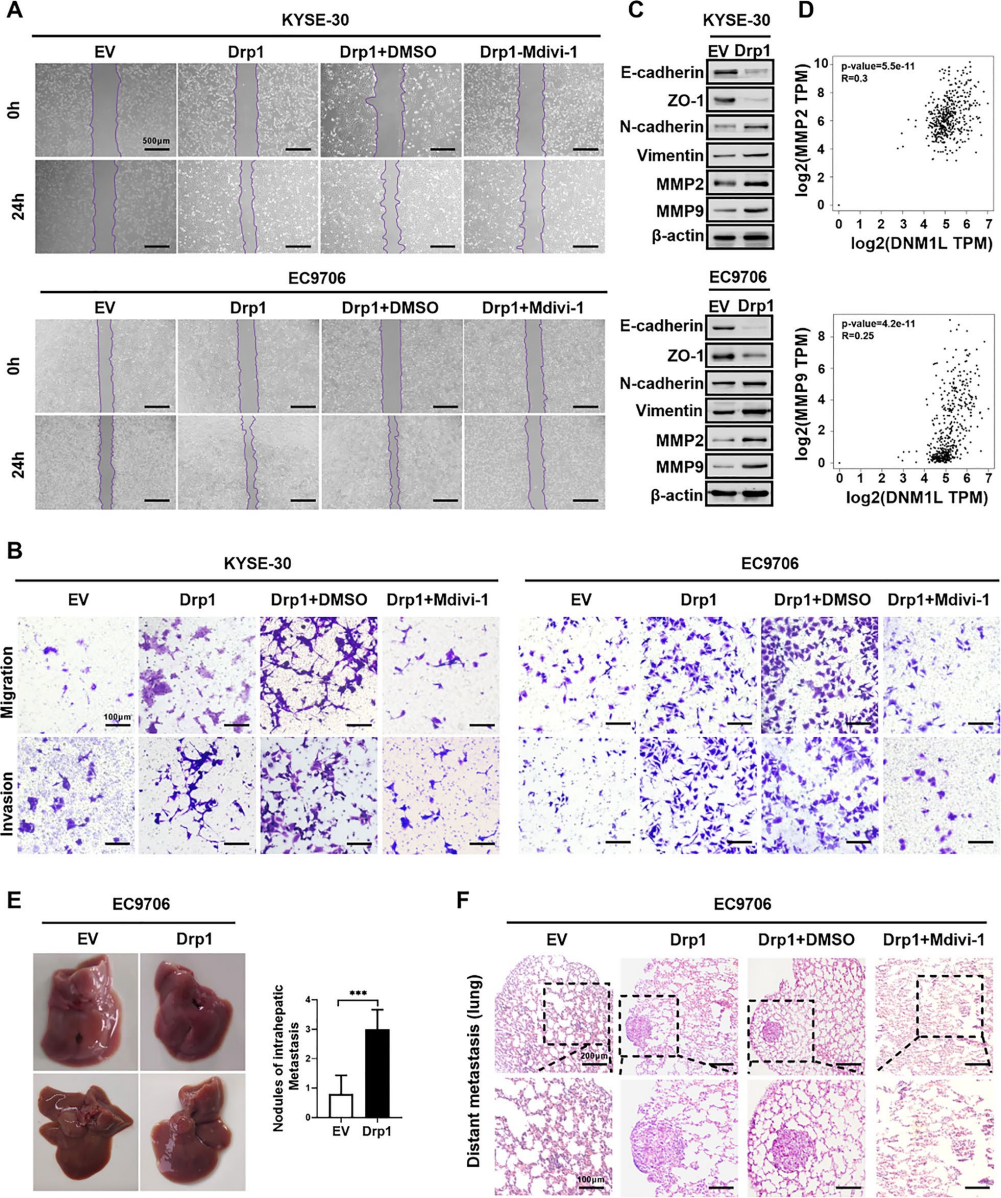
Overexpression of Drp1 promotes the metastasis of ESCC cells both in vitro and in vivo. **A** and **B** Wound-healing migration assay (**A**) and Transwell migration and invasion assays (**B**) for ESCC cells with Drp1 overexpression or treated with Mdivi-1 (n = 3 independent experiments). Mdivi-1 refers to treatment with 50 μM Mdivi-1 for 12 h. Scale bars: 100 μm. **C** Western blotting assay for protein expression level of MMP2, MMP9, and EMT-related gene in ESCC cells with Drp1 overexpression. **D** Pearson correlation analysis between the levels of Drp1 and MMP2, MMP9 in ESCC tissues from the GEPIA database (The relative expression ratio of tumour to non‐tumour was log2‐transformed. MMP2: Pearson’s correlation coefficient R = 0.3, *p* < 0.01; MMP9: Pearson’s correlation coefficient R = 0.25, *p* < 0.01). **E** Gross images illustrating liver metastasis (left) and quantification of metastatic nodules per intrahepatic area (right) in mice (n = 10 per group). **F** Representative H&E images displaying lung metastasis in mice treatment as indicated. (Mdivi-1, 42.5 μM) (n = 10 per group). Scale bars: 100 μm

Both in vitro and in vivo gain-of-function studies were conducted to evaluate the impact of Drp1 on the metastatic potential of ESCC cells. As shown in Fig. 2A, B and Fig. S2A, 2B, Drp1 overexpression significantly augmented the migration and invasion capabilities of KYSE-30 and EC9706 cells. Conversely, treatment with Mdivi-1, a Drp1 inhibitor, mitigated the enhanced migration and invasion abilities of ESCC cells (Fig. 2A, B and Fig. S2A, 2B). Multiple studies have demonstrated that processes such as matrix metalloproteinase activity and epithelial mesenchymal transition (EMT) play important roles in ESCC progression [28–30]. Subsequently, western blotting analysis revealed that Drp1 overexpression notably promoted the process of EMT as well as the levels of MMP2 and MMP9 in ESCC cells (Fig. 2C and Fig. S2C, 2D). Moreover, analysis of data from TCGA database demonstrated a significant positive correlation between Drp1 and both MMP2 and MMP9 (MMP2, Pearson’s correlation R = 0.3, *p* < 0.01; MMP9, Pearson’s correlation R = 0.25, *p* < 0.01) (Fig. 2D). Immunohistochemistry results further indicated an upregulation of Drp1 and MMP2, showing a positive correlation between Drp1 protein expression and MMP2 protein expression in ESCC tissues (Fig. S2E).

To further investigate the metastatic capabilities of Drp1 in vivo, EC9706 cells with Drp1 overexpression were injected into the tail vein of nude mice to establish xenograft tumours. Moreover, lung metastasis was assessed through hematoxylin and eosin (H&E) staining. The results depicted in Fig. 2E demonstrate that the group with Drp1 overexpression exhibited liver metastatic nodules compared to the control group. Conversely, nude mice xenograft tumours of ESCC cells treated with Mdivi-1 showed a lower number of lung metastatic lesions (Fig. 2F). Overall, these findings indicated that Drp1 overexpression significantly enhanced the metastasis of ESCC cells both in vitro and in vivo.

### Targeting Drp1 inhibits the progression of ESCC cells

**Fig. 3:**
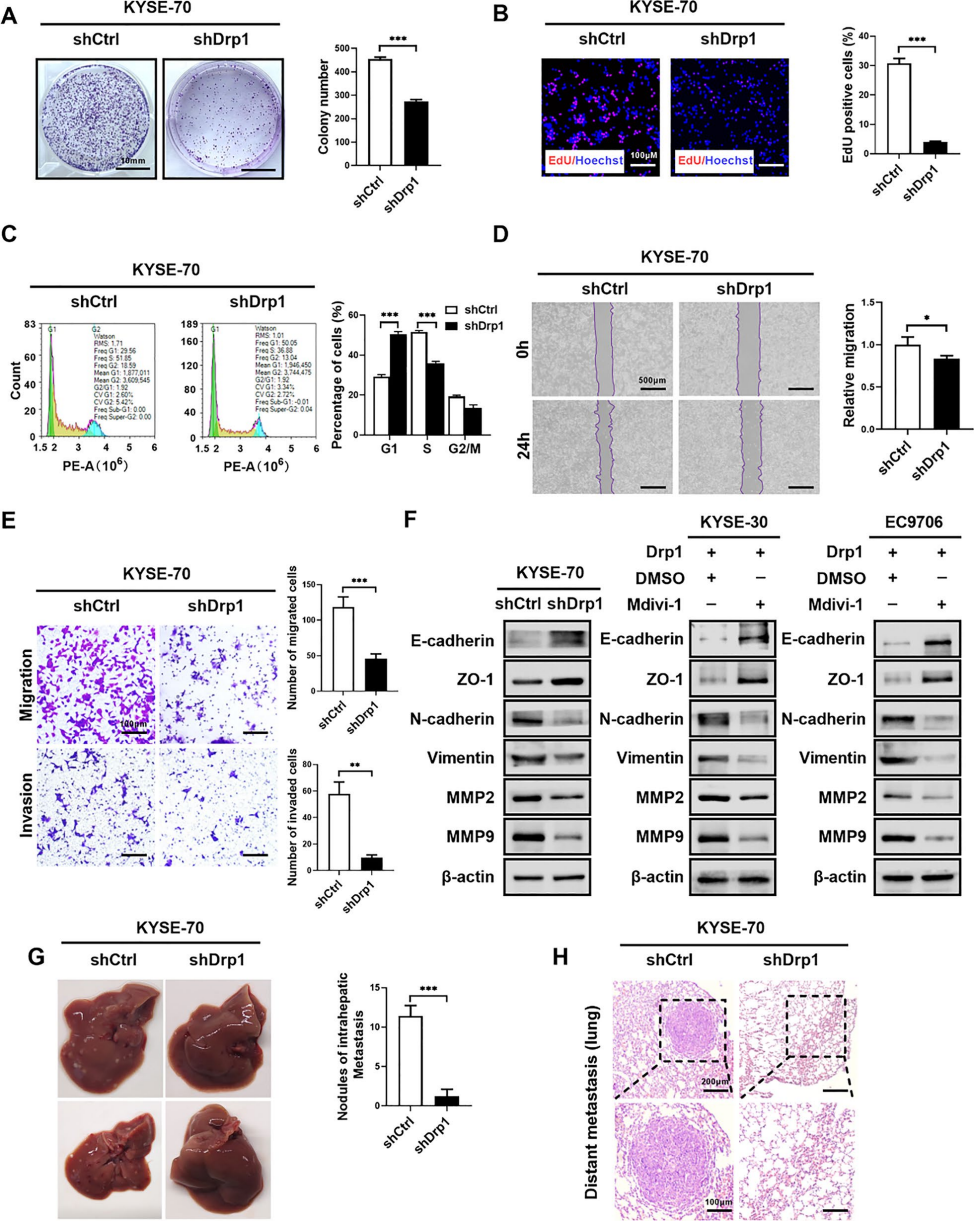
Targeting Drp1 inhibits the progression of ESCC cells. **A** and **B** Colony-forming assay (**A**) and EdU incorporation assay (**B**) for the proliferation of ESCC cells with Drp1 knockdown (n = 3 independent experiments). Scale bars: 100 μm. shCtrl denotes control shRNA, while shDrp1 indicates shRNA targeting Drp1. **C** Representative flow cytometry images of cell cycle in KYSE-70 cells with treatment as indicated (n = 3 independent experiments). **D** and **E** Wound-healing migration assay (**D**) and Transwell migration and invasion assays (**E**) for KYSE-70 cells with Drp1 overexpression or treated with Mdivi-1 (n = 3 independent experiments). Scale bars: 100 μm. **F** Western blotting assay for protein expression level of MMP2, MMP9, and EMT-related gene in ESCC cells with treatment as indicated. **G** Gross images of livers (left) and nodules of intrahepatic metastasis (right) following tail vein injection of ESCC cells with Drp1 knockdown (n = 10 per group). **H** Representative H&E images of lung metastasis in mice from (**G**) (n = 10 per group). Scale bars: 100 μm. Data information: Graphs represent mean ± SEM. Statistical analysis involved two-tailed unpaired t-tests for (**A**, **B**, **D**, **E**, **G**) and one-way ANOVA for (**C**). p-values from t-tests are denoted as *p < 0.05; **p < 0.01; ***p < 0.001

To further substantiate the tumorigenic role of Drp1, KYSE-70 cells with Drp1 downregulation were utilized to investigate its impact on the proliferation and metastasis of ESCC cells. Our results demonstrated that the knockdown of Drp1 in ESCC cells led to a significant decrease in colony formation (Fig. 3A), a reduced proportion of EdU-positive cells (Fig. 3B), and an arrested cell cycle (Fig. 3C). Additionally, downregulation of Drp1 significantly diminished the migration and invasion capabilities of ESCC cells (Fig. 3D¸ E). Furthermore, western blotting results indicated that the downregulation of Drp1 inhibited the EMT process in ESCC cells, consistent with the results observed in the Mdivi-1 treatment group (Fig. 3F and Fig. S3). Metastasis assays conducted in nude mice confirmed a lower number of metastatic nodules in the liver and lungs of the Drp1-knockdown group compared to the control group (Fig. 3G, H). Collectively, these findings suggested that the downregulation of Drp1 attenuated the proliferative and metastatic capabilities of ESCC cells.

### Drp1 accelerates ESCC cell metastasis through the ROS-PGC1-α-Nrf1/2 pathway

**Fig. 4:**
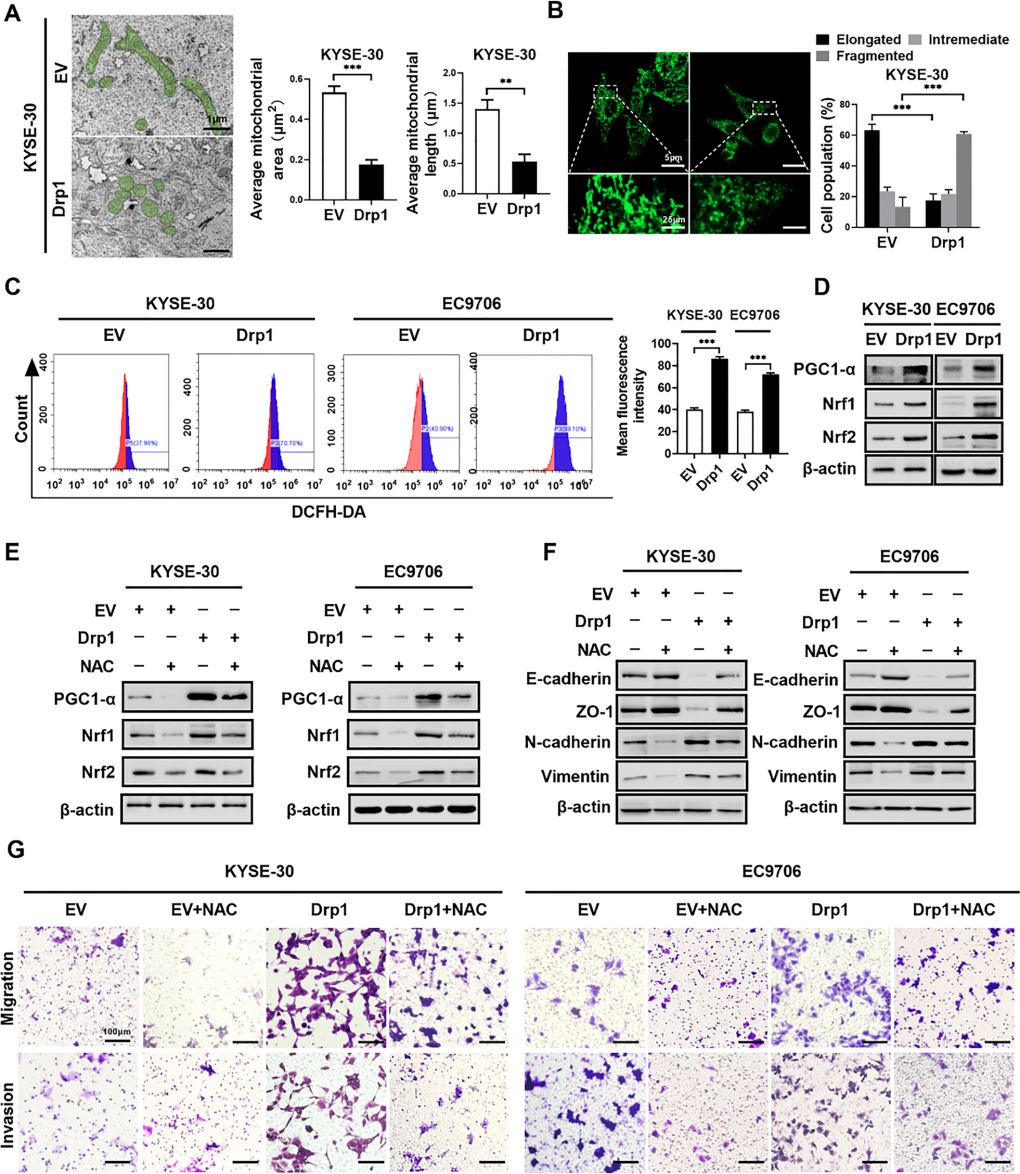
Drp1 accelerates ESCC cell metastasis through the ROS-PGC1-α-Nrf1/2 pathway. **A** Representative mitochondrial morphology images of transmission electron microscopy in KYSE-30 cell with Drp1 overexpression (n = 3 independent experiments). Scale bars: 1 μm. **B** Representative mitochondrial morphology images of confocal microscopy in KYSE-30 cell treatment as indicated (n = 3 independent experiments). Scale bars: 5 μm. **C** Representative flow cytometry images of ROS in KYSE-30 and EC9706 cells with Drp1 overexpression (n = 3 independent experiments). **D** and **E** Western blotting assay for the expression levels of PGC-1α, Nrf1, and Nrf2 in ESCC cells with stable overexpression of Drp1 (**D**) or treatment with 20 μM N-Acetyl-L-cysteine (NAC) for 12 h (**E**). **F** Western blotting assay for the expression levels of EMT-related gene in ESCC cells with treatment as indicated (n = 3 independent experiments). **G** Transwell migration and invasion assays for ESCC cells treatment as indicated (n = 3 independent experiments). Scale bars: 100 μm

Mitochondrial dysfunction, which is characterized by an imbalance between fission and fusion, metabolic disruption, and overproduction of reactive oxygen species (ROS), drives the aggressiveness of ESCC by fueling proliferation, invasion, and resistance to therapy [31–33]. Reactive oxygen species (ROS) play a pivotal in the pathogenesis of cancer cells. We investigated the influence of Drp1 on ROS production in ESCC cells. Our findings demonstrated mitochondrial fragmentation and abnormally elevated ROS levels in the Drp1 overexpression group compared to the control in ESCC cells (Fig. 4A–C). Previous research has identified the PGC1-α-Nrf1/2 pathway as critical in ROS regulation [34, 35]. Subsequently, we detected the expression of PGC1-α-Nrf1/2 signalling pathway-related proteins in ESCC cells. Our results showed that the levels of PGC1-α, Nrf1, and Nrf2 were increased when Drp1 was overexpressed, and these levels were significantly reduced after treatment with the potent antioxidant N-acetylcysteine (NAC) (Fig. 4D, E and Fig. S4A, S4B). Additionally, NAC mitigated the EMT process induced by Drp1 overexpression (Fig. 4F and Fig. S4C). Moreover, NAC attenuated the enhanced wound healing, migration, and invasion capabilities induced by Drp1 overexpression (Fig. 4G and Fig. S4D, 4E). In conclusion, our results indicated that Drp1 promoted ESCC cell metastasis through the ROS-PGC1-α-Nrf1/2 pathway, and NAC can significantly inhibited Drp1-induced ESCC progression.

### MiR-203a-3p can downregulate the expression of Drp1 and impair mitochondrial function in ESCC cells

**Fig. 5:**
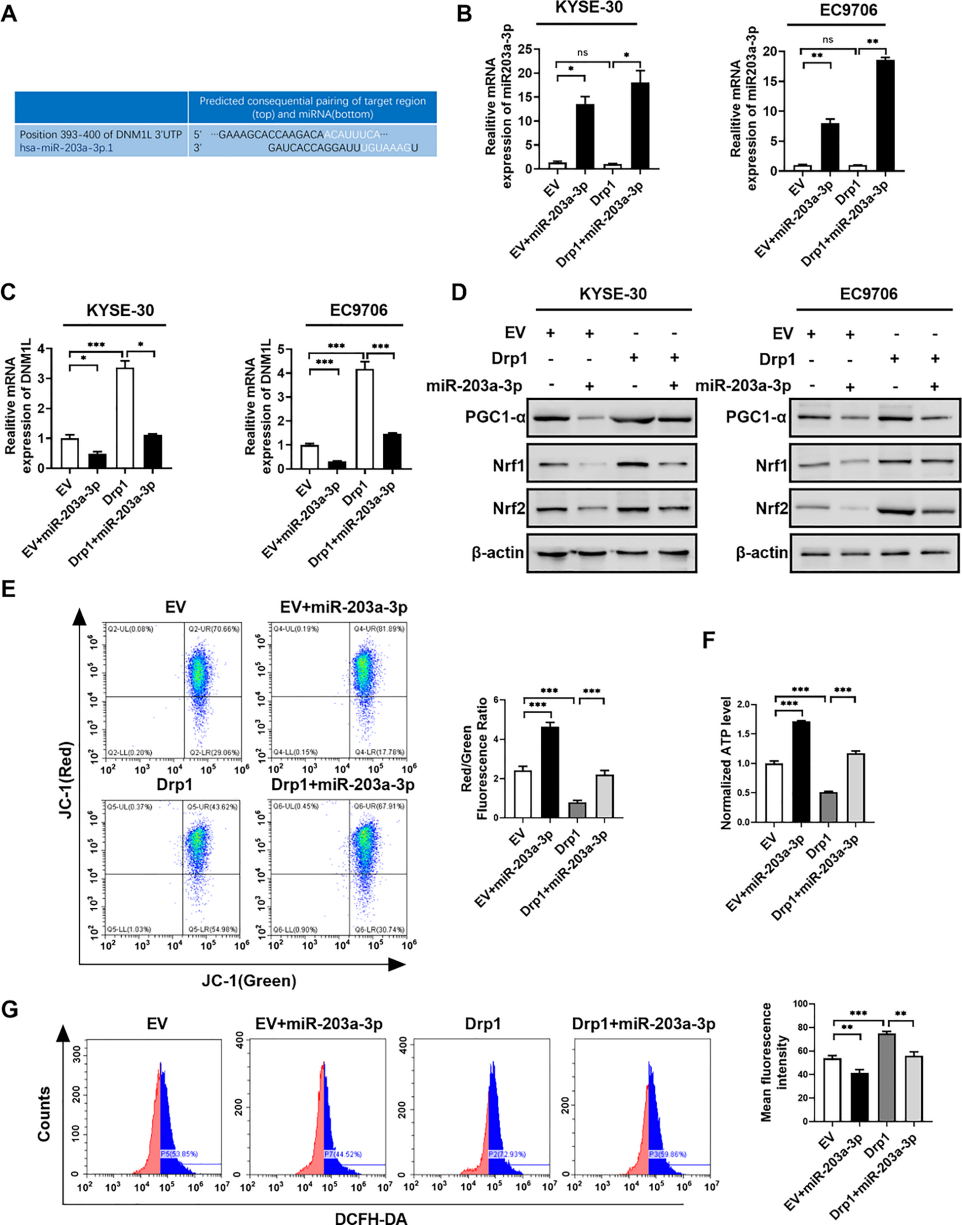
MiR-203a-3p can downregulate the expression of Drp1 and impair mitochondrial function in ESCC cells. **A** TargetScan database analyzed potential target miRNAs of Drp1. **B** and **C** qRT-PCR assay for the levels of miR-203a-3p (**B**) and Drp1 (**C**) in ESCC cells treated with stable overexpression of Drp1 and miR-203a-3p. **D** Western blotting analysis of the levels of PGC1-α, Nrf1, and Nrf2 in ESCC cells treatment as indicated. **E** Representative flow cytometry images of JC-1 in KYSE-30 cells with treatment as indicated. **F** ATP assay kits detect the levels of ATP in KYSE-30 cells. **G** Representative flow cytometry images of ROS in KYSE-30 cells with treatment as indicated. Data information: Data are presented as mean ± SEM, and statistical analysis was performed using One-way ANOVA. p-values were derived from t-tests. *p < 0.05; **p < 0.01; ***p < 0.001

Several studies have underscored the critical involvement of microRNAs (miRNAs) in ESCC pathogenesis [36, 37]. In this investigation, we explored the potential regulatory roles of miRNAs in ESCC progression by targeting Drp1. Through comprehensive bioinformatics analyses using TargetScan database, miR-203a-3p emerged as a prospective miRNA candidate interacting with Drp1 (Fig. 5A). Subsequently, the miR-203a-3p mimic was transfected into ESCC cells overexpressing Drp1 (Fig. 5B). The results showed that the expression of DNM1L was significantly reduced after transfection with miR-203a-3p (Fig. 5C). Moreover, to elucidate the interplay between miR-203a-3p, Drp1, and the ROS-PGC1-α-Nrf1/2 pathway, miR-203a-3p was transfected into Drp1-overexpression ESCC cells. The findings demonstrated that miR-203a-3p effectively attenuated Drp1-induced upregulation of Nrf1/2 (Fig. 5D and Fig. S5).

Furthermore, we investigated the impact of miR-203a-3p on mitochondrial dynamics, specifically focusing on mitochondrial cleavage, reactive oxygen species (ROS) production, ATP generation, and mitochondrial membrane potential. Our results demonstrated that upregulation of Drp1 significantly decreased mitochondrial membrane potential and the level of mitochondrial ATP (Fig. 5E, F), while the levels of ROS was significantly increased in ESCC cells (Fig. 5G). In contrast, miR-203a-3p treatment effectively retarded mitochondrial outer membrane permeabilization and ATP depletion, suppressed ROS production in ESCC cells with Drp1 overexpression (Fig. 5E–G).

Collectively, these findings highlight the inhibitory potential of miR-203a-3p on Drp1 expression and mitochondrial function, suggesting that miR-203a-3p plays a regulatory role in ESCC cells.

### MiR-203a-3p inhibits the proliferation and invasion of ESCC cells by targeting Drp1

**Fig. 6:**
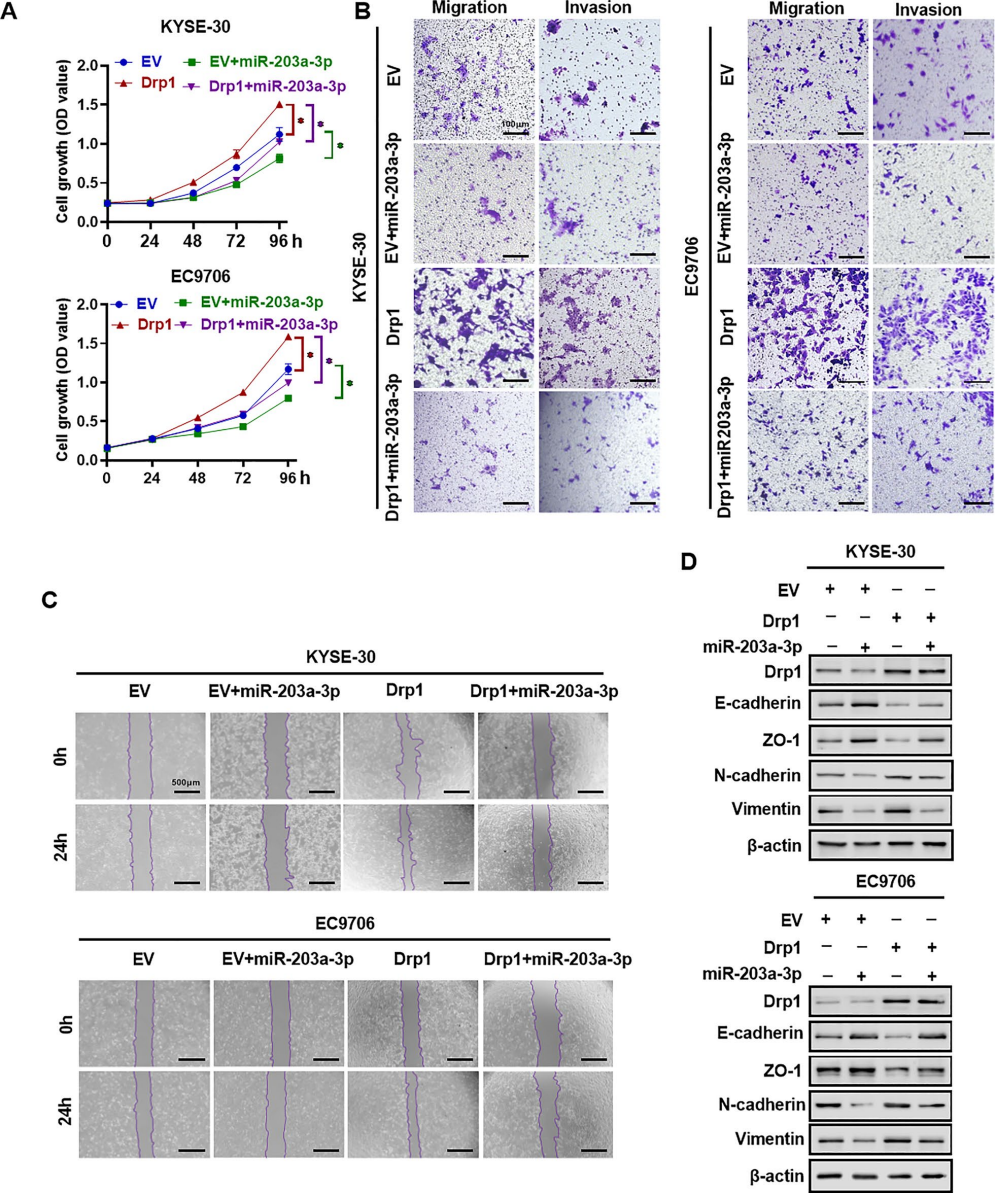
MiR-203a-3p inhibits the proliferation and invasion of ESCC cells by targeting Drp1. **A** MTS assay for the proliferation of ESCC cells treatment as indicated. **B** and **C** Wound-healing assay (**B**) and Transwell migration and invasion assays (**C**) for the migration and invasion capacities of ESCC cells treatment as indicated. Scale bars represent 100 μm. **D** Western blotting analysis the levels of EMT-related gene in KYSE-30 and EC9706 cells treatment as indicated. Data information: Graphs present mean ± SEM. Statistical analysis was performed using One-way ANOVA. p-values were obtained from t-tests. *p < 0.05; **p < 0.01; ***p < 0.001

Subsequently, we examined the influence of miR-203a-3p on the progression of ESCC cells. The MTS assay revealed that miR-203a-3p effectively attenuated the proliferative capacity of ESCC cells mediated by Drp1 (Fig. 6A). Additionally, transwell and wound healing assays demonstrated that increased levels of miR-203a-3p inhibited the migration and invasion capabilities of ESCC cells driven by Drp1 overexpression (Fig. 6B¸ C and Fig. S6A, S6B). Furthermore, western blotting assays indicated that miR-203a-3p suppressed the processes of EMT in ESCC cells induced by Drp1 overexpressing (Fig. 6D and Fig. S6C). In conclusion, these findings underscore the crucial roles of miR-203a-3p in inhibiting the Drp1-mediated malignant progression of ESCC cells.

## Discussion

ESCC is the predominant subtype of esophageal cancer and poses a significant public health challenge. Early diagnosis is essential for the effective therapeutic management of ESCC. During malignant transformation, the levels of various cellular components may change, some of which can be utilized for tumor detection and for monitoring malignant status and prognosis. Research has identified proteins, lncRNA, and miRNA as potential biomarkers for ESCC [38]. These markers hold potential value in the diagnosis, prognosis assessment, and treatment of ESCC, while, the clinical application requires further validation. Therefore, there is an urgent need to continue exploring additional potential markers for ESCC.

**Fig. 7:**
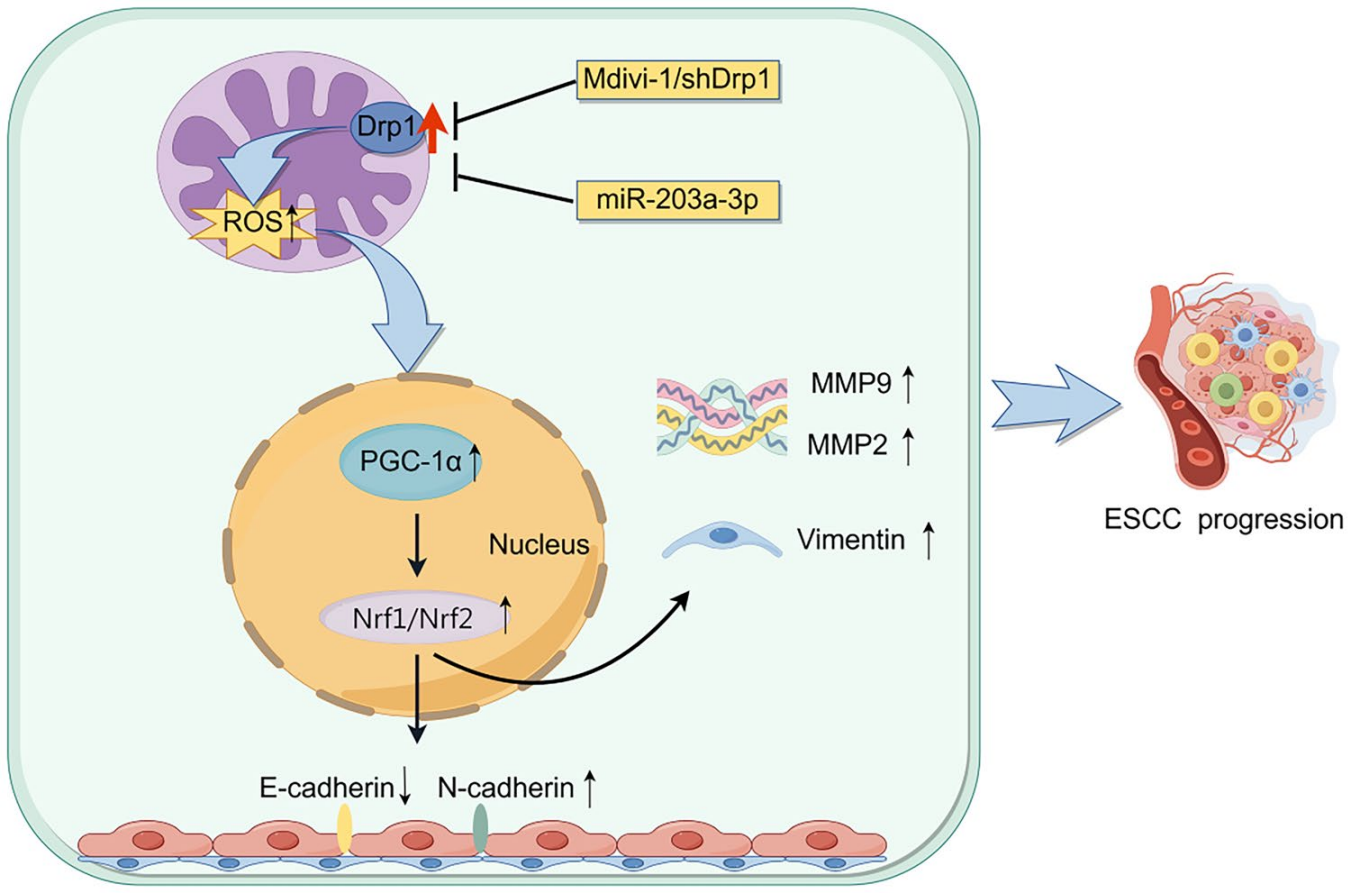
Schematic depicting the effect of targeting Drp1 inhibits ESCC progression via the ROS-PGC1-α-Nrf1/2 pathway

Mitochondrial dysregulation is intricately linked to tumorigenesis, with mitochondrial dynamics, regulated by the highly conserved GTPase gene Drp1, influencing mitochondrial fission. However, the role of Drp1 in ESCC metastasis has not been systematically elucidated. In this study, we demonstrate that high expression levels of Drp1 correlate with poor prognosis in ESCC patients. Drp1 overexpression significantly elevates ROS and the expression levels of PGC1-α and Nrf1/2, which subsequently promote ESCC cancer growth (Fig. 7).

Mitochondria are dynamic organelles that undergo coordinated cycles of fusion and fission, facilitating adaptations to both cellular and extracellular cues [39]. Several studies have shown that the abnormal expression of Drp1 enhances glutaminolysis, aerobic glycolysis, fatty acid metabolism and mitochondrial fission, contributing to the progression of neck cancer, anaplastic thyroid cancer, and colon cancer [39–42]. Previous results have shown that silencing of Drp1 abolished FAL1-induced apoptosis through a mitochondrial-dependent pathway in ESCC cells [43]. Furthermore, our study demonstrates that increased Drp1 promotes autophagy and ESCC progression via the mtDNA stress-mediated cGAS-STING pathway [44]. Consistent with previous studies, we observed that elevated levels of Drp1 are associated with poorer prognosis, correlating with increased expression of Drp1 in ESCC tissues. Furthermore, Drp1 not only enhances the proliferation of ESCC cells but also facilitates their metastasis, both in vitro and in vivo, although the specific mechanisms underlying these effects remain to be fully elucidated.

ROS play pivotal roles in cancer cell signalling, proliferation, apoptosis modulation, and facilitation of invasion and migration processes [45]. Elevated ROS levels activate fission proteins and enhance Drp1 activity, thereby compromising mitochondrial integrity and augmenting fission dynamics [46]. Consistently, in the present study, our findings indicated that Drp1 overexpression significantly increased the level of ROS and upregulated the expression of PGC1-α, Nrf1, and Nrf2, which enhanced the expression of MMP2 and MMP9, thereby promoting the metastasis of ESCC cells. More importantly, following treatment with NAC, a ROS scavenger, the metastasis of ESCC cells was significantly inhibited, which was mediated by the upregulation of Drp1. Therefore, blockage of Drp1 may be a potential strategy to prevent metastasis in ESCC. Mitochondrial division inhibitor (Mdivi-1), a putative inhibitor of Drp1, prevents mitochondrial fission and thereby restricts cell proliferation across several types of oral squamous cell carcinoma, thyroid cancer, breast cancer and so on [47–49]. Consistent with these findings, our results demonstrated that Mdivi-1 significantly inhibited the metastasis of ESCC cells induced by Drp1 overexpression by targeting the suppression of the EMT process and the expression levels of MMP2 and MMP9 in ESCC cells, both in vivo and in vitro.

MicroRNAs (miRNAs) are short, noncoding, single-stranded RNA molecules that regulate gene expression at the post-transcriptional level by binding to mRNAs. miRNAs affect the course of processes of fundamental importance for the proper functioning of the organism, including cell division, proliferation, differentiation, cell apoptosis and so on [50]. Specifically, miRNAs modulate mitochondrial functions that are crucial for cancer biology, including aerobic glycolysis, mitochondrial DNA transcription, apoptosis, and metabolism [51]. Several studies have demonstrated that the expression levels of tumor suppressor miRNAs in ESCC tissues are significantly reduced [52]. Other studies have indicated that miR-203a-3p is associated with poor prognosis in soft tissue sarcoma, colorectal cancer [22, 23] and influences the growth and metastasis of non-small cell lung cancer, pancreatic cancer, hepatocellular carcinoma, and ovarian cancer [24–27]. In the present study, we observed that miR-203a-3p can target and regulate the expression of Drp1 in ESCC tissues, thereby inhibiting the growth and metastasis of ESCC cells, although the specific mechanism remains to be elucidated. However, the interaction between miRNAs and Drp1 remains unexplored. Notably, using online prediction tools, we identified miR-203a-3p as a potential regulator of Drp1, and transfection experiments confirmed that miR-203a-3p inhibited the expression of Drp1, thereby suppressing the metastasis of ESCC cells. Mechanistically, miR-203a-3p inhibited ESCC cell metastasis primarily by obstructing the process of EMT. Interestingly, analysis of DeepSeek (https://chat.deepseek.com/) indicated that Drp1 was significantly upregulated in ESCC tissues and played a crucial role in the progression of ESCC by activating several related signaling pathways, including PI3K/AKT/mTOR, MAPK/ERK, Wnt/β-catenin, and Hippo/YAP. These pathways are integral to regulating cell proliferation, metabolic reprogramming, and immune evasion, all of which contribute to increased tumor aggressiveness and therapeutic resistance, ultimately affecting patient prognosis. However, the precise mechanisms by which miR-203a-3p reduces Drp1 expression and influences mitochondrial metabolic levels remain poorly understood and warrant further investigation.

Collectively, these findings suggested that Drp1 overexpression significantly promoted the progression of ESCC cells by enhancing the EMT process and activating the PGC1-α-Nrf1/2 pathway, thereby facilitating metastasis. Additionally, targeting Drp1 expression with Mdivi-1 or miR-203a-3p can significantly inhibited the metastasis of ESCC cells both in vitro and in vivo through the ROS-PGC1-α-Nrf1/2 pathway. Therefore, targeted suppression of Drp1 levels represents a promising therapeutic strategy for cancer patients. These findings provide novel insights into potential treatments aimed at targeting ESCC metastasis.

## Supplementary Information

Additional file 1. Table S1. Clinical characteristics of ESCC patients. Table S2. Lentivirus and sh lentivirus target sequences. Table S3. QRT-PCR primers used in this study. Table S4. Primary antibodies used for Western blotting and immunohistochemistry. Table S5. The catalogue number and company name of the reagent used in this study. Figure S1. Construction of ESCC cell lines with stable knockdown and overexpression of Drp1. **A** Quantitative analysis for the expression of Drp1 in paired ESCC tissues. **B**–**E** Western blottingand qRT-PCRanalysis for the expression of Drp1 in KYSE-30 and EC9706 cells with Drp1 stable overexpression and control cells. EV, empty vector; Drp1, expression vector encoding Drp1. **F** and **G** Western blottingand qRT-PCRanalysis for the expression of Drp1 in KYSE-70 cells with Drp1 stable knockdown and control cells. shCtrl, control shRNA; shDrp1, shRNA against Drp1. Data information: Graphs show mean ± SEM, two-tailed unpaired t-test. p-value from t tests. *p < 0.05; **p < 0.01; ***p < 0.001. Figure S2. Drp1 overexpression promotes metastasis of ESCC cells in vitro and in vivo. **A** Quantitative analysis the migration of Wound-healing assay in Fig. 2A. **B** Quantitative analysis the number of migrated and invaded cells of Transwell assay in Fig. 2B. **C**, **D** Quantitative analysis for the levels of EMT-related proteins in KYSE-30 and EC9706cellsin Fig. 2C. **E** Representative images of IHC staining of MMP2 and Drp1and levelsin paired ESCC tissues. Figure S3. Targeting Drp1 inhibits the progression of ESCC cells. **A**–**C** Quantitative analysis for the levels of EMT-related proteins in ESCC cells as indicated.. shCtrl, control shRNA; shDrp1, shRNA against Drp1; EV, empty vector; Drp1, expression vector encoding Drp1. Data information: Graphs show mean ± SEM, One - way ANOVA. p-value from t tests. *p < 0.05; **p < 0.01; ***p < 0.001. Figure S4. Drp1 accelerates ESCC cell metastasis through the ROS-PGC1-α-Nrf1/2 pathway. **A**–**C** Quantitative analysis for the levels of proteins in in Fig. 4D–F. **D** Wound-healing migration assay for ESCC cells treatment as indicated. Scale bars: 500 μm. **E** Quantitative analysis the number of migrated and invaded cells of Transwell assay in Fig. 4G. Data information: Data information: Graphs show mean ± SEM, One-way ANOVA. p-value from t tests. *p < 0.05; **p < 0.01; ***p < 0.001. Figure S5. MiR-203a-3p suppress the expression of Drp1 via targeting ROS-PGC1-α-Nrf1/2 axis. **A** Quantitative analysis for the levels of proteins in ESCC cells as indicated.. Data information: Graphs show mean ± SEM, One - way ANOVA. p-value from t tests. *p < 0.05; **p < 0.01; ***p < 0.001. Figure S6. MiR-203a-3p inhibits the proliferation and invasion of ESCC cells by targeting Drp1. **A** Quantitative analysis the number of migrated and invaded cells of Transwell assay in Fig. 6C. **B** Quantitative analysis the migration of Wound-healing assay in Fig. 6B. **C** Quantitative analysis for the levels of proteins in ESCC cells as indicated.. Data information: Graphs show mean ± SEM, One-way ANOVA.* p* value from t tests. *p < 0.05; **p < 0.01; ***p < 0.001.

Additional file 2

## Data Availability

All data are available in the main text or the Supplementary Materials.

## Abbreviations

Drp1: Dynamin-related protein 1
EMT: Epithelial-mesenchymal transition
ESCC: Esophageal squamous cell carcinoma
ROS: Reactive oxygen species
PGC1-α: Peroxisome proliferator-activated receptor gamma coactivator-1 alpha
Nrf1: Nuclear factor E2 related factor 1
Nrf2: Nuclear factor E2 related factor 2
GTPase: Guanosine triphosphatase
miRNAs: MicroRNAs
IHC: Immunohistochemistry
HRP: Horseradish peroxidase
EdU: Ethynyl deoxyuridine
SPF: Specific pathogen-free
TCGA: The Cancer Genome Atlas
H&E: Hematoxylin and eosin
NAC: N-acetylcysteine
sncRNAs: Small non-coding RNAs
tRF: Derived fragments
rRF: RRNA-derived fragments
piRNA: Piwi-interacting RNAs
lncRNA: Long non-coding RNAs
qRT-PCR: Real-Time quantitative Polymerase Chain Reaction
Mdivi-1: Mitochondrial division inhibitor 1
shRNA: Short hairpin RNA

## Declarations

### Animal studies

Six-week-old male BALB/c-nude mice were procured from Beijing Vital River Laboratory Animal Technology Co., Ltd., and maintained under specific pathogen-free (SPF) conditions in the Experimental Animal Center of Henan University. All experimental procedures involving animals were approved by the Henan University Animal Care Committee.
