# Supplementary material for: Targeting Drp1 inhibits ESCC progression via the ROS-PGC1-α-Nrf1/2 pathway
Source: J Transl Med. 2025 Jun 17;23:674. doi: 10.1186/s12967-025-06697-8 (PMC12175380; doi:10.1186/s12967-025-06697-8)
Supplement: Supplementary file 1 — Additional file 1. Table S1. Clinical characteristics of ESCC patients. Table S2. Lentivirus and sh lentivirus target sequences. Table S3. QRT-PCR primers used in this study. Table S4. Primary antibodies used for Western blotting and immunohistochemistry. Table S5. The catalogue number and company name of the reagent used in this study. Figure S1. Construction of ESCC cell lines with stable knockdown and overexpression of Drp1. A Quantitative analysis for the expression of Drp1 in paired ESCC tissues. B–E Western blottingand qRT-PCRanalysis for the expression of Drp1 in KYSE-30 and EC9706 cells with Drp1 stable overexpression and control cells. EV, empty vector; Drp1, expression vector encoding Drp1. F and G Western blottingand qRT-PCRanalysis for the expression of Drp1 in KYSE-70 cells with Drp1 stable knockdown and control cells. shCtrl, control shRNA; shDrp1, shRNA against Drp1. Data information: Graphs show mean ± SEM, two-tailed unpaired t-test. p-value from t tests. *p < 0.05; **p < 0.01; ***p < 0.001. Figure S2. Drp1 overexpression promotes metastasis of ESCC cells in vitro and in vivo. A Quantitative analysis the migration of Wound-healing assay in Fig. 2A. B Quantitative analysis the number of migrated and invaded cells of Transwell assay in Fig. 2B. C, D Quantitative analysis for the levels of EMT-related proteins in KYSE-30 and EC9706cellsin Fig. 2C. E Representative images of IHC staining of MMP2 and Drp1and levelsin paired ESCC tissues. Figure S3. Targeting Drp1 inhibits the progression of ESCC cells. A–C Quantitative analysis for the levels of EMT-related proteins in ESCC cells as indicated.. shCtrl, control shRNA; shDrp1, shRNA against Drp1; EV, empty vector; Drp1, expression vector encoding Drp1. Data information: Graphs show mean ± SEM, One - way ANOVA. p-value from t tests. *p < 0.05; **p < 0.01; ***p < 0.001. Figure S4. Drp1 accelerates ESCC cell metastasis through the ROS-PGC1-α-Nrf1/2 pathway. A–C Quantitative analysis for the levels of proteins [file 12967_2025_6697_MOESM1_ESM.docx]

**Supplementary materials:**

**Targeting Drp1 inhibits ESCC progression via the ROS-PGC1-α-Nrf1/2 pathway**

**Supplementary Tables**

**Table 1. Clinical characteristics of ESCC patients.**

| **Variable** | **All patients, n (%), Total=102** |
| --- | --- |
| Sex |  |
| Female | 23 (22.55%) |
| Male | 79 (77.45%) |
| Age，years |  |
| <60 | 35 (34.31%) |
| ≥60 | 67 (65.69%) |
| T classification |  |
| T1 | 19 (18.63%) |
| T2 | 36 (33.33%) |
| T3 | 47 (43.52%) |
| N classification |  |
| Absent (0) | 69 (67.65%) |
| Present (1/2/3) | 33 (32.35%) |
| M classification |  |
| M0 | 101 (99.02%) |
| M1 | 1 (0.98%) |
| Histological grade |  |
| G1 | 33 (32.35%) |
| G2 | 63 (61.76%) |
| G3 | 6 (5.88%) |
| Size of tumor, cm |  |
| <5cm | 21 (20.59%) |
| ≥5cm | 81 (79.41%) |
| Tumor location |  |
| Lower thoracic esophagus | 37 (36.27%) |
| Middle thoracic esophagus | 48 (47.06%) |
| Upper thoracic esophagus | 17 (16.67%) |
| Survival |  |
| Death | 31 (30.39%) |
| Alive | 71 (69.61%) |

**Table 2. Lentivirus and sh lentivirus target sequences.**

| **Gene** | **Forward Primer** | **Reverse Primer** |
| --- | --- | --- |
| Drp1 | GGACCGGTTCTAGAGCCACCATGGAGGCGCTAATTCCTGT | TCTGGAACATCGTATGGGTACCAAAGATGAGTCTCCCGGA |
| Drp1 siRNA | ACUAUUGAAGGAACUGCAAA | UAUAUUUUGCAGUUCCUUCA |

**Table 3. QRT-PCR primers used in this study.**

| **Gene** | **Forward Primer** | **Reverse Primer** |
| --- | --- | --- |
| Drp1 | AAGGAGCCAGTCAAATTATTGC | AGTCAACAAAGTCTCAGTATTA |
| GAPDH | AACGGATTTGGTCGTATTGG | TTGATTTTGGAGGGATCTCG |
| miR-203a-3p | GCGCCGTGAAATGTTTAGG |  |
| N-cadherin | TGGACCATCACTCGGCTTA | ACACTGGCAAACCTTCACG |
| Vimentin | CCTGAACCTGAGGGAAACTAA | GCAGAAAGGCACTTGAAAGC |
| ZO-1 | CACGCAGTTACGAGCAAG | TGAAGGTATCAGCGGAGG |

**Table 4. Primary antibodies used for Western blotting and immunohistochemistry.**

| **Antibody** | **Company (Cat.No.)** | **Dilutability** |
| --- | --- | --- |
| Actin | Proteintech (60008-1-lg) | WB, 1:10000 |
| Drp1 | Abcam (ab56788) | WB, 1:2000; IHC, 1:200 |
| E-cadherin  MMP2  MMP9 | Proteintech (20874-1-AP)  Proteintech (10373-2-AP)  Proteintech (10375-2-AP) | WB, 1:5000  WB, 1:1000; IHC, 1:200  WB, 1:1000 |
| Mouse IgG | Proteintech (SA00001-1) | WB, 1:5000 |
| N-cadherin | Cell Signaling (#13116) | WB, 1:1000 |
| Nrf1 | Cell Signaling (#46743S) | WB, 1:1000 |
| Nrf2 | Cell Signaling (#12721S) | WB, 1:1000 |
| PGC1-α | Cell Signaling (#2178S) | WB, 1:1000 |
| Rabbit IgG | Proteintech (SA00001-2) | WB, 1:5000 |
| TOM20 | Proteintech (11802-1-AP) | IF, 1:100 |
| Vimentin | Proteintech (10366-1-AP) | WB, 1:2000 |
| ZO-1 | Proteintech (21773-1-AP) | WB, 1:1000 |

**Table 5. The catalogue number and company name of the reagent used in this study.**

| **Reagent** | **Company** | **Cat.No.** |
| --- | --- | --- |
| RIPA | Beyotime | P0013C |
| Protease Inhibitor | Roche | 539128 |
| Phosphatase inhibitor | Roche | 200-664-3 |
| 5x Protein Loading buffer | Solarbio | P1040 |
| BCA Protein Assay Kit | Solarbio | PC0020 |
| 30% Acr-Bis (29:1) | Solarbio | A1010 |
| 1.5MTris.HCL(Ph8.8) | Solarbio | T1010 |
| 1.0MTris.HCL(Ph6.8) | Solarbio | T1020 |
| SDS-PAGE | Solarbio | S8010 |
| PVDF membranes | MILLIPORE | IPVH00010 |
| Ammonium Persulfate | Solarbio | A1030 |
| TEMED | Sigma-aldrich | 110-18-9 |
| Glycine | Solarbio | G8200 |
| TRIS | Solarbio | T8060 |
| Marker | Shanghai Yamei Biotechnology | WJ102 |
| Carbinol | HENGXING CHEMICAL REAGENT | 67-56-1 |
| BSA | Solarbio | A8020 |
| Tween-20 | Solarbio | T8220 |
| 1×TBST | Solarbio | T1085 |
| ECL Plus | Solarbio | PE0010 |
| Membrane regeneration solution | Solarbio | SW3022 |
| Anhydrous ethanol | HENGXING CHEMICAL REAGENT | 64-17-5 |
| Xylene | HENGXING CHEMICAL REAGENT | 1330-20-7 |
| 1×PBST | Solarbio | P1031 |
| Tissue fixative | Servicebio | G1101 |
| Endogenous peroxidase | Wuhan Boster Biological Technology | AR1108 |
| Immunohistochemistry Kit | Sangon Biotech | D601037 |
| Neutral gum | Solarbio | G8590 |
| TRIzol LS Reagent | Thermo Fisher Company | 10296010CN |
| Phenol: Chloroform: isoamyl alcohol | Sigma-aldrich | 136112-00-0 |
| 5X Evo M-MLV RT Master Mix *1 | Accurate Biology | AG11706 |
| SYBR Green Pro Taq HS | Accurate Biology | AG11701 |
| Fetal bovine serum | Biological Industries | 04-001-1ACS |
| Medium 1640 | CORNING | 10-040-CV |
| PBS | Shanghai BasalMedia Technologies | B310KJ |
| Lipofectamine 2000 reagent | Invitrogen | 11668500 |
| MTS | Keygen biotech | KGA9307-500 |
| Cell-light EdU | Guangzhou RiboBio | C10310-1 |
| Crystal violet | Solarbio | 548-62-9 |
| Matrigel | CORNING | 3422 |
| DMSO | Solarbio | D8370 |
| 4% polyformaldehyde | Solarbio | P1110 |
| Mdivi-1 | Sigma | 338967-87-6 |
| Triton X-100 | Solarbio | T8200 |
| Eosin | Solarbio | G1100 |
| Hematoxylin dye | Solarbio | SH8390 |
| DMEM | CORNING | 17-207-CV |
| Puromycin | APE×BIO | A3740 |
| DCFH-DA | Beyotime | S0033S |
| JC-1 dye | Beyotime | C2006 |
| ATP Assay Kit | Beyotime | S0026 |
| Cell cycle kit | Beyotime | C1052 |

**Supplemental Figures**


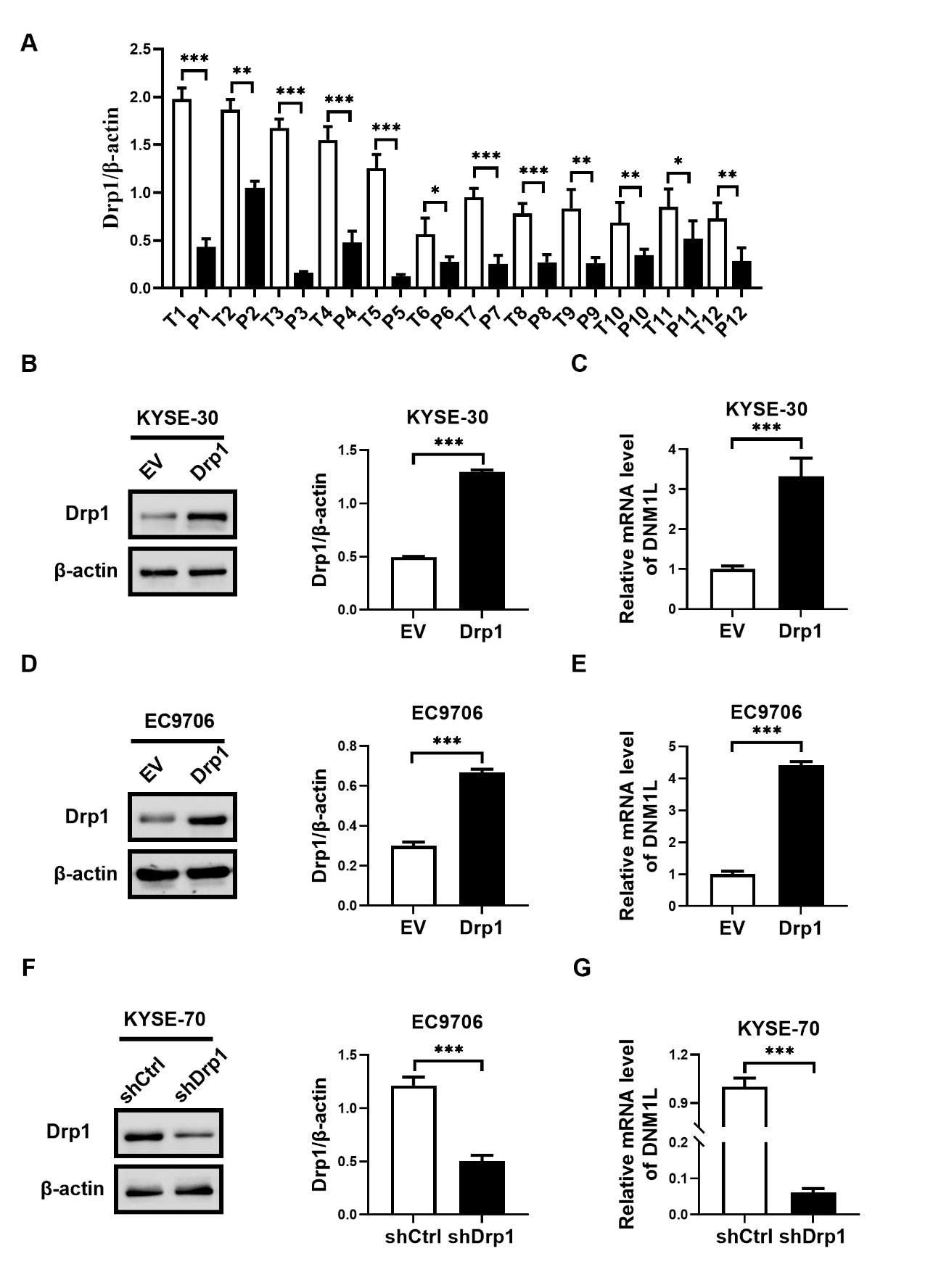


**Figure S1. Construction of ESCC cell lines with stable knockdown and overexpression of Drp1.**

(A). Quantitative analysis for the expression of Drp1 in paired ESCC tissues (n = 3 independent experiments). (B - E) Western blotting (B and D) and qRT-PCR (C and E) analysis for the expression of Drp1 in KYSE-30 and EC9706 cells with Drp1 stable overexpression and control cells (n = 3 independent experiments). EV, empty vector; Drp1, expression vector encoding Drp1. (F and G) Western blotting (F) and qRT-PCR (G) analysis for the expression of Drp1 in KYSE-70 cells with Drp1 stable knockdown and control cells (n = 3 independent experiments). shCtrl, control shRNA; shDrp1, shRNA against Drp1.

Data information: Graphs show mean ± SEM, two - tailed unpaired t - test. p-value from t tests. *, *p* <  0.05; **, *p*  <  0.01; ***, *p*  <  0.001.


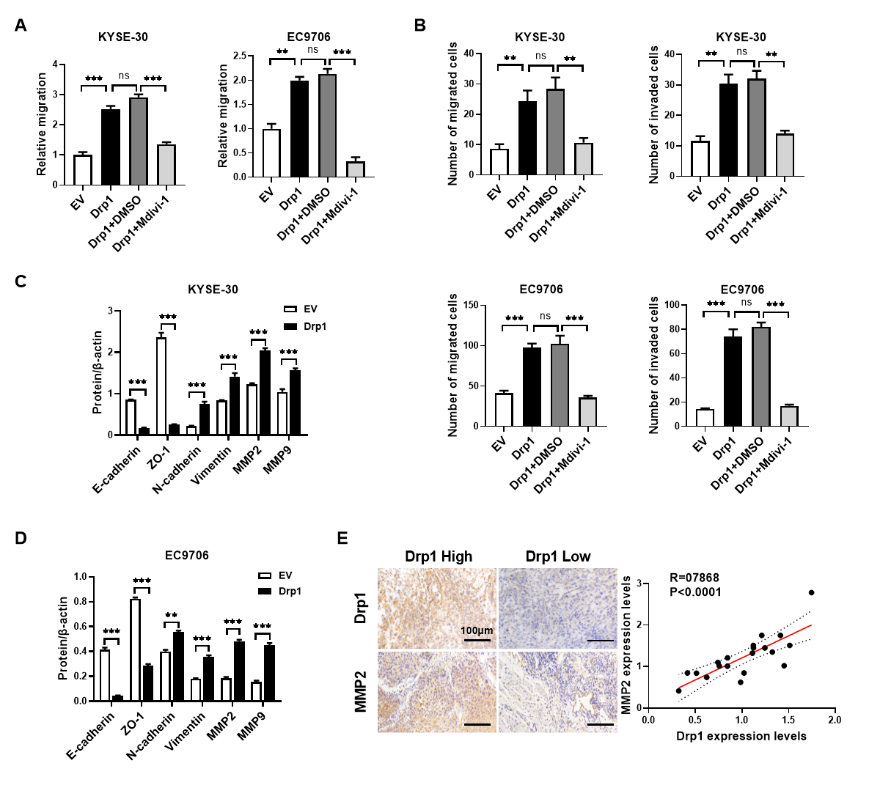


**Figure S2. Drp1 overexpression promotes metastasis of ESCC cells in vitro and in vivo.**

(A). Quantitative analysis the migration of Wound-healing assay in Fig. 2A. (B). Quantitative analysis the number of migrated and invaded cells of Transwell assay in Fig. 2B. (C-D). Quantitative analysis for the levels of EMT - related proteins in KYSE-30 and EC9706 (C) cells (n = 3 independent experiments) in Fig. 2C. (E). Representative images of IHC staining of MMP2 and Drp1 (left panel) and levels (right panel) in paired ESCC tissues.


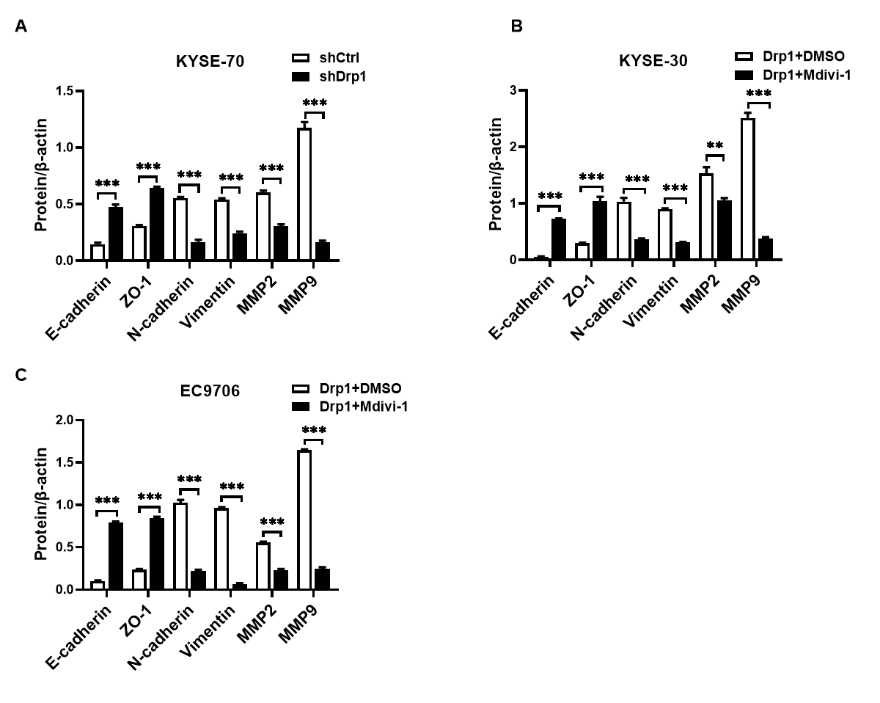


**Figure S3. Targeting Drp1 inhibits the progression of ESCC cells.**

(A-C). Quantitative analysis for the levels of EMT - related proteins in ESCC cells as indicated. (n = 3 independent experiments). shCtrl, control shRNA; shDrp1, shRNA against Drp1; EV, empty vector; Drp1, expression vector encoding Drp1.

Data information: Graphs show mean ± SEM, One - way ANOVA. p-value from t tests. *, *p*  <  0.05; **, *p*  <  0.01; ***, *p*  <  0.001.


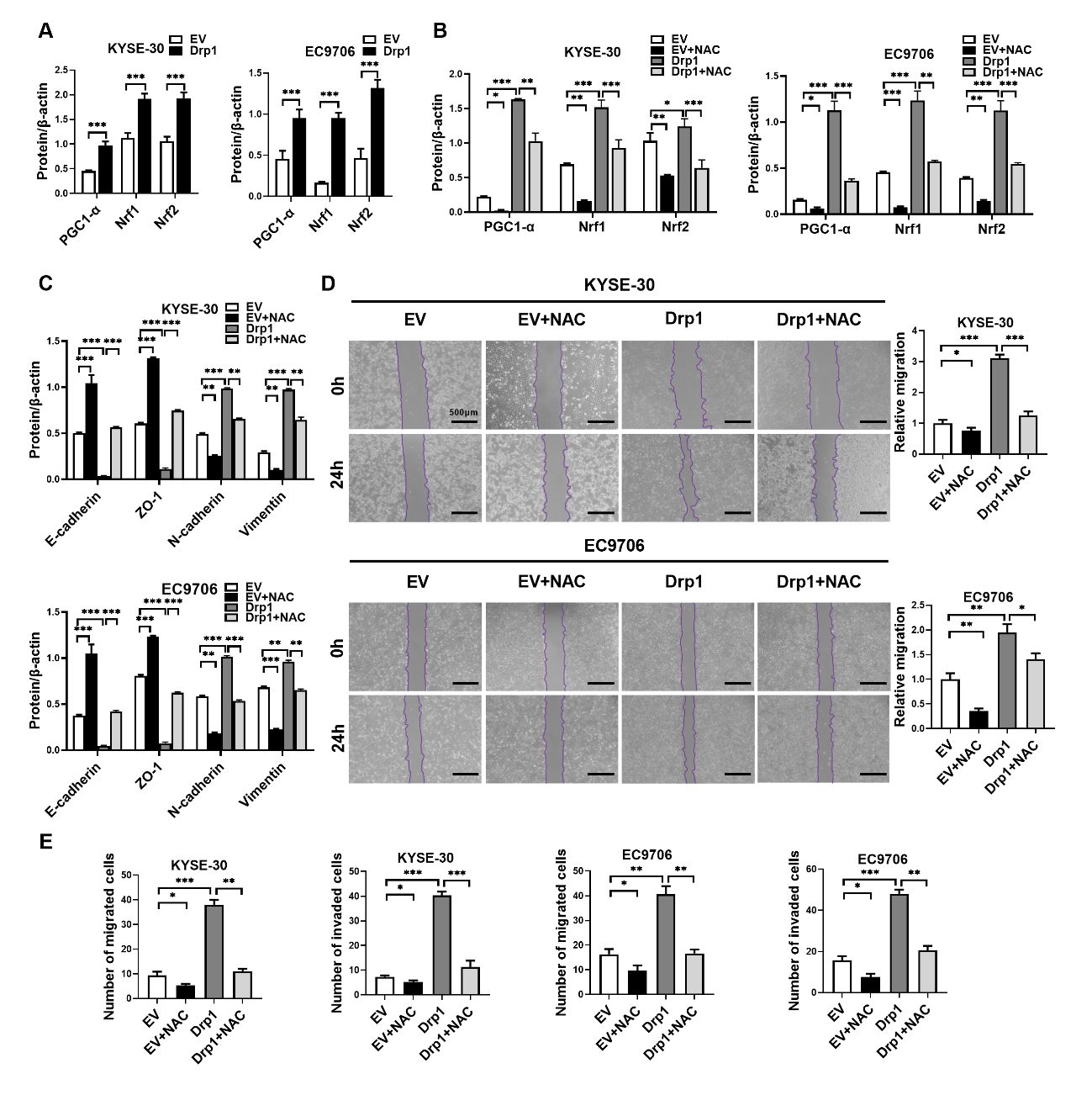


**Figure S4. Drp1 accelerates ESCC cell metastasis through the ROS-PGC1-α-Nrf1/2 pathway.**

(A-C). Quantitative analysis for the levels of proteins in in Fig. 4D – 4F. (D). The migration of Wound-healing assay. (E). Quantitative analysis the number of migrated and invaded cells of Transwell assay in Fig. 4G.

Data information: Data information: Graphs show mean ± SEM, One - way ANOVA. p-value from t tests. *, *p*  <  0.05; **, *p*  <  0.01; ***, *p*  <  0.001.


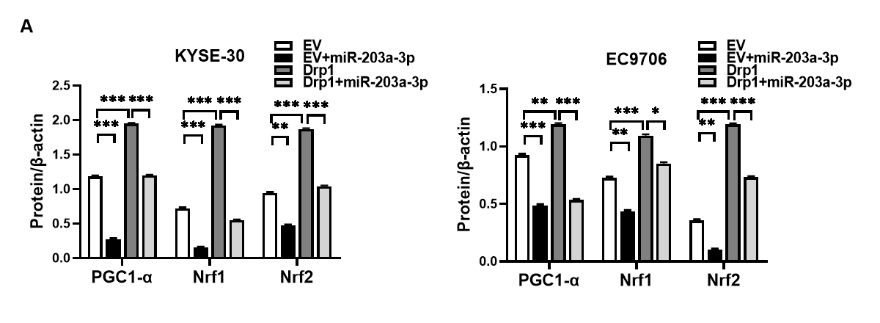


**Figure S5. MiR-203a-3p suppress the expression of Drp1 via targeting ROS-PGC1-α-Nrf1/2 axis.**

(A). Quantitative analysis for the levels of proteins in ESCC cells as indicated. (n = 3 independent experiments).

Data information: Graphs show mean ± SEM, One - way ANOVA. p-value from t tests. *, *p*  <  0.05; **, *p*  <  0.01; ***, *p*  <  0.001.


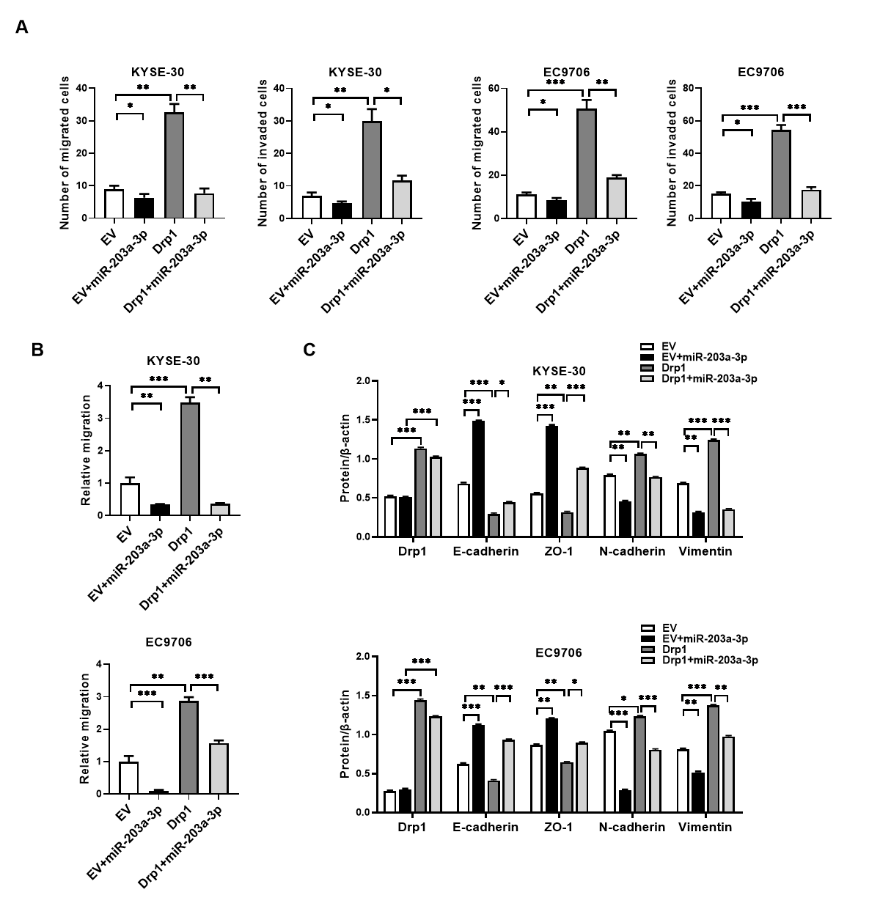


**Figure S6. MiR-203a-3p inhibits the proliferation and invasion of ESCC cells by targeting Drp1.**

(A). Quantitative analysis the number of migrated and invaded cells of Transwell assay in Fig. 6C. (B). Quantitative analysis the migration of Wound-healing assay in Fig. 6B. (C). Quantitative analysis for the levels of proteins in ESCC cells as indicated. (n = 3 independent experiments).

Data information: Graphs show mean ± SEM, One - way ANOVA. *p* value from t tests. *, *p*  <  0.05; **, *p*  <  0.01; ***, *p*  <  0.001.
