# Supplementary material for: Targeting Drp1 inhibits ESCC progression via the ROS-PGC1-α-Nrf1/2 pathway
Source: J Transl Med. 2025 Jun 17;23:674. doi: 10.1186/s12967-025-06697-8 (PMC12175380; doi:10.1186/s12967-025-06697-8)

**Supplementary materials:**

**RNA concentration and the OD260/280 values for RNA quality control.**

**Figure 1E.**

**T1:**


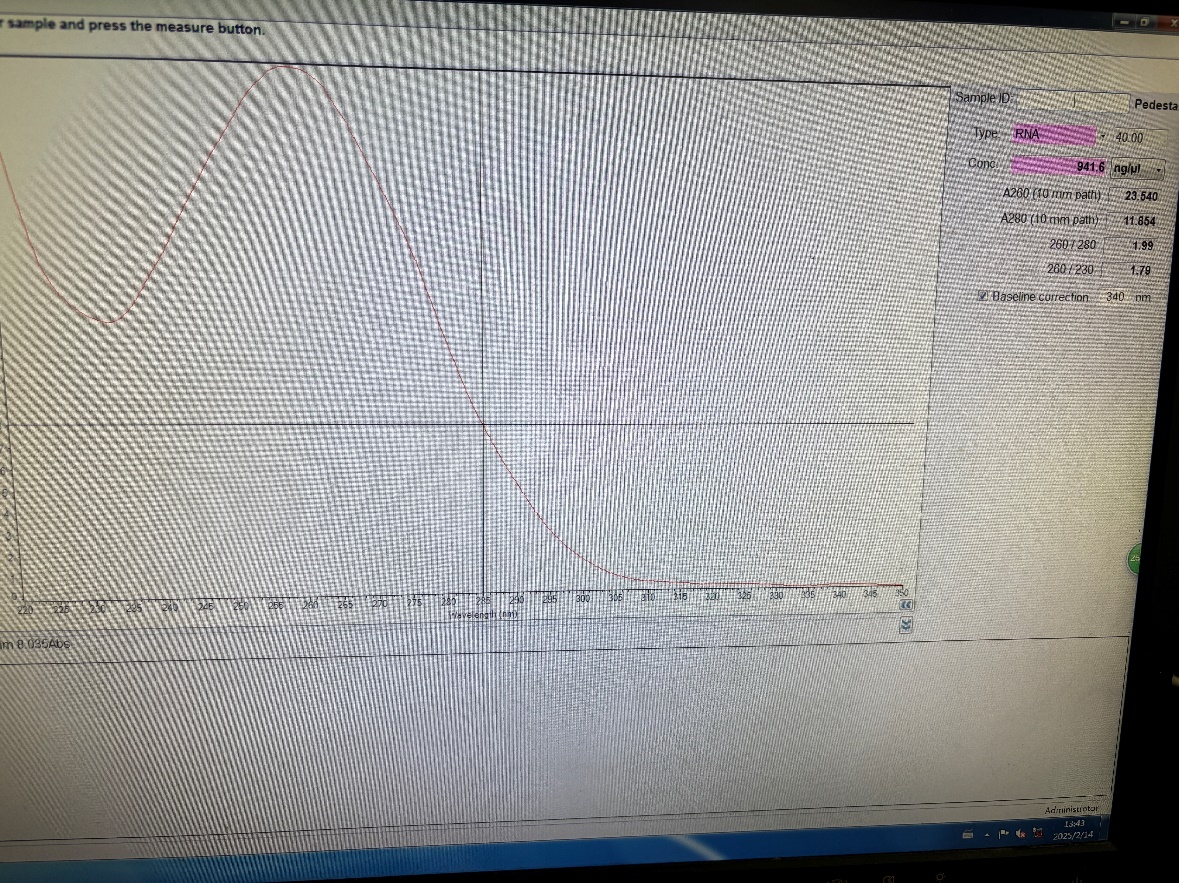


**P1:**


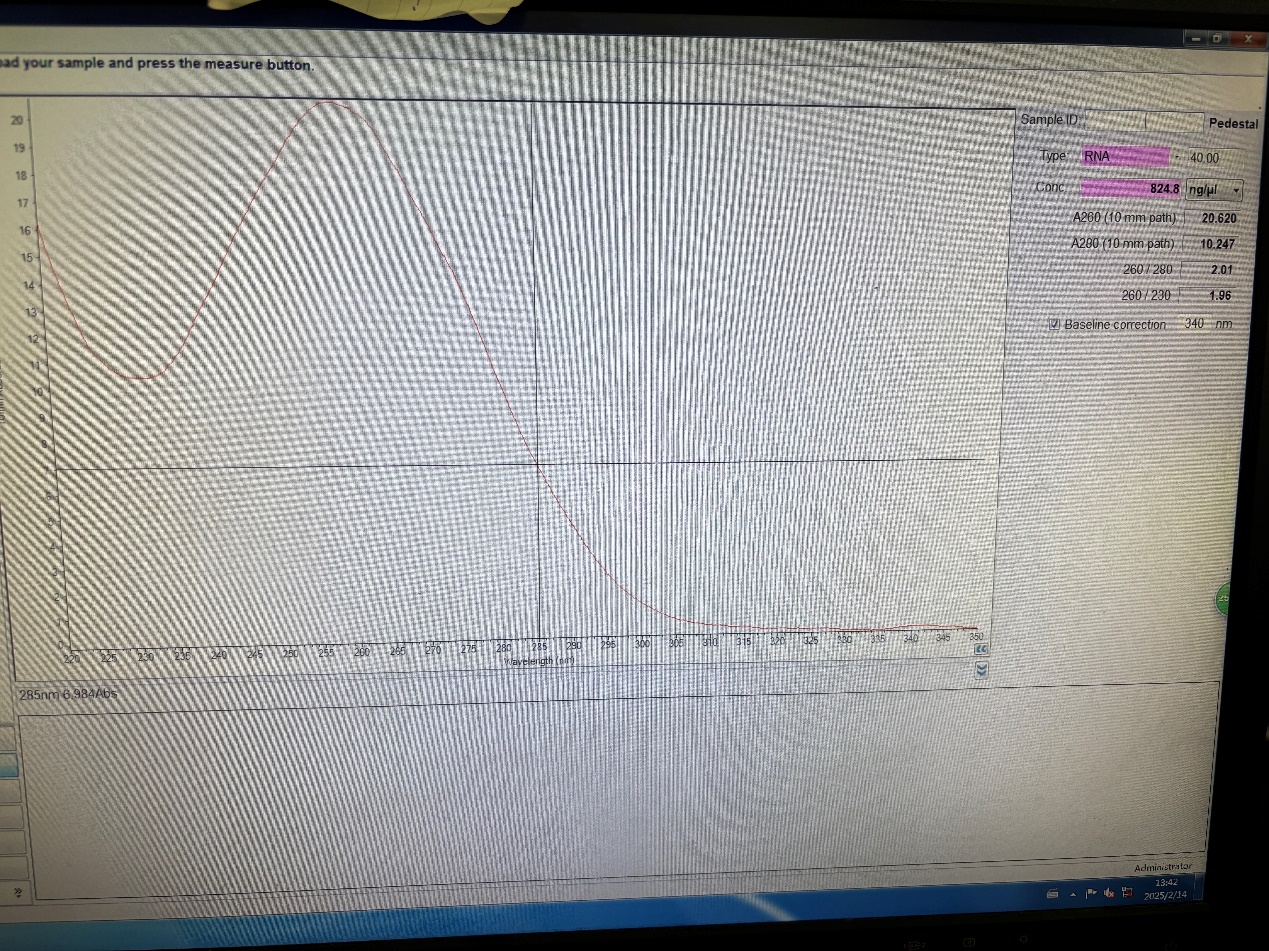


**T2:**


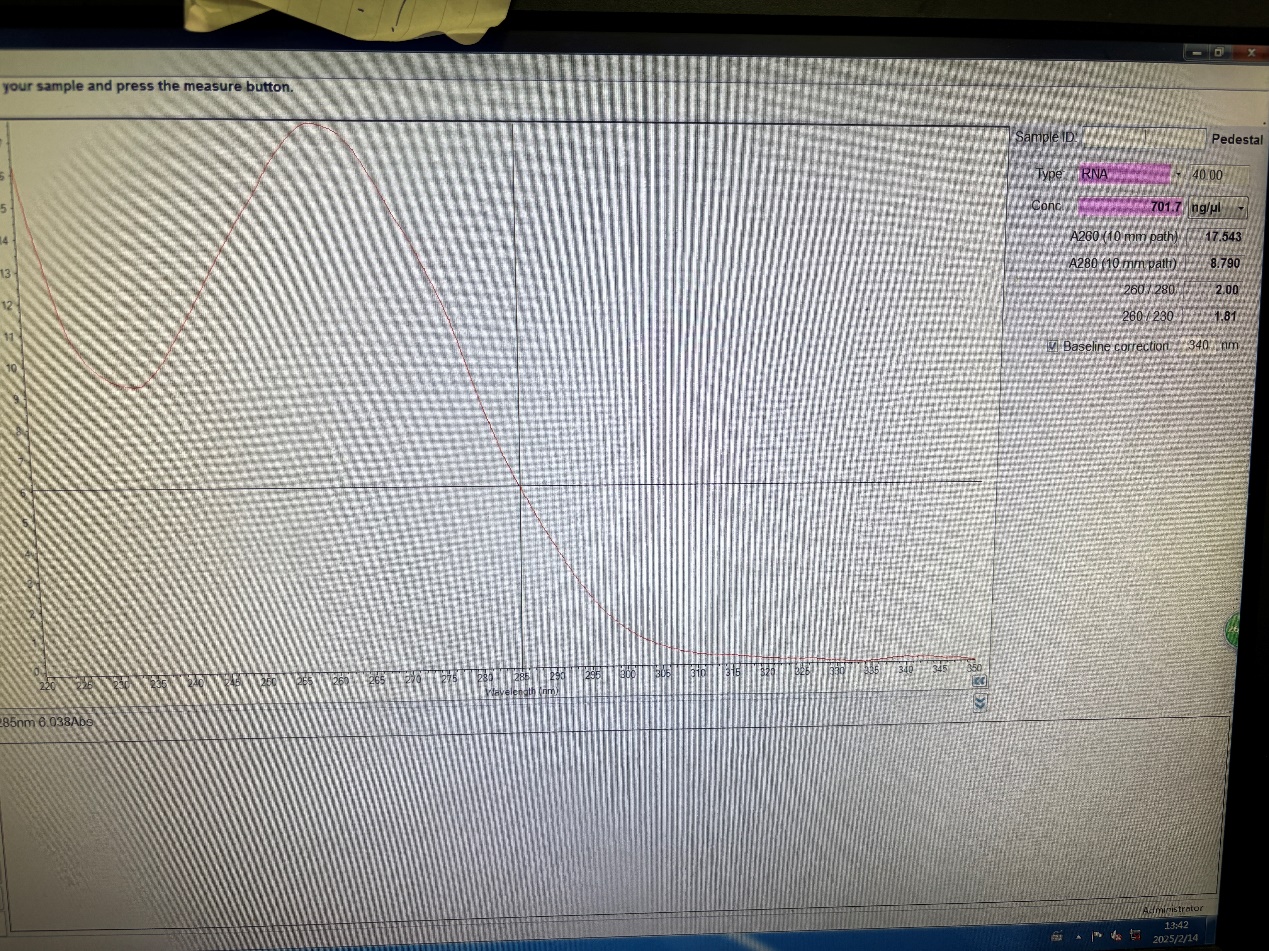


**P2:**


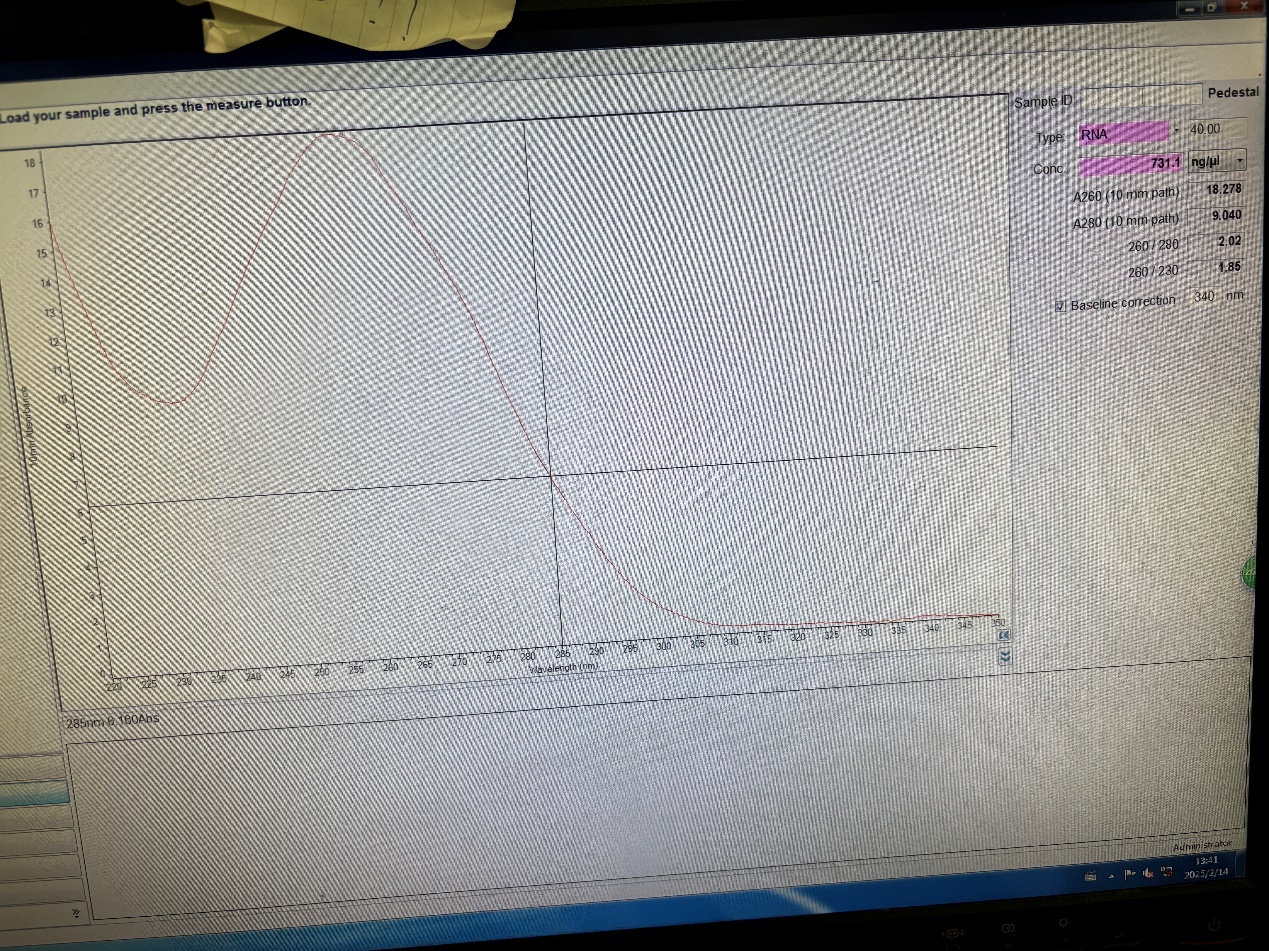


**T3:**


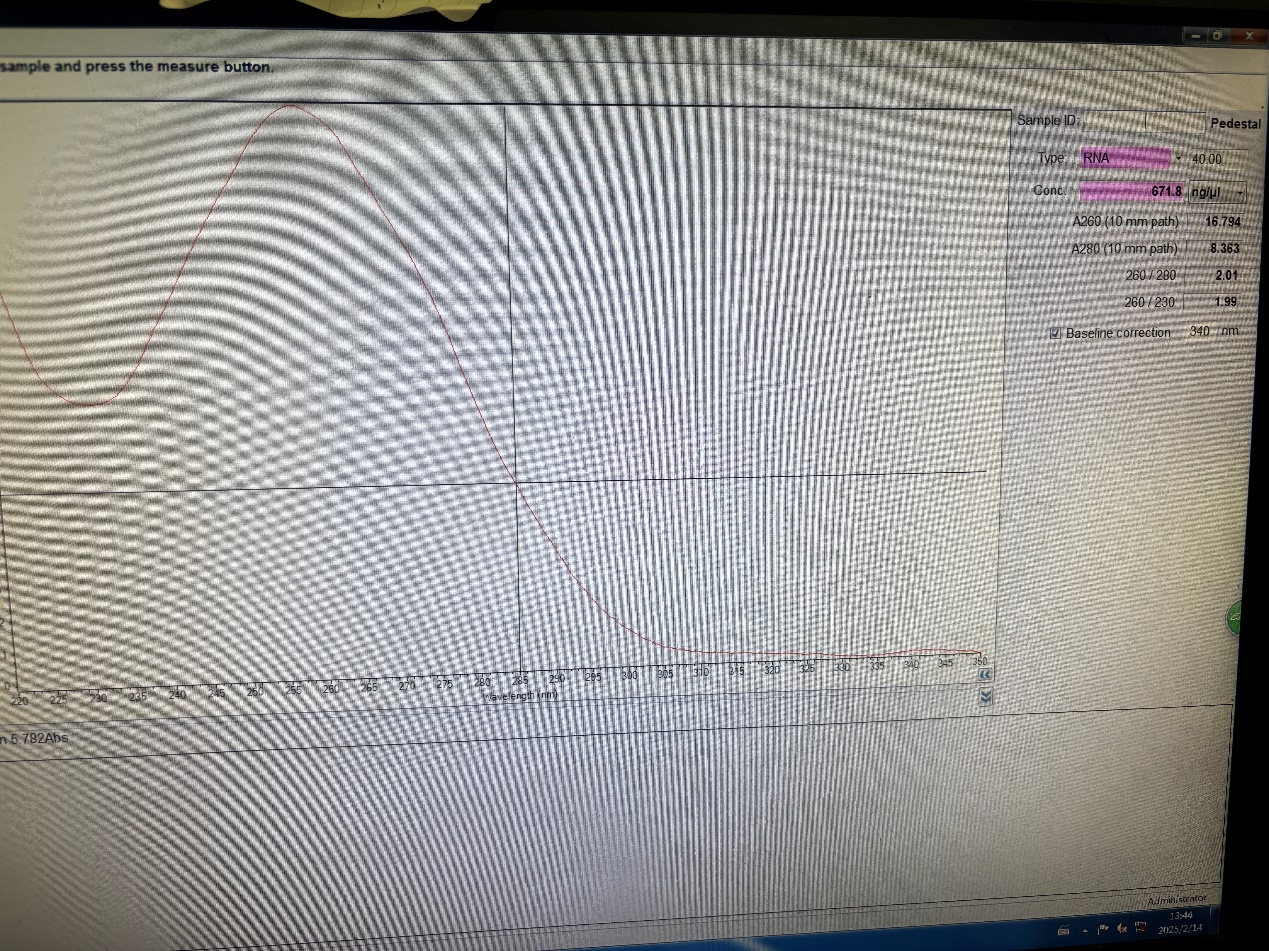


**P3:**


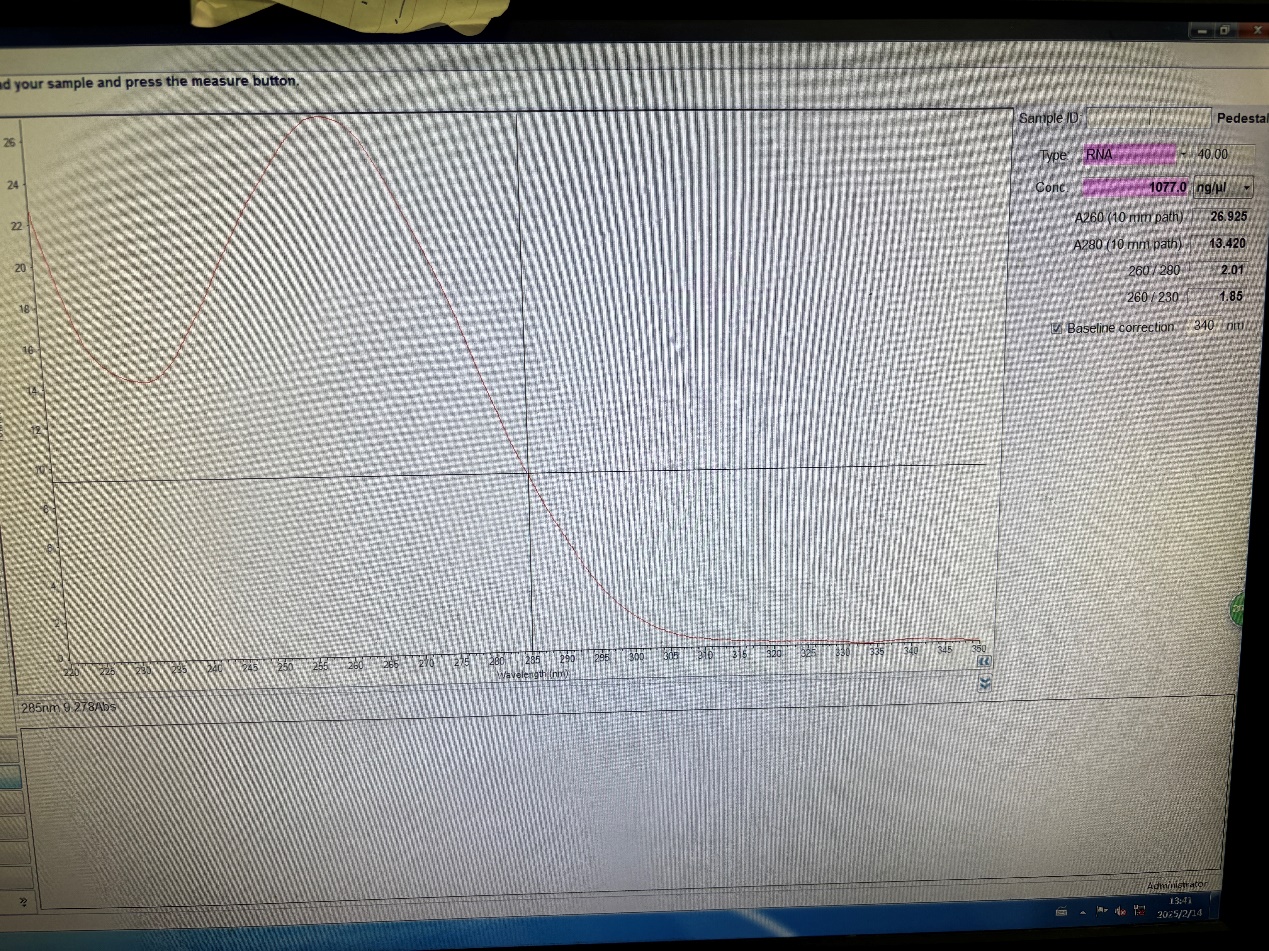


**T4:**


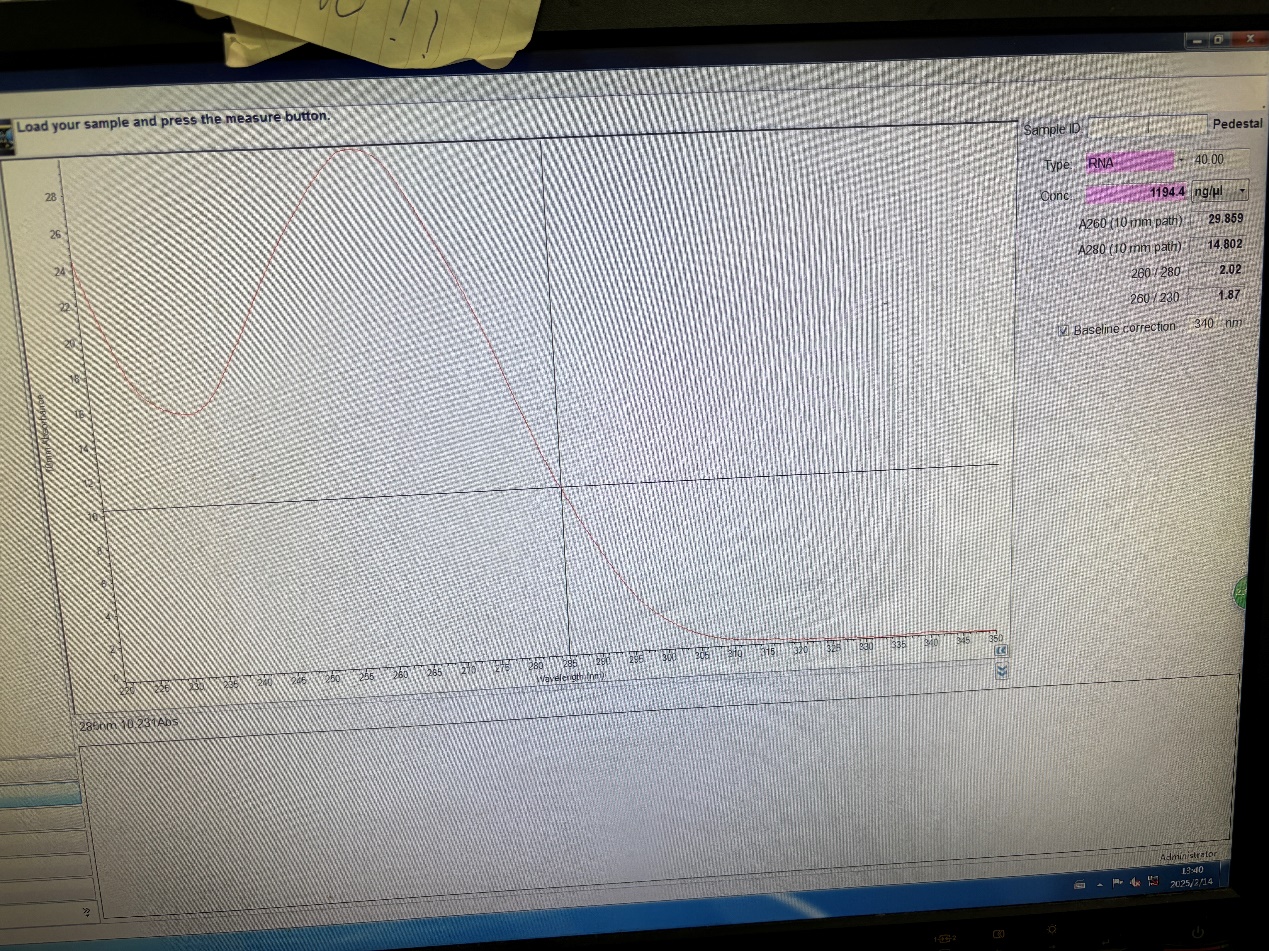


**P4:**


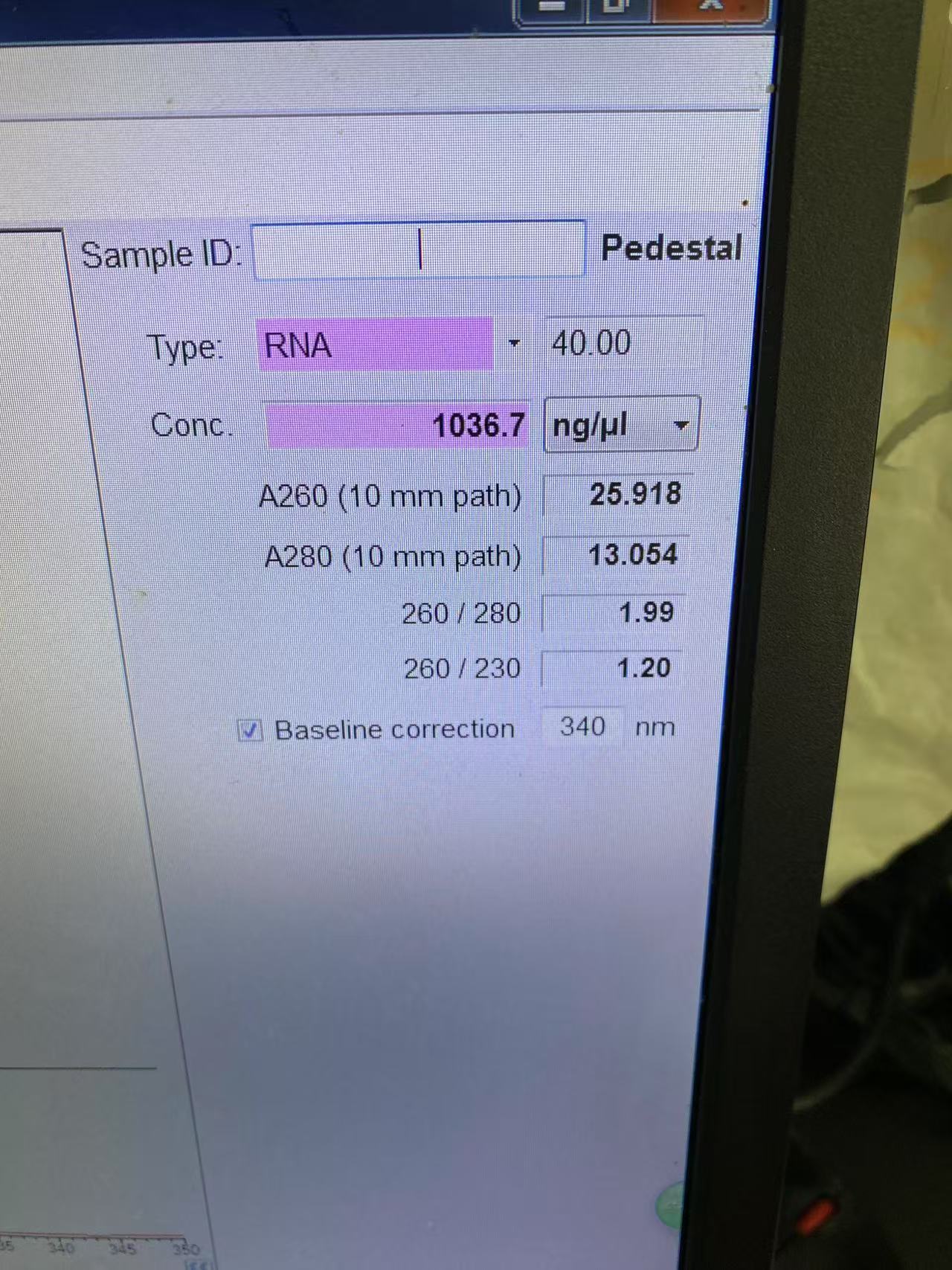


**T5:**


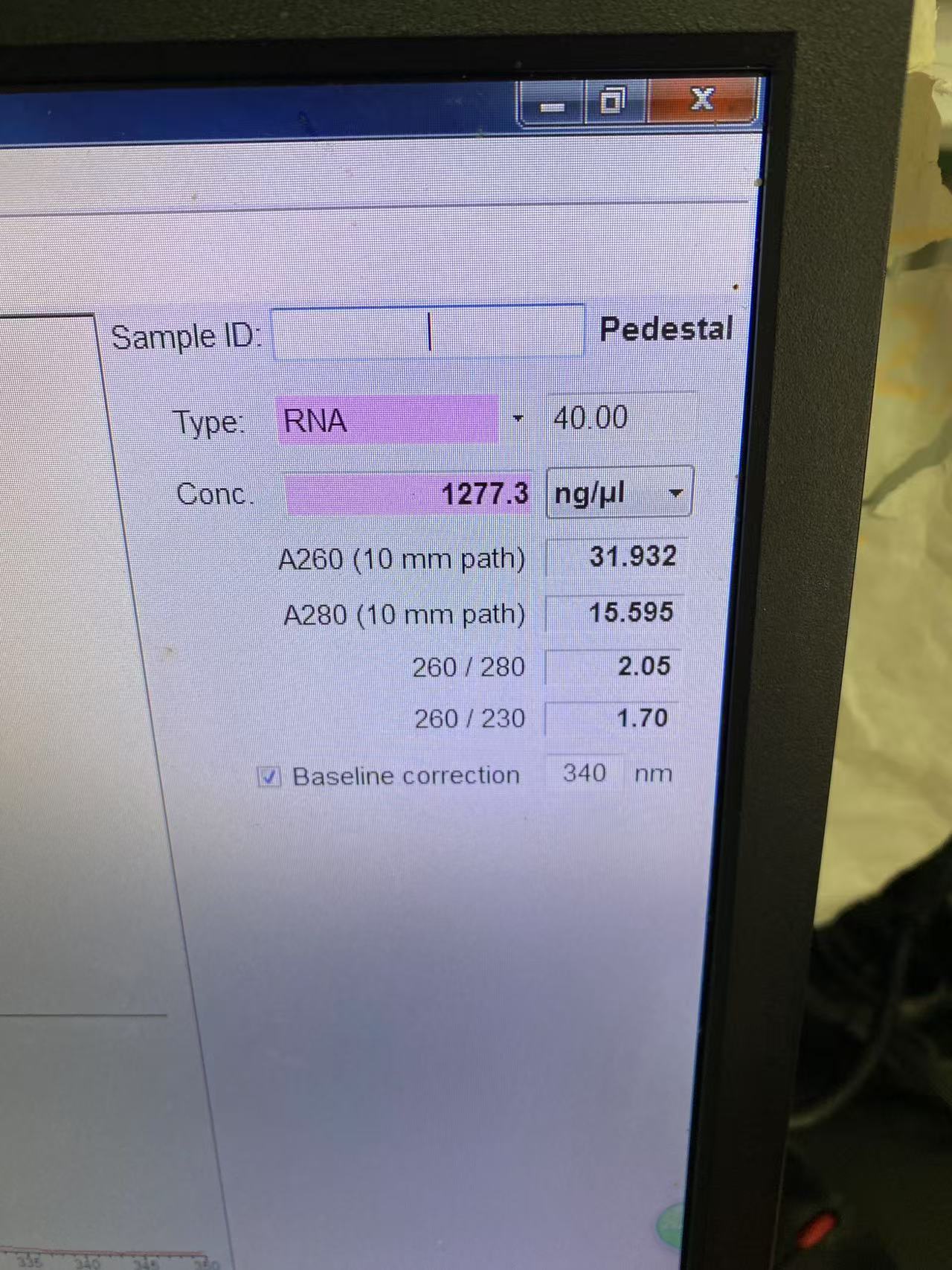


**P5:**


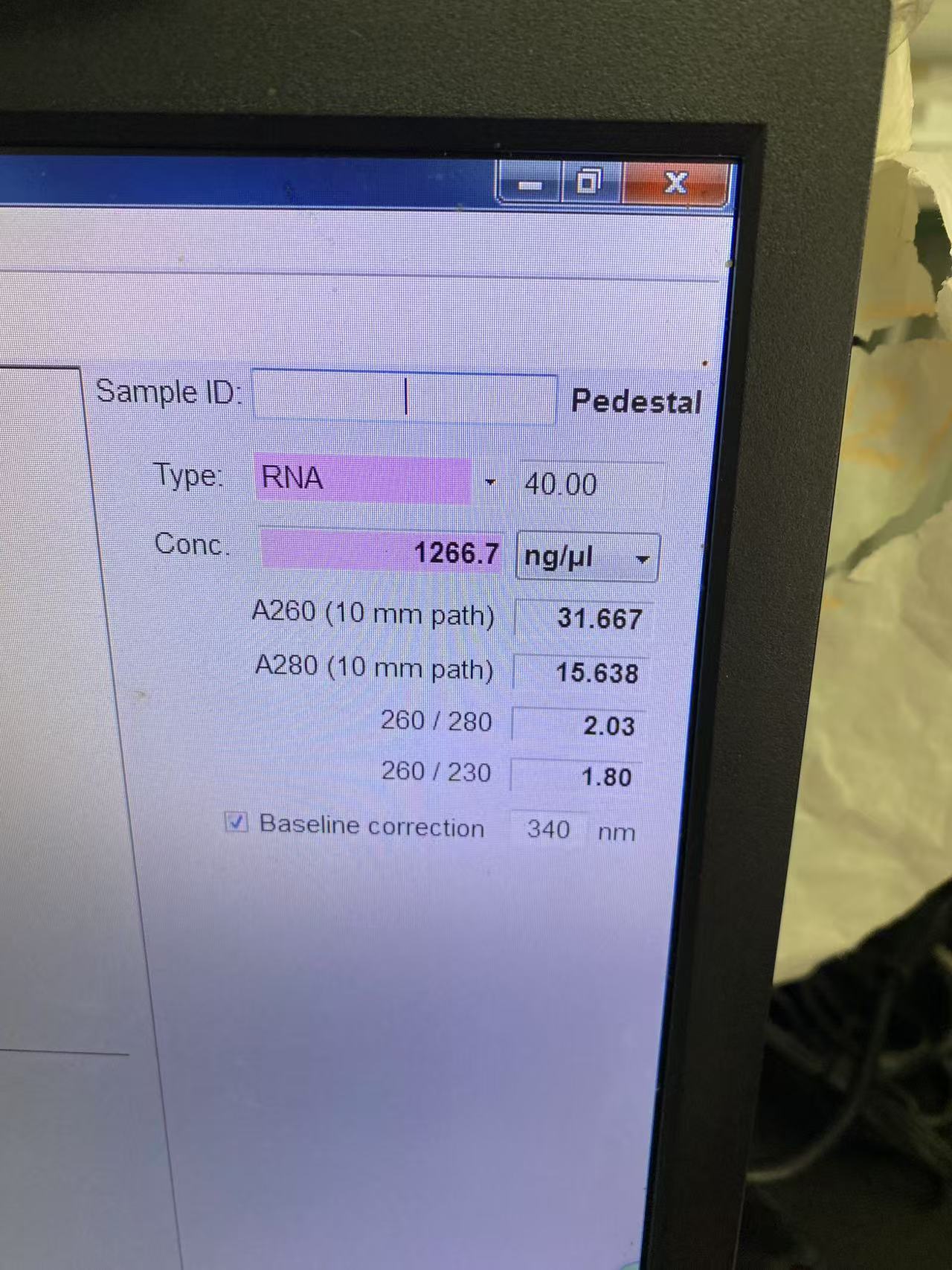


**T6:**


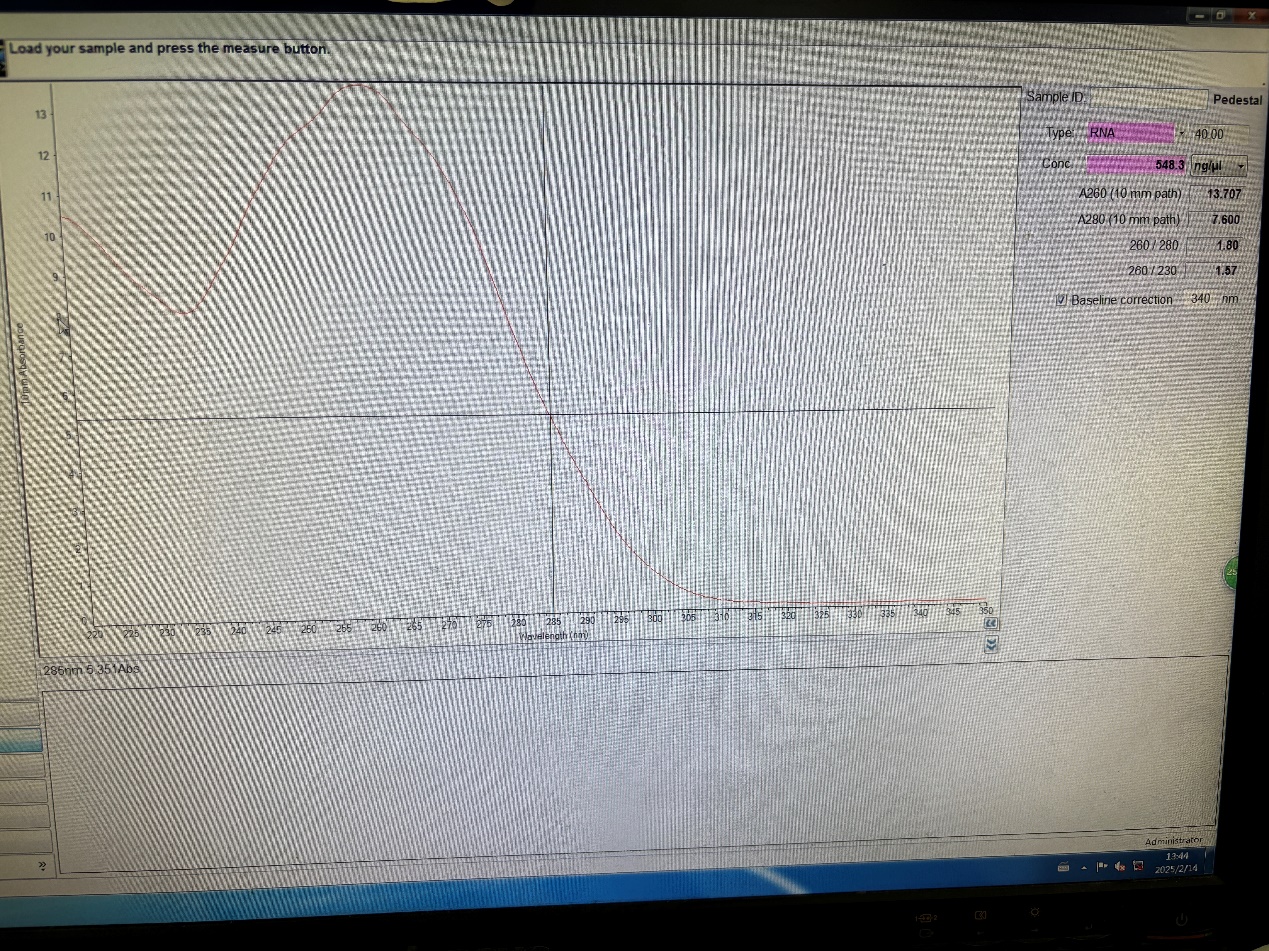


**P6:**


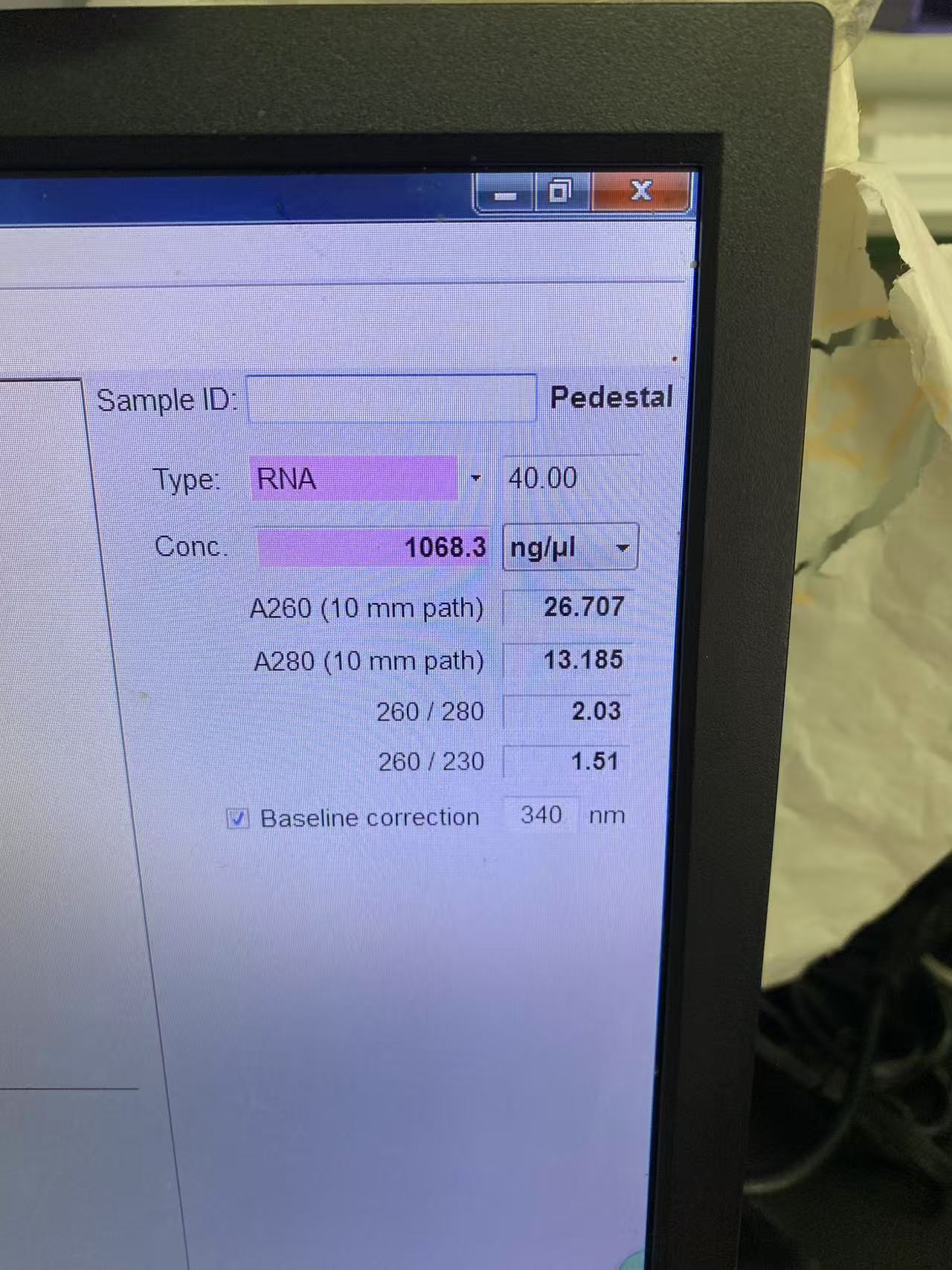


**T7:**


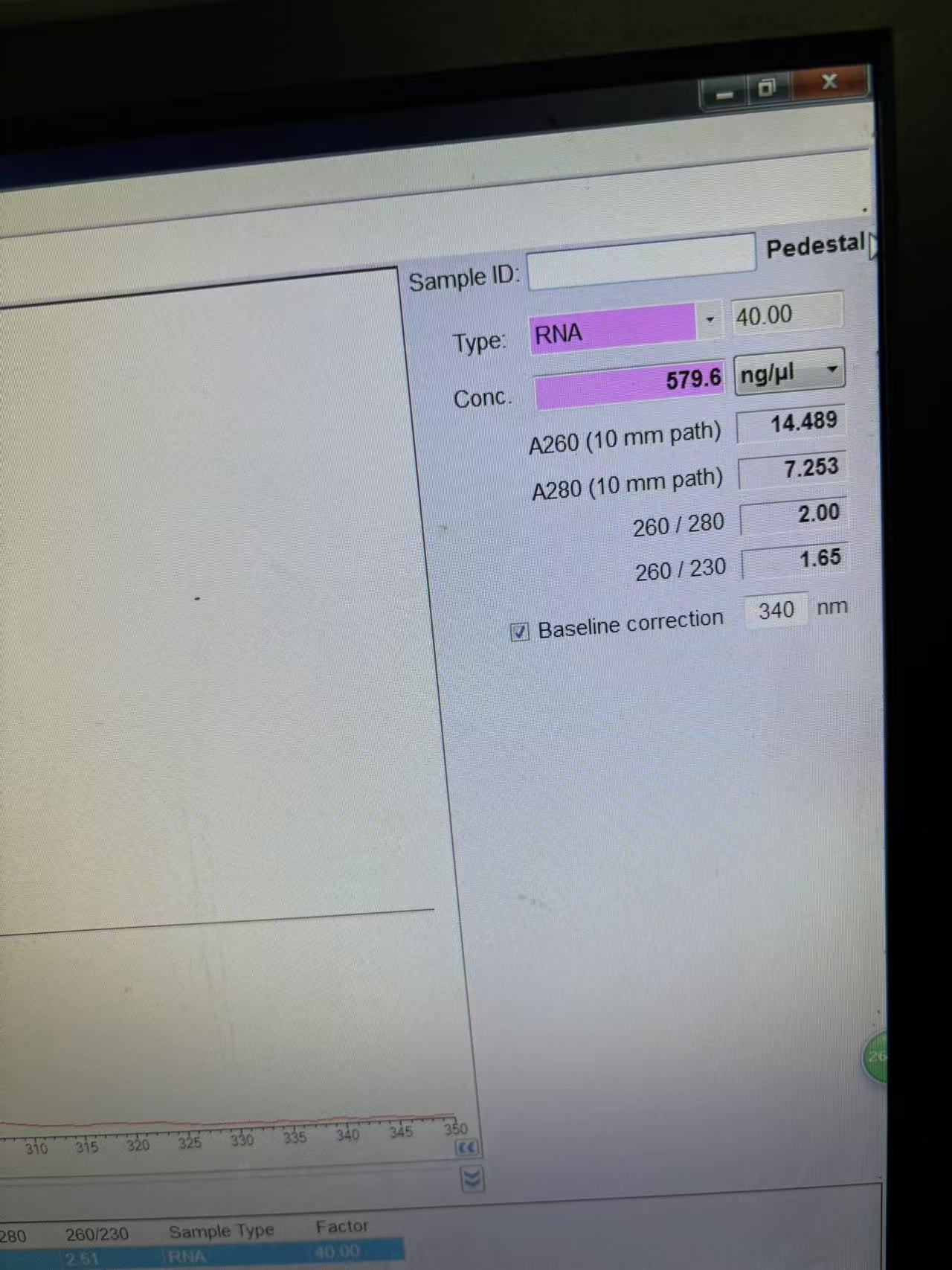


**P7:**


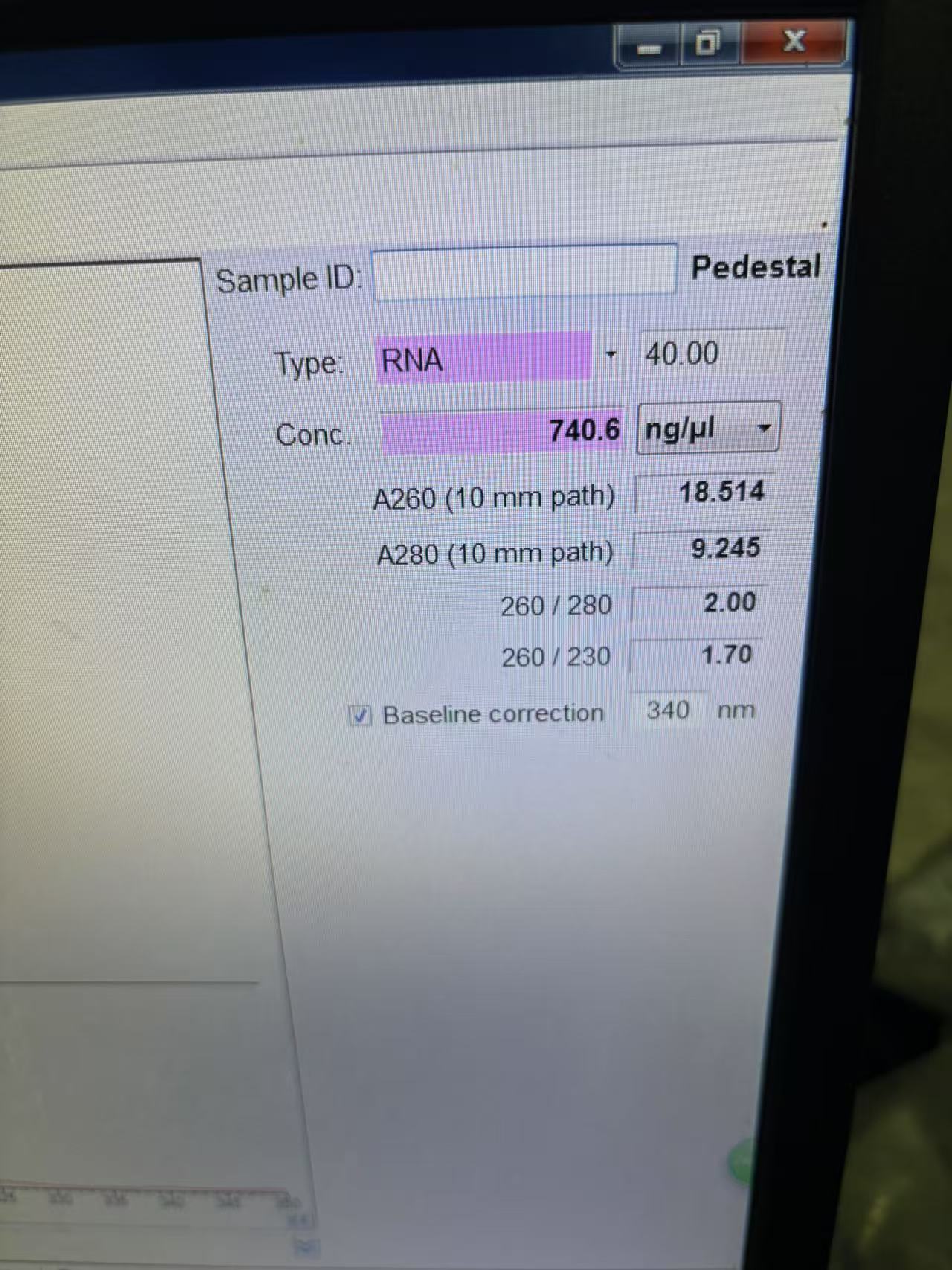


**T8:**

**
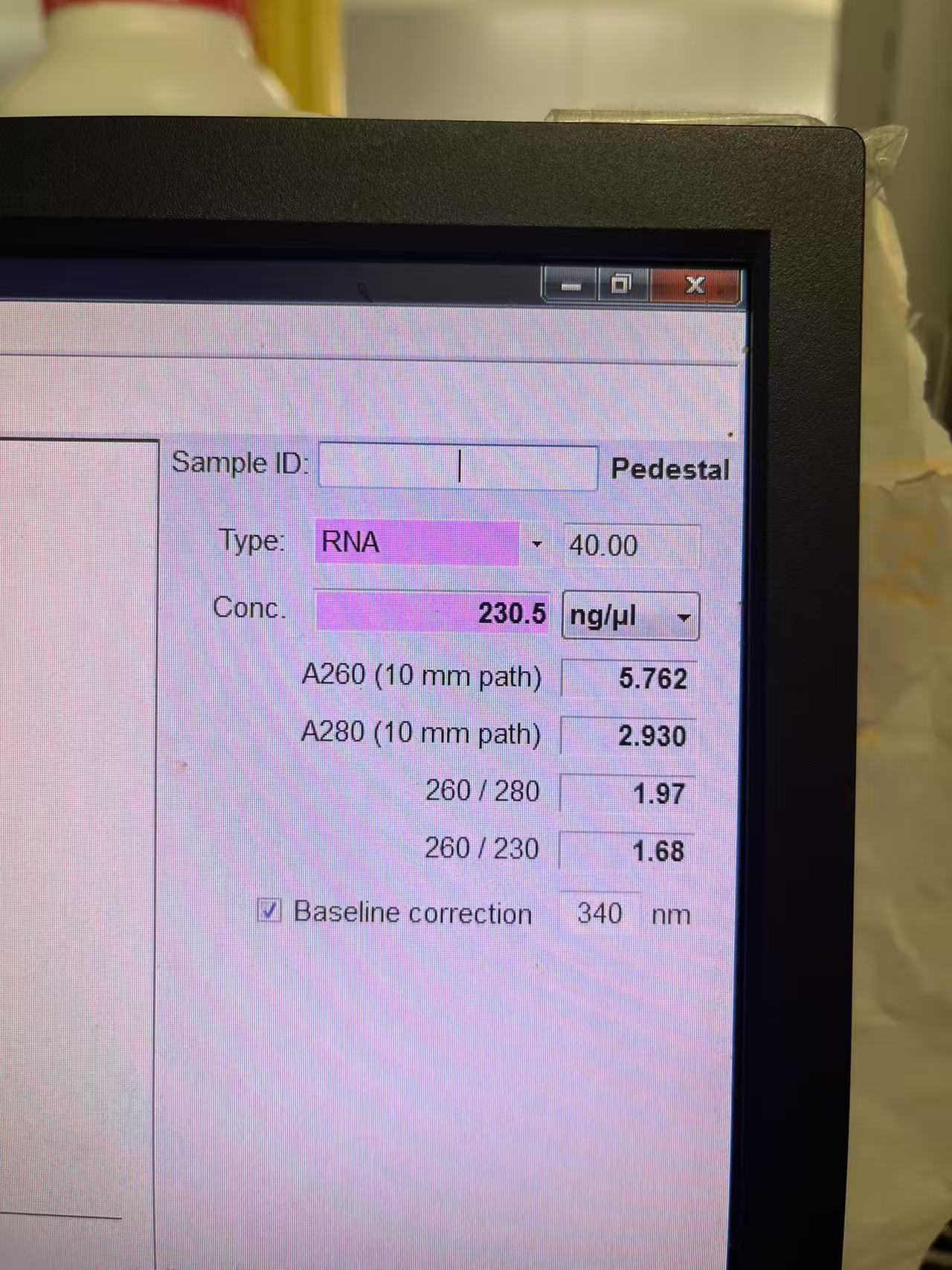
**

**P8:**

**
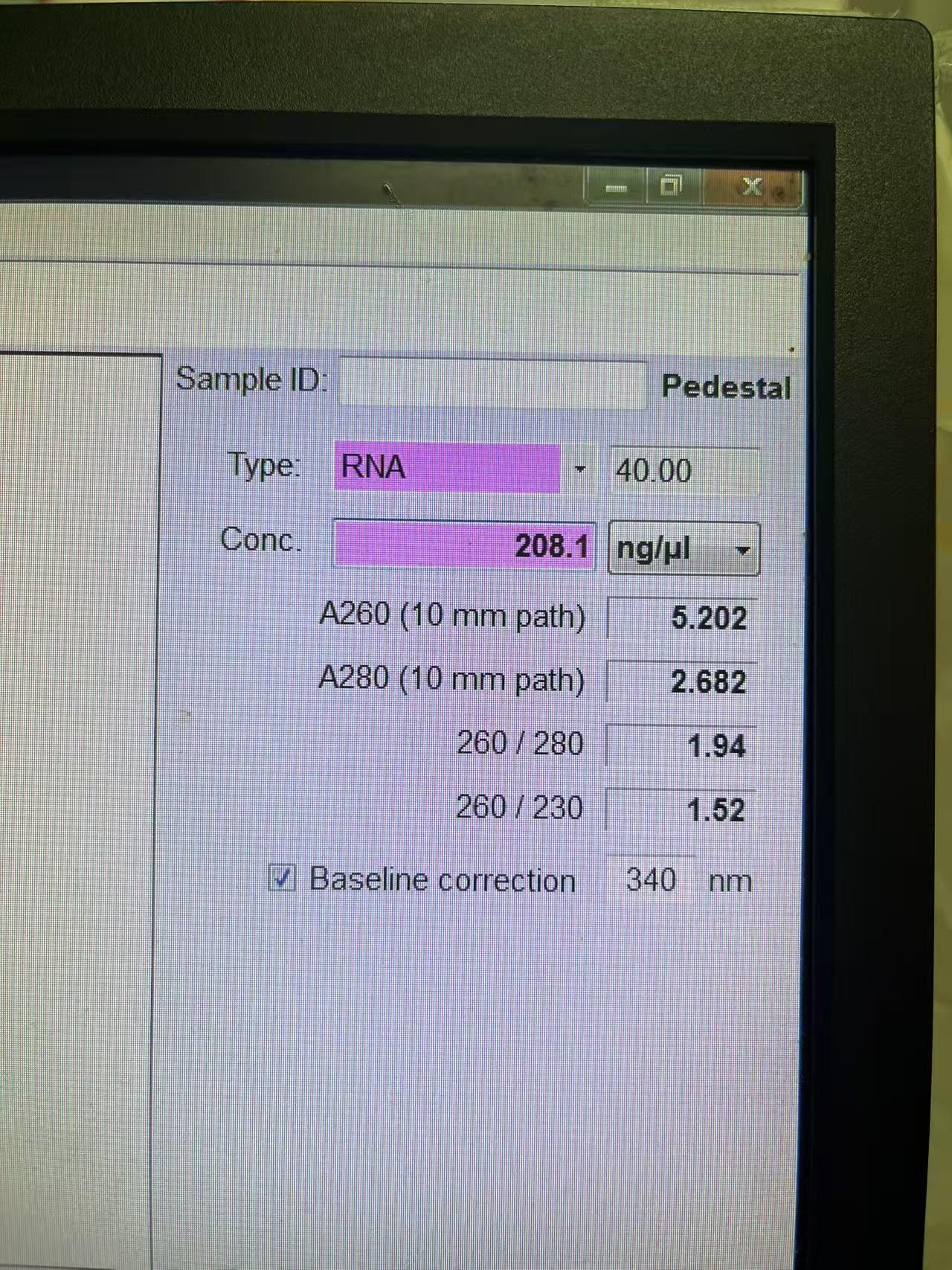
**

**T9:**

**
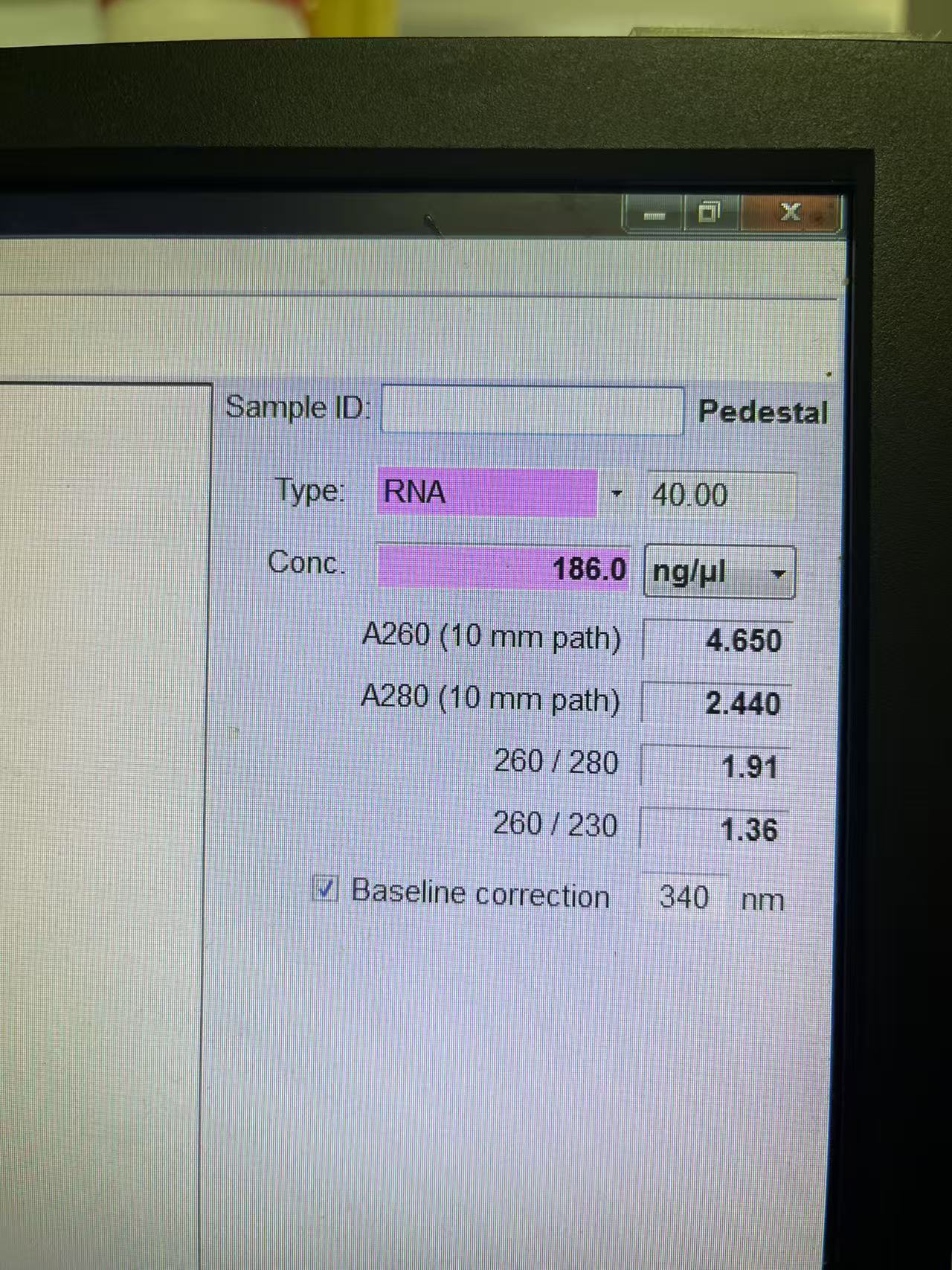
**

**P9:**

**
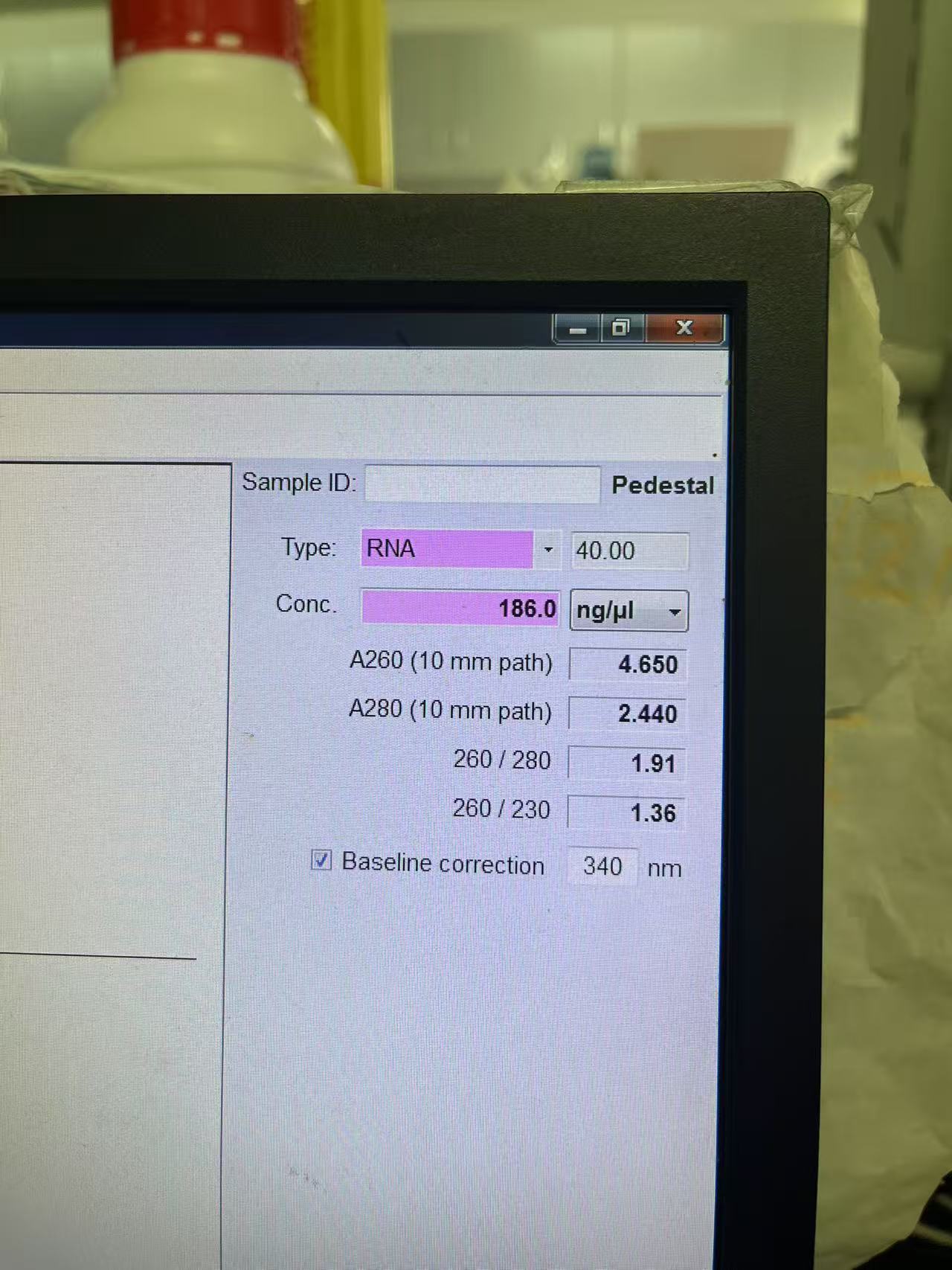
**

**T10:**

**
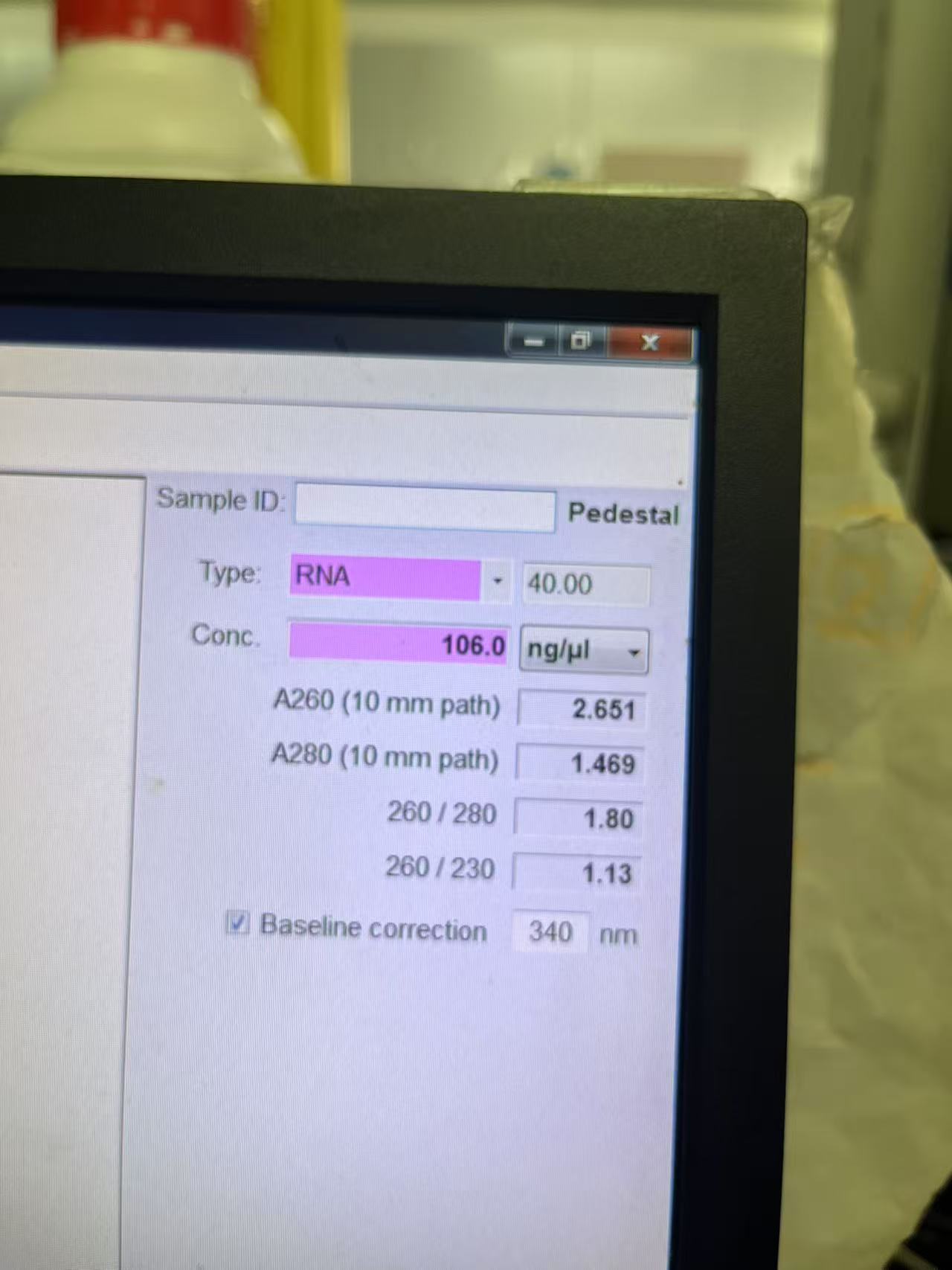
**

**P10:**

**
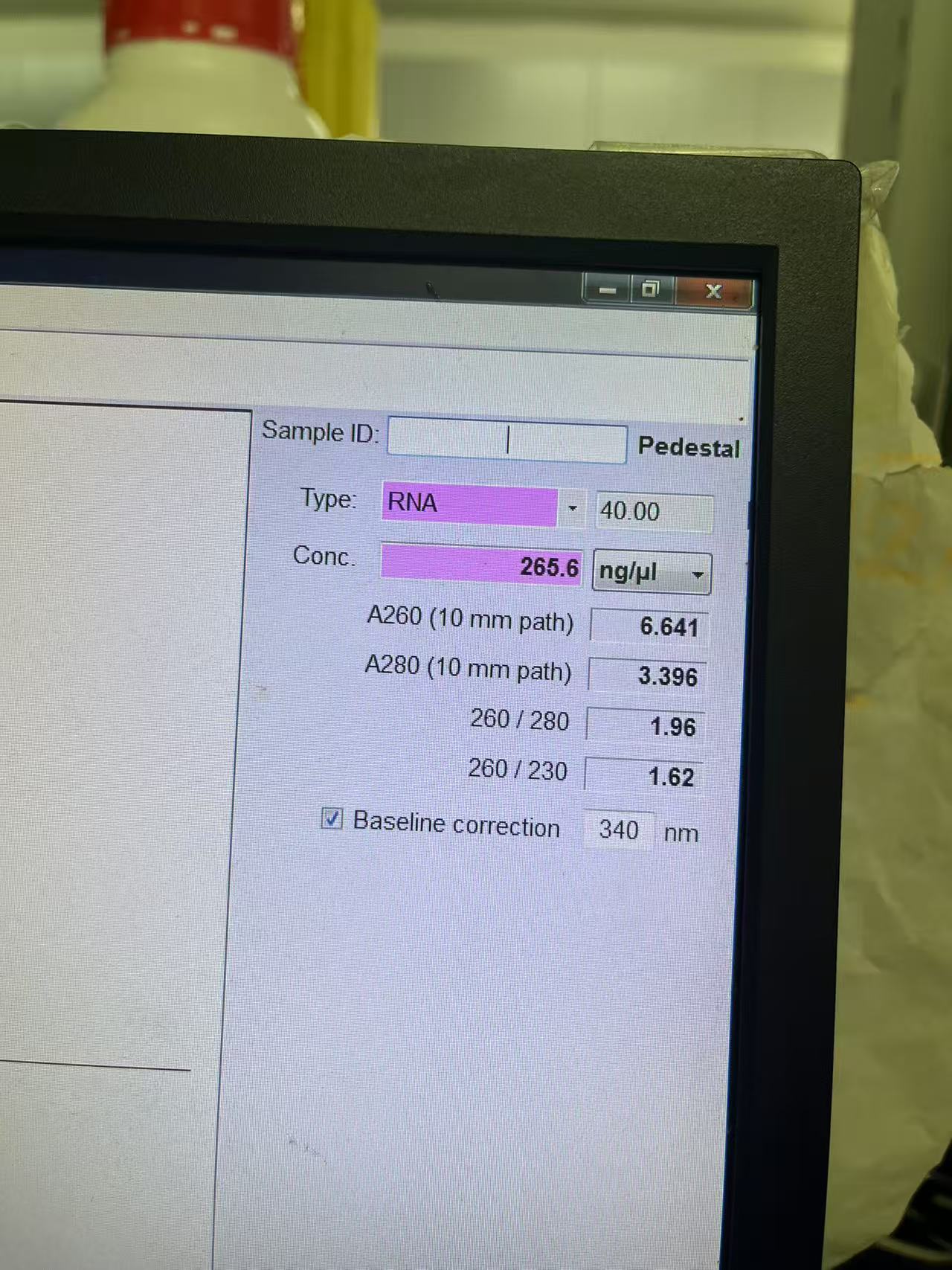
**

**T11:**

**
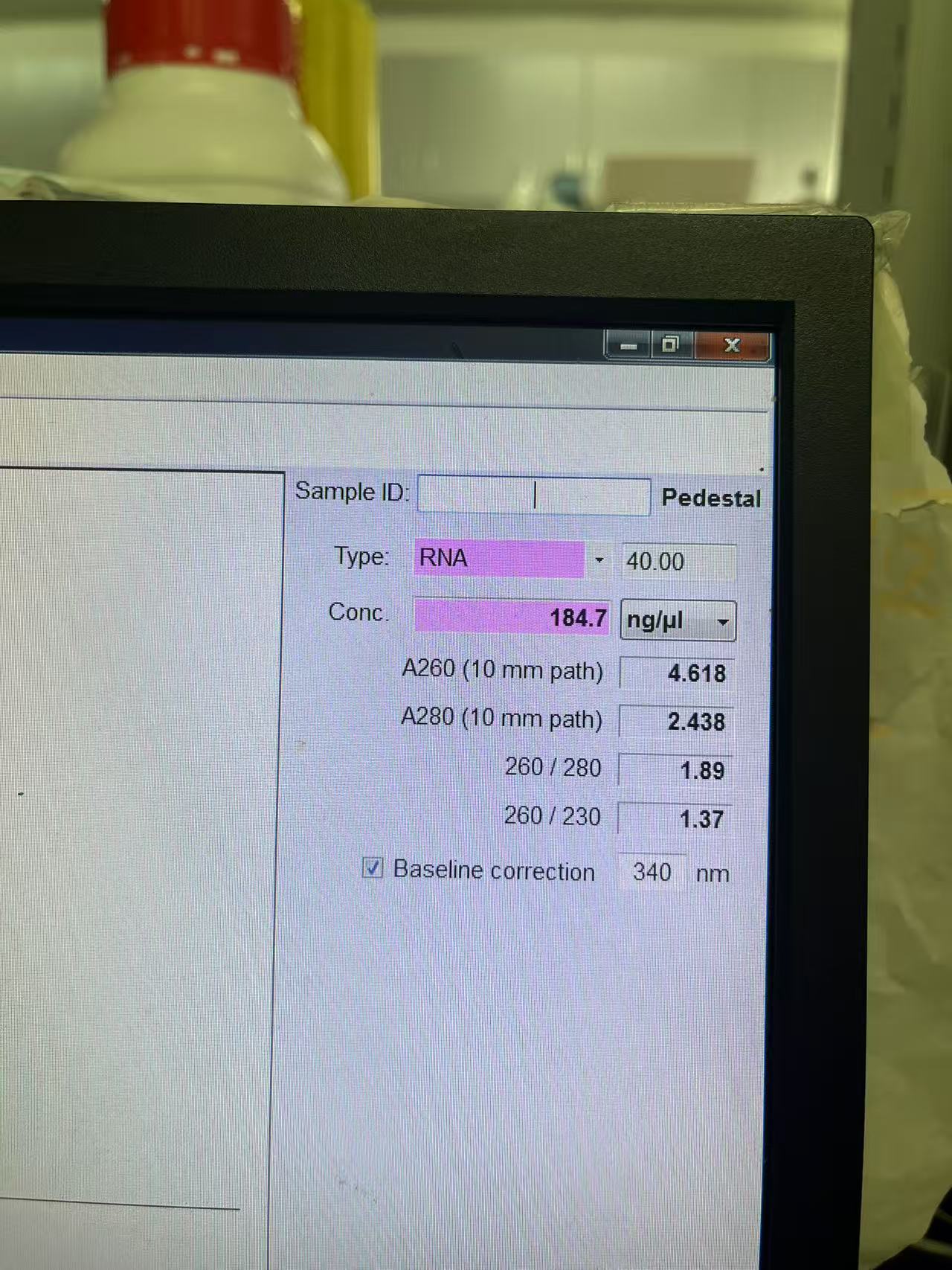
**

**P11:**

**
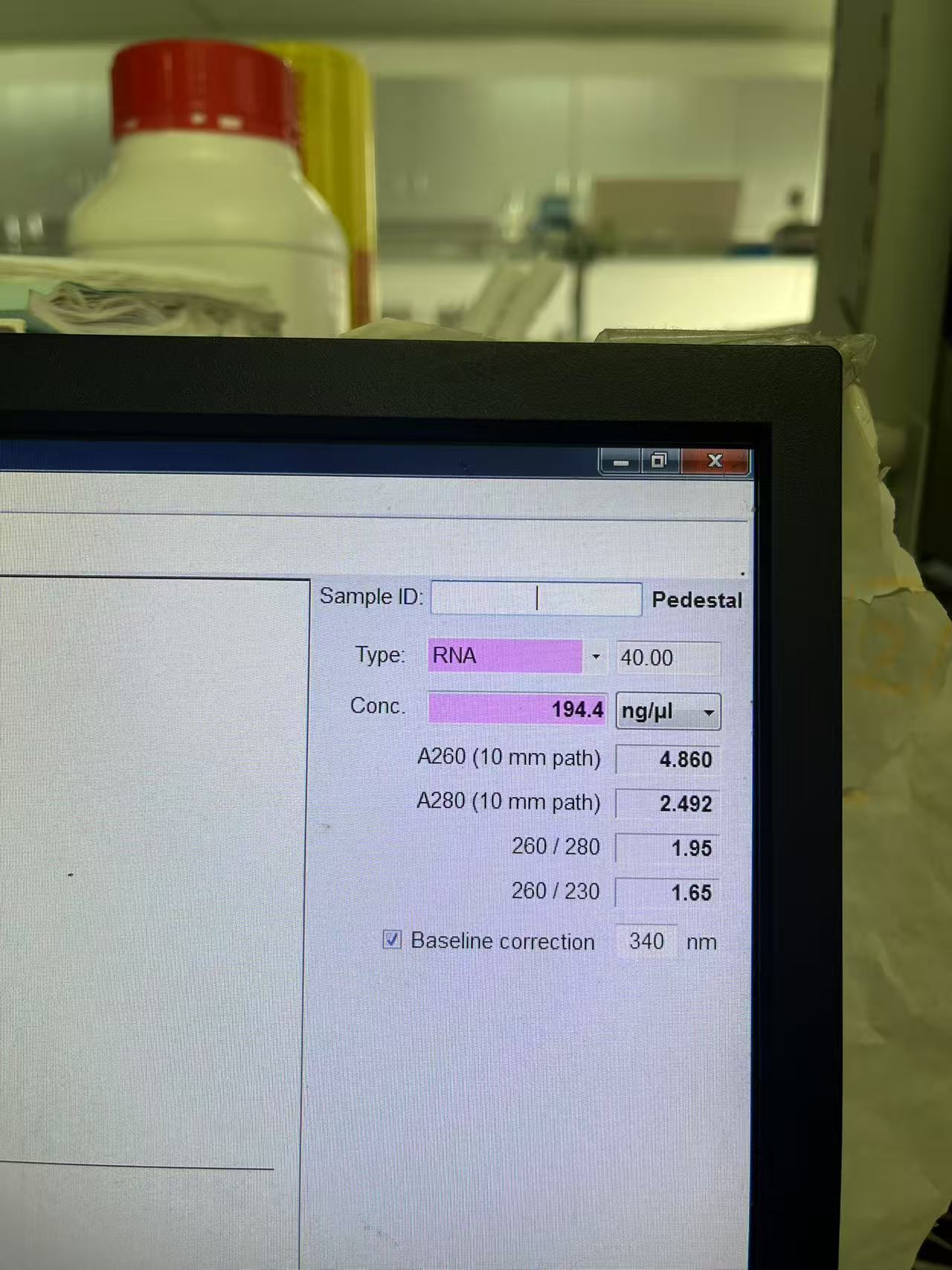
**

**T12:**

**
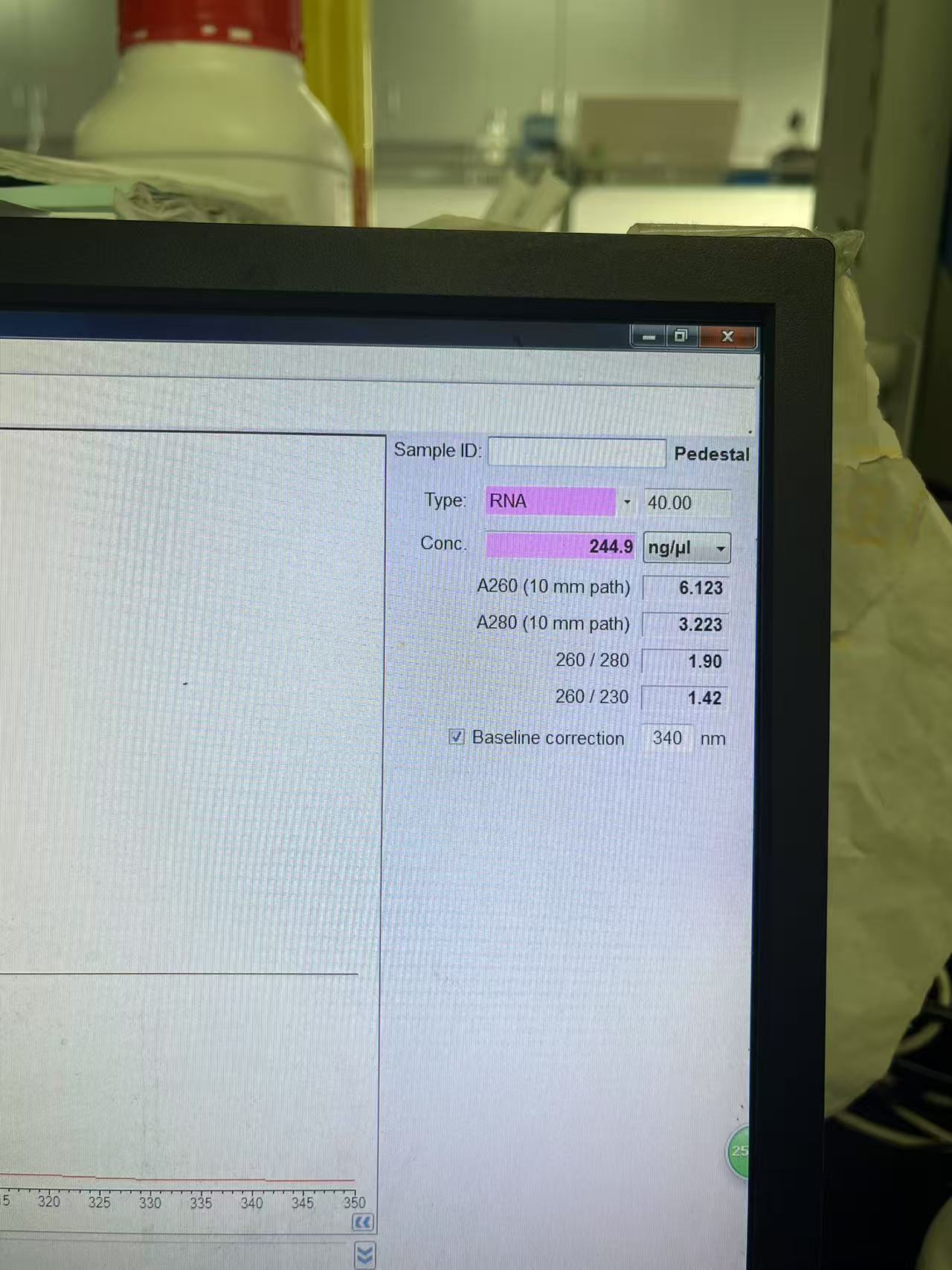
**

**P12:**

**
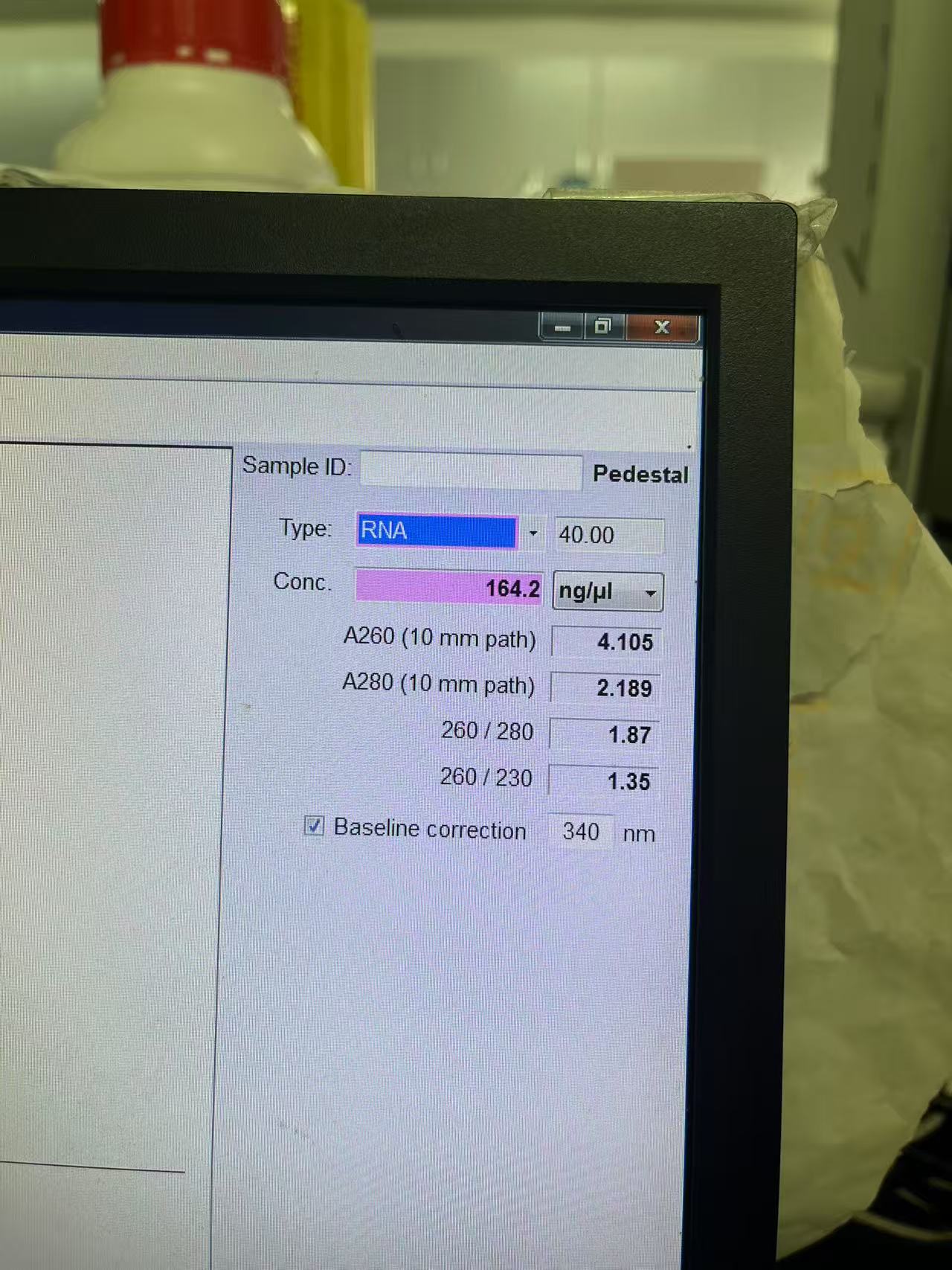
**

**Figure 5B:**

**KYSE30-EV:**


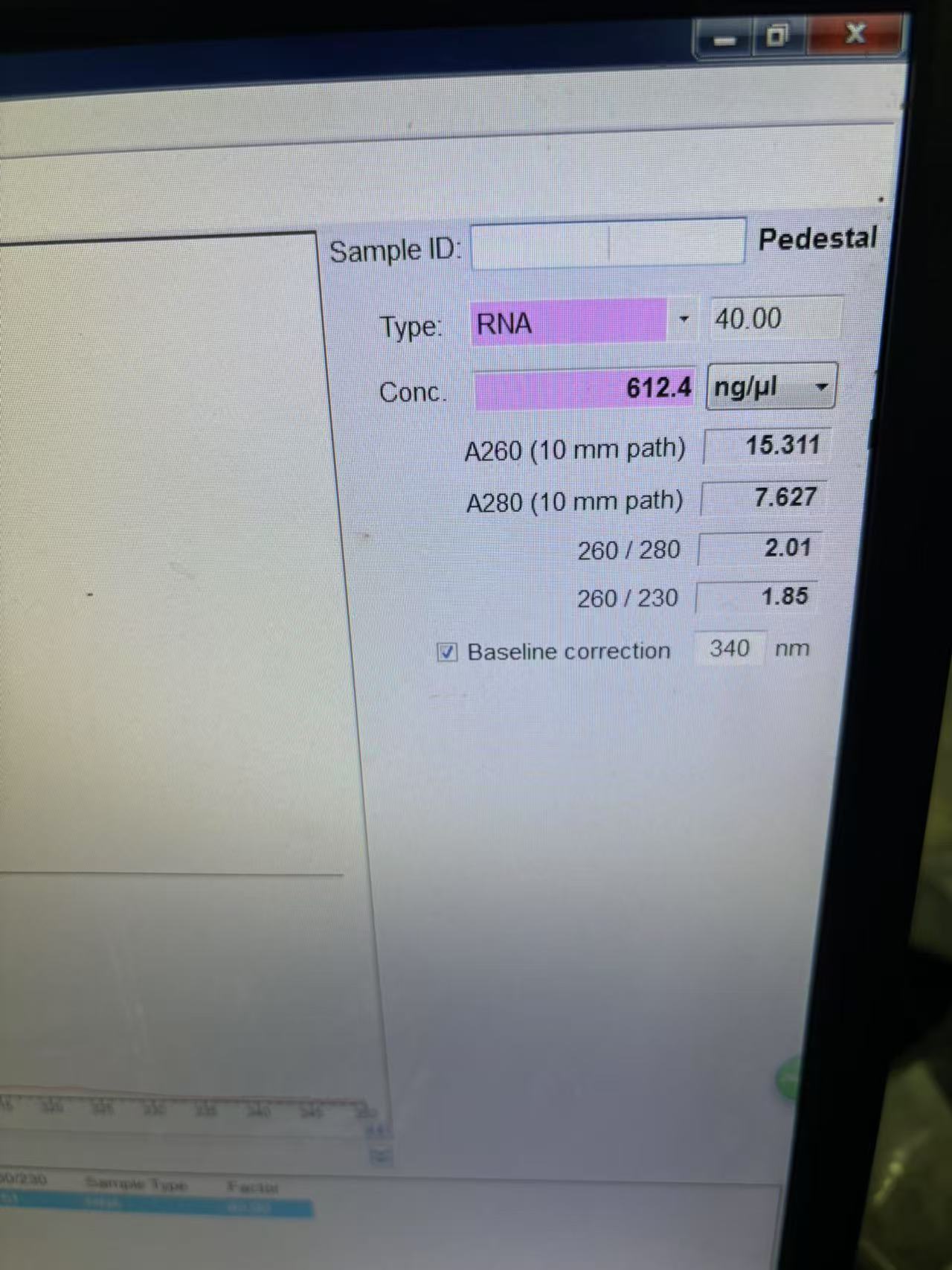


**KYSE30-EV+miR-203a-3p:**


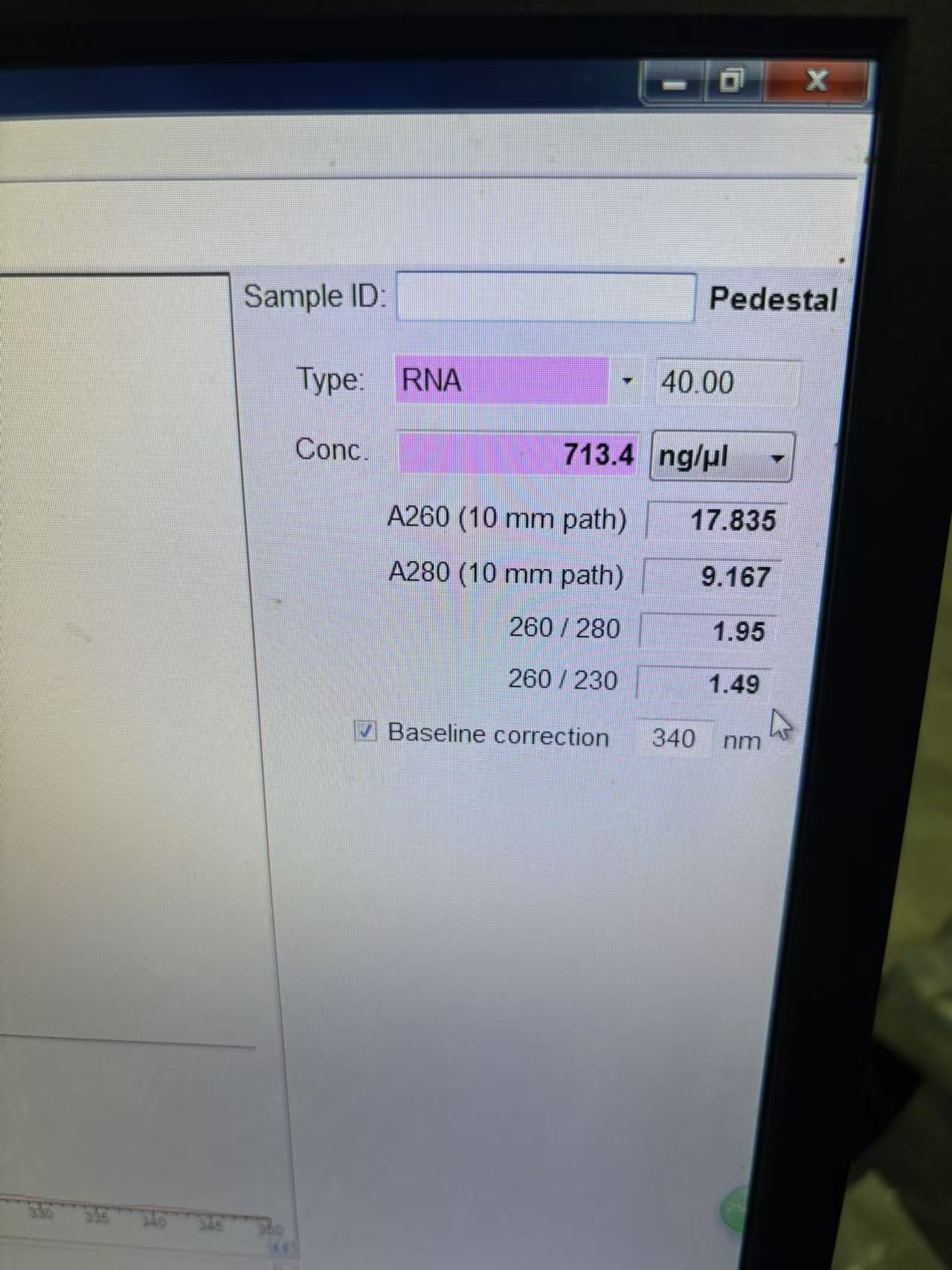


**Drp1:**

**
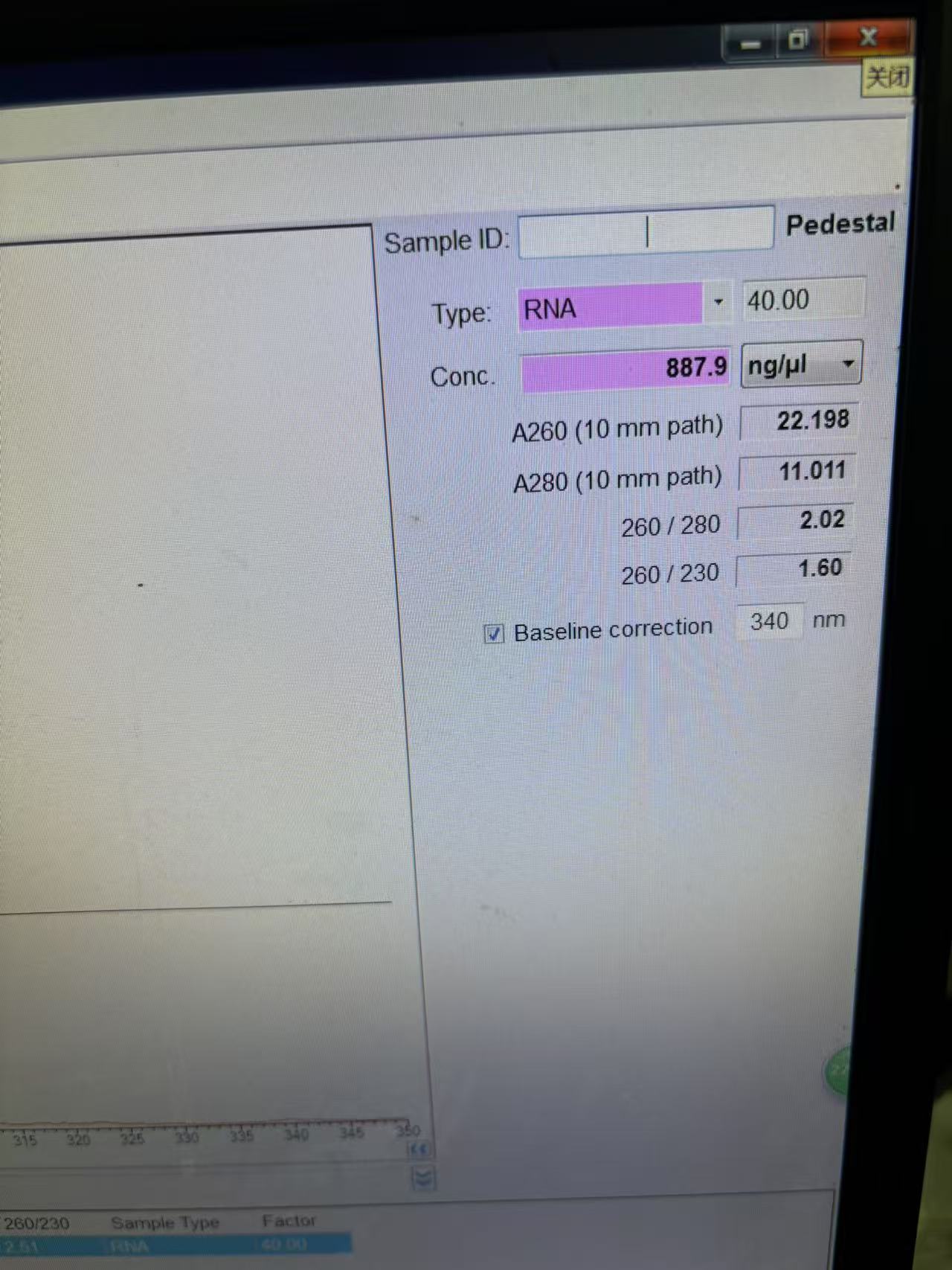
**

**Drp1+miR-203a-3p:**

**
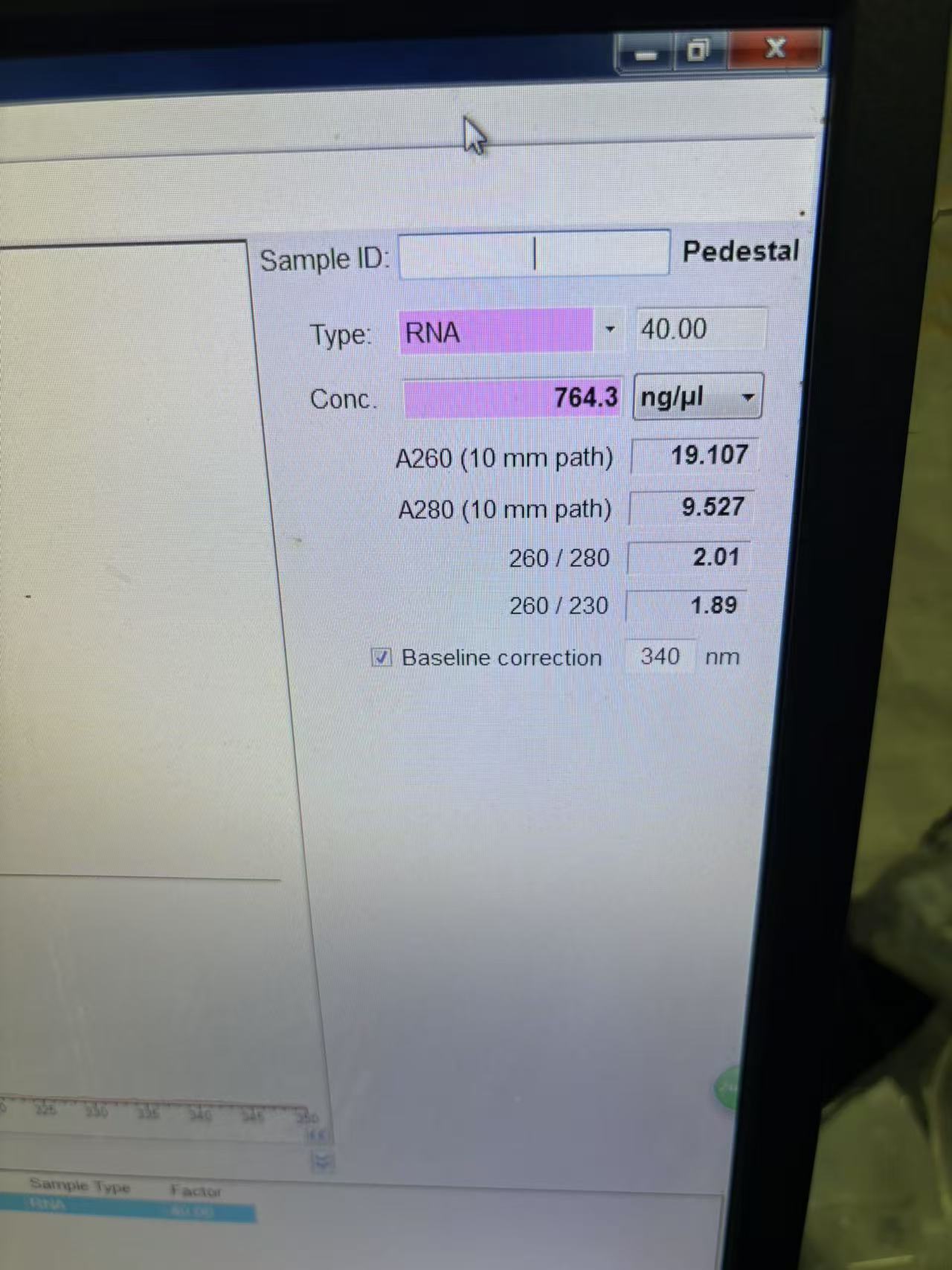
**

**EC9706-EV:**

**
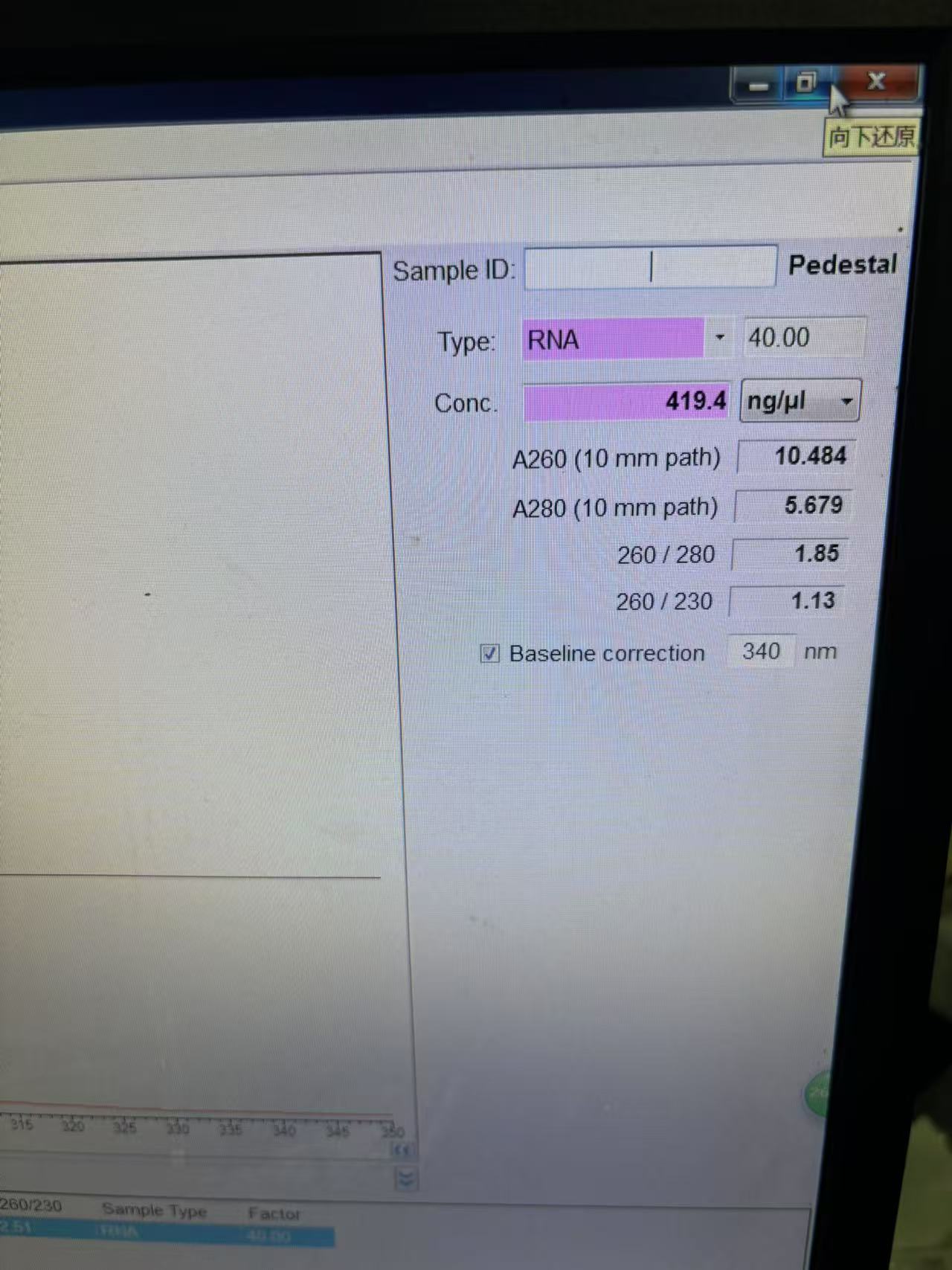
**

**EC9706-EV+miR-203a-3p:**

**
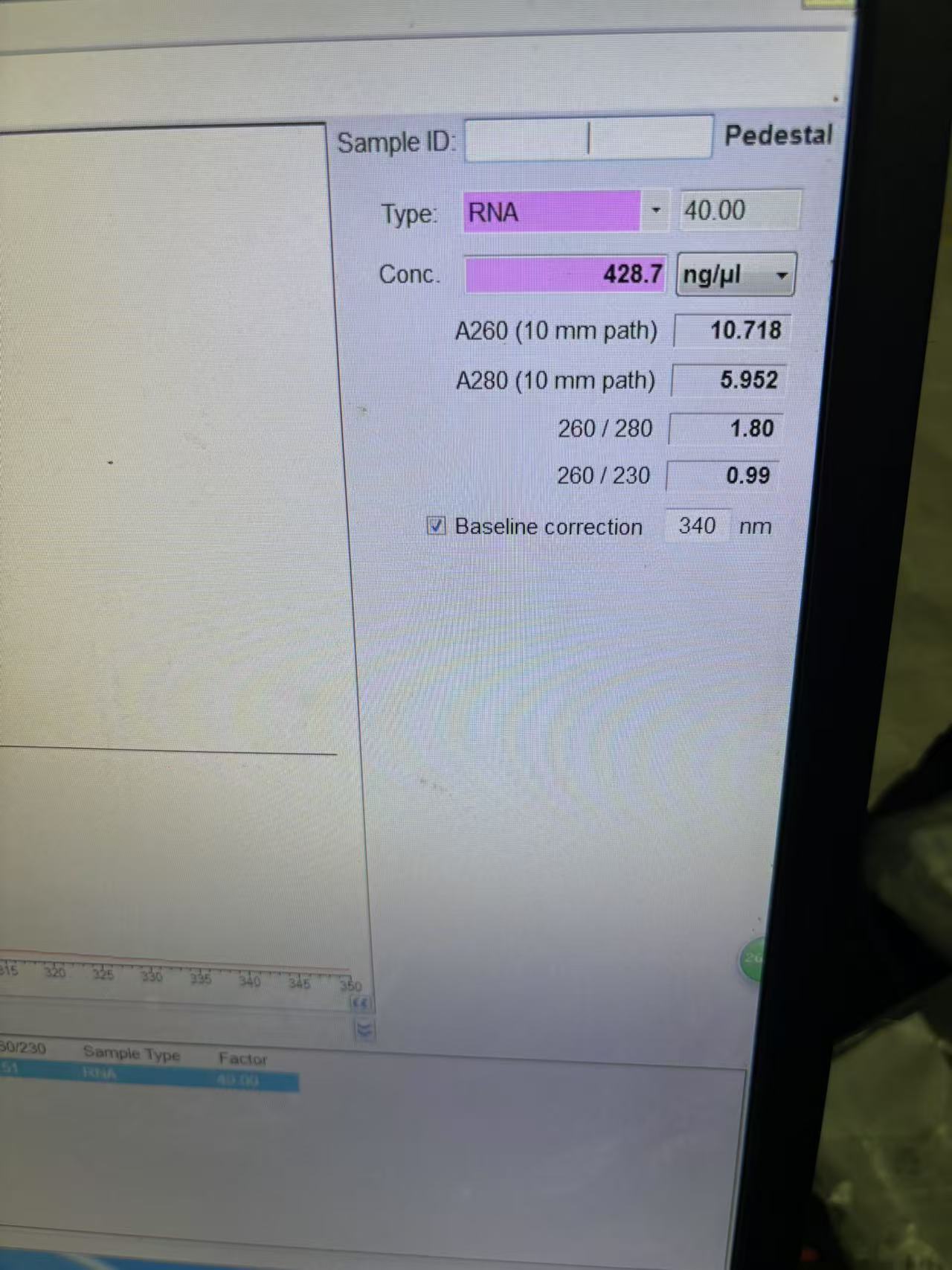
**

**EC9706-Drp1:**

**
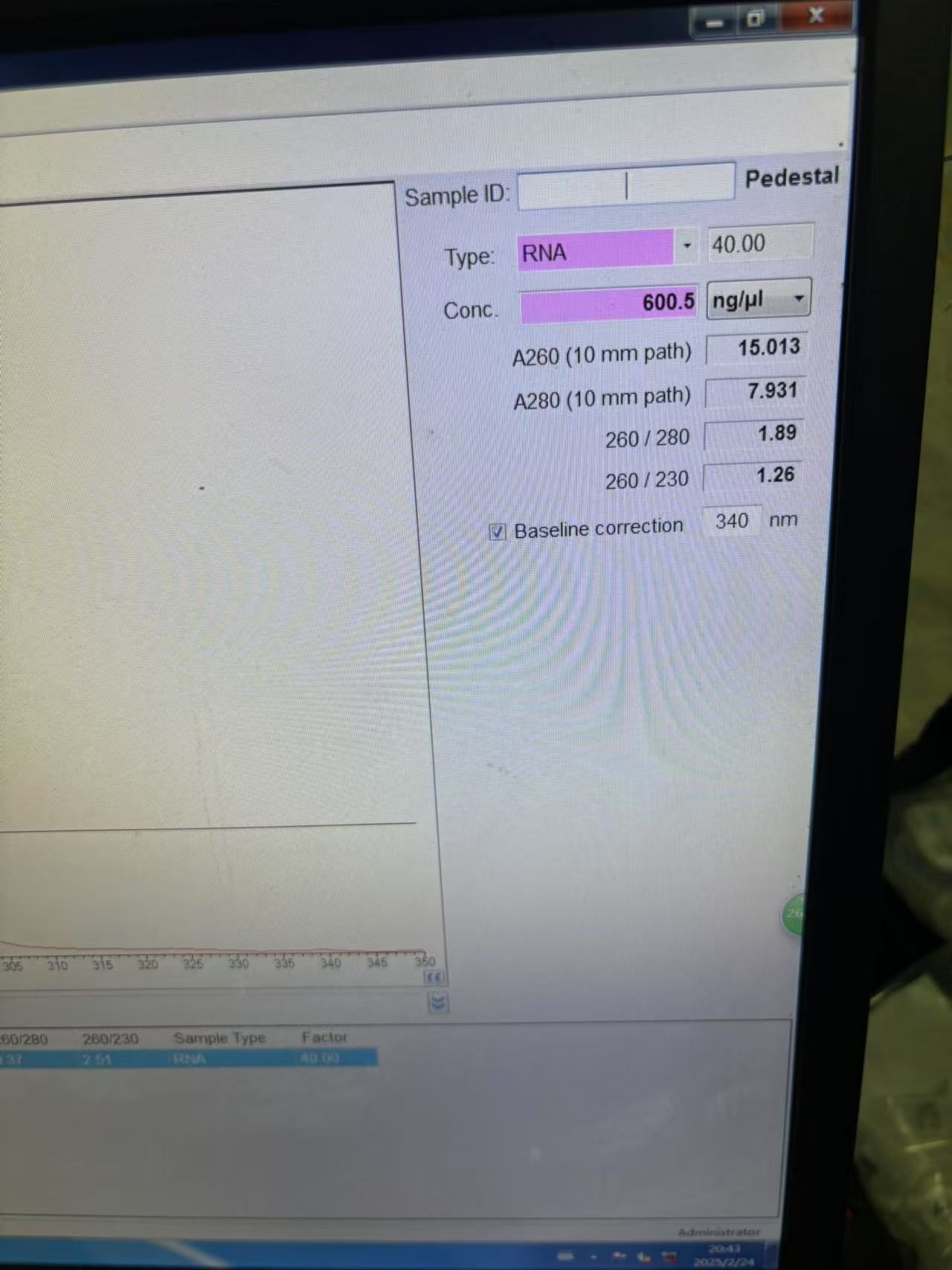
**

**EC9706-Drp1+miR-203a-3p:**

**
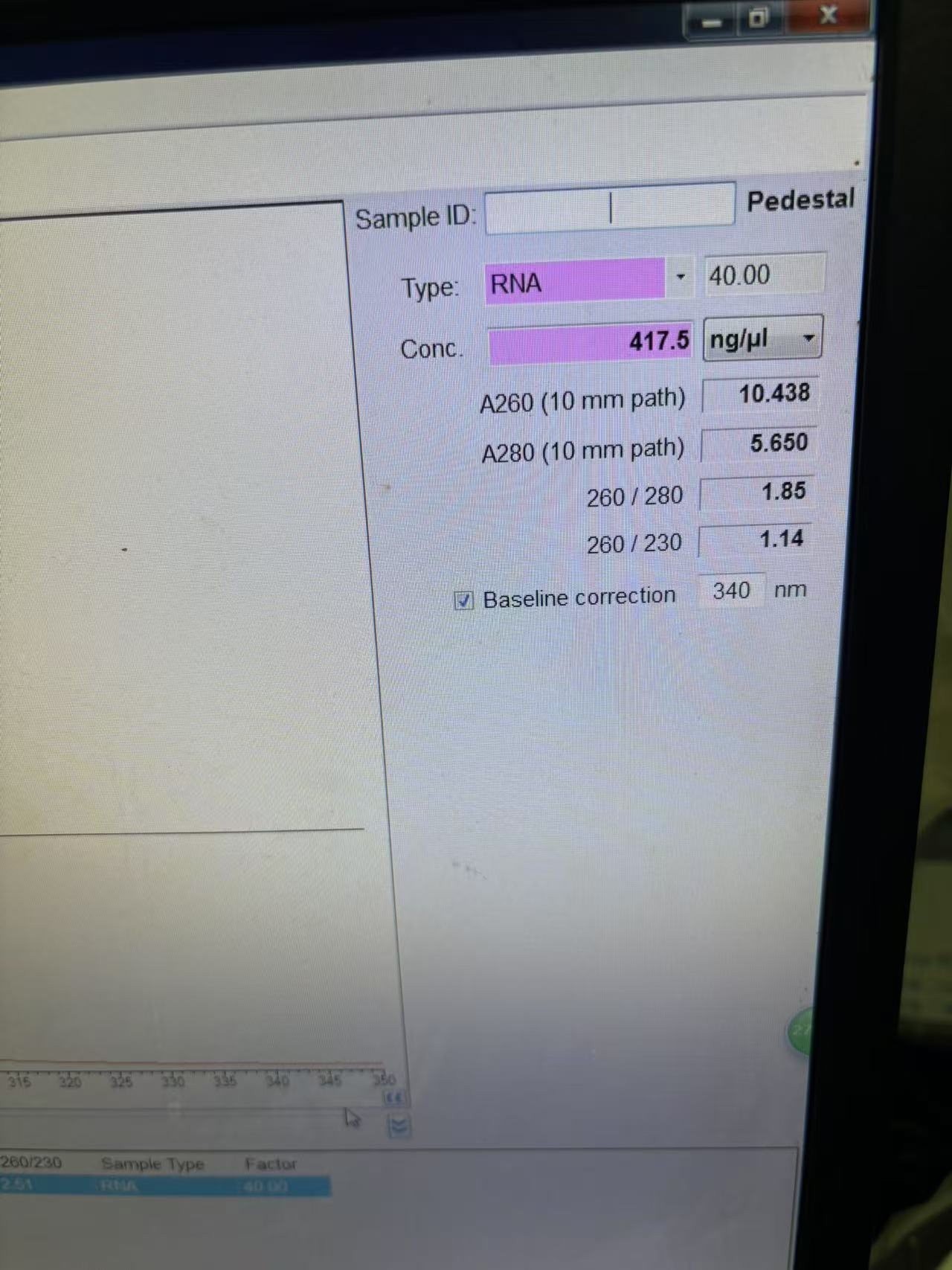
**

**Figure 5C:**

**KYSE30-EV:**

**
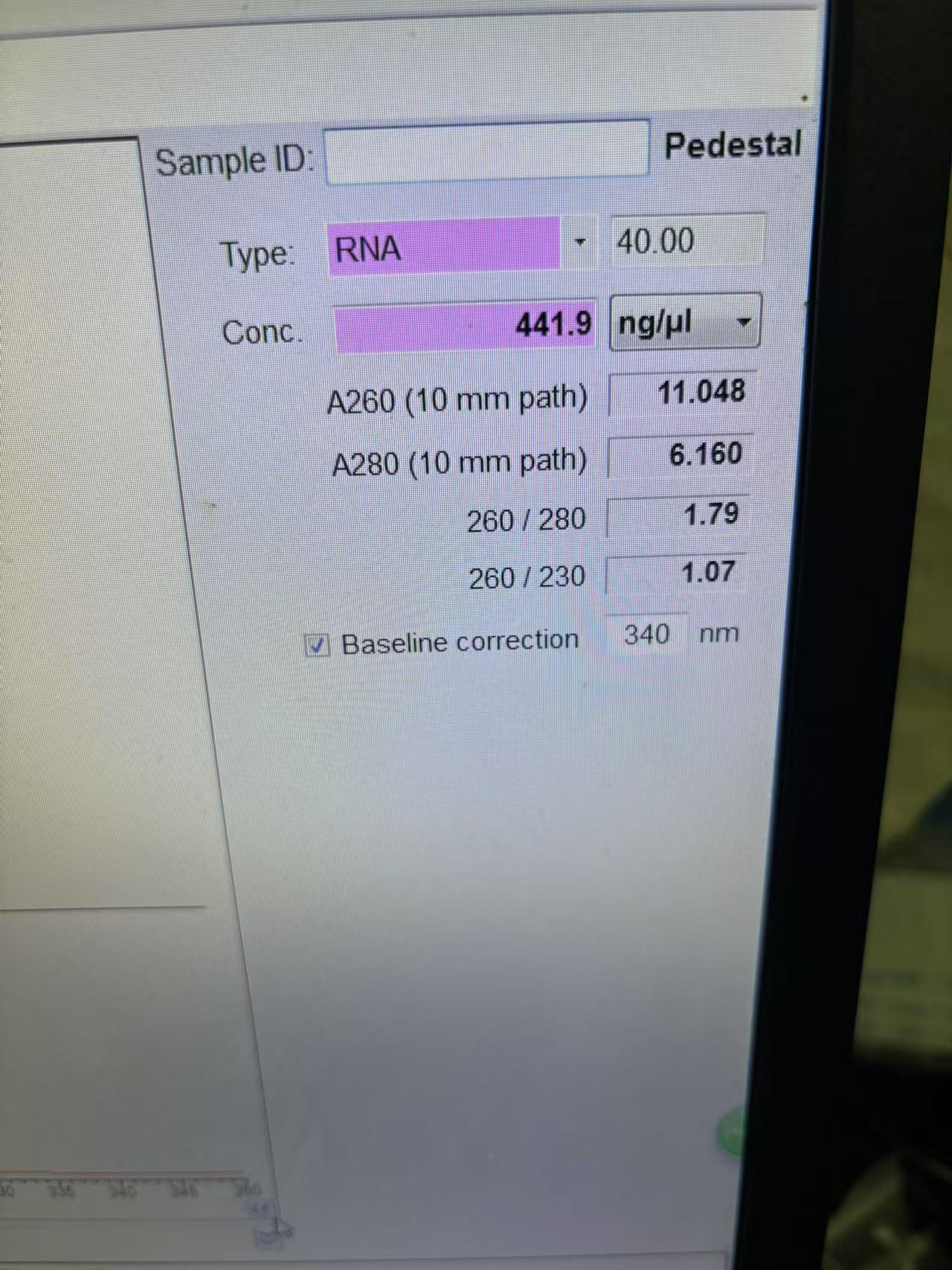
**

**KYSE30-EV+miR-203a-3p:**

**
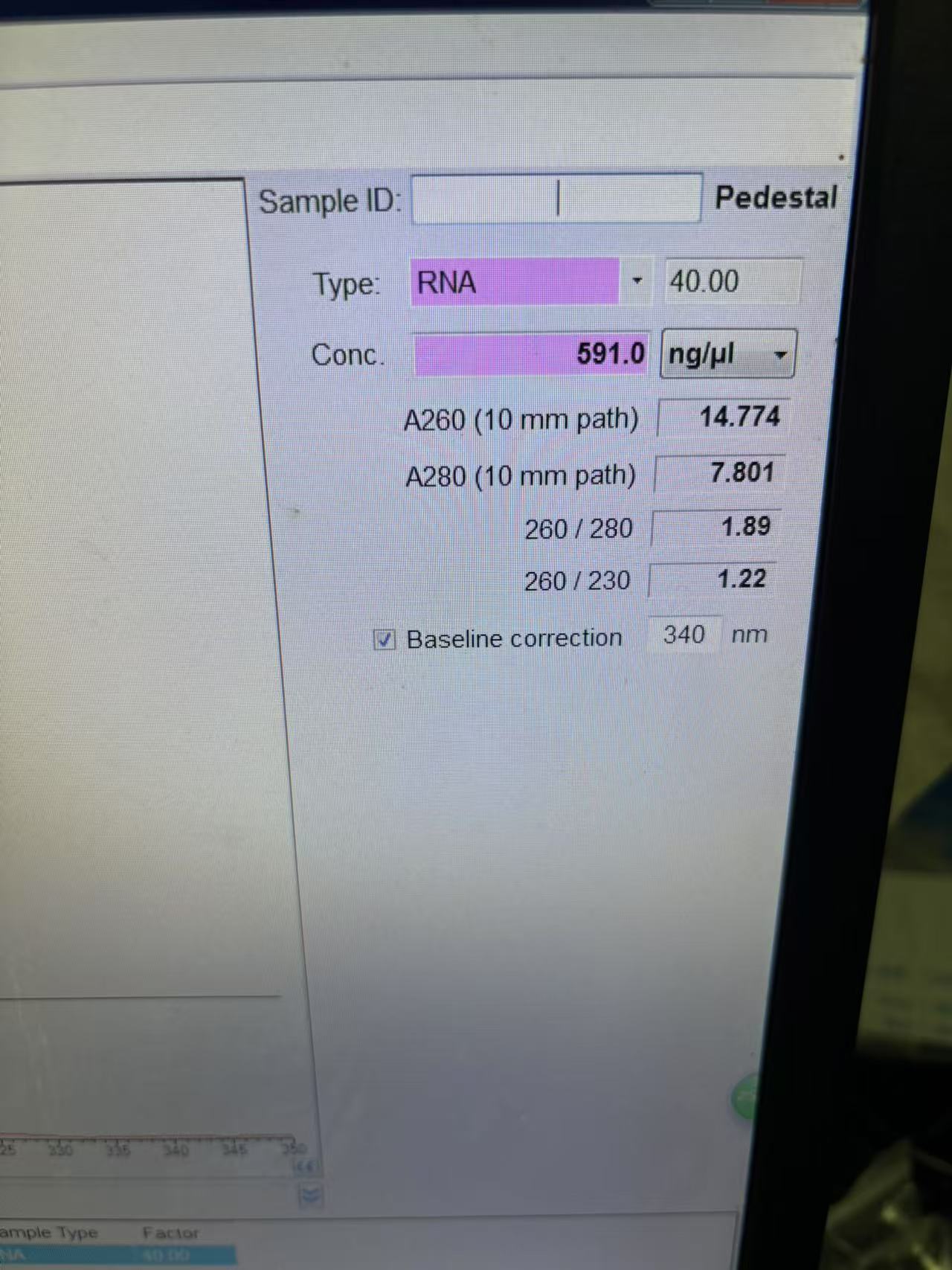
**

**KYSE30-Drp1:**

**
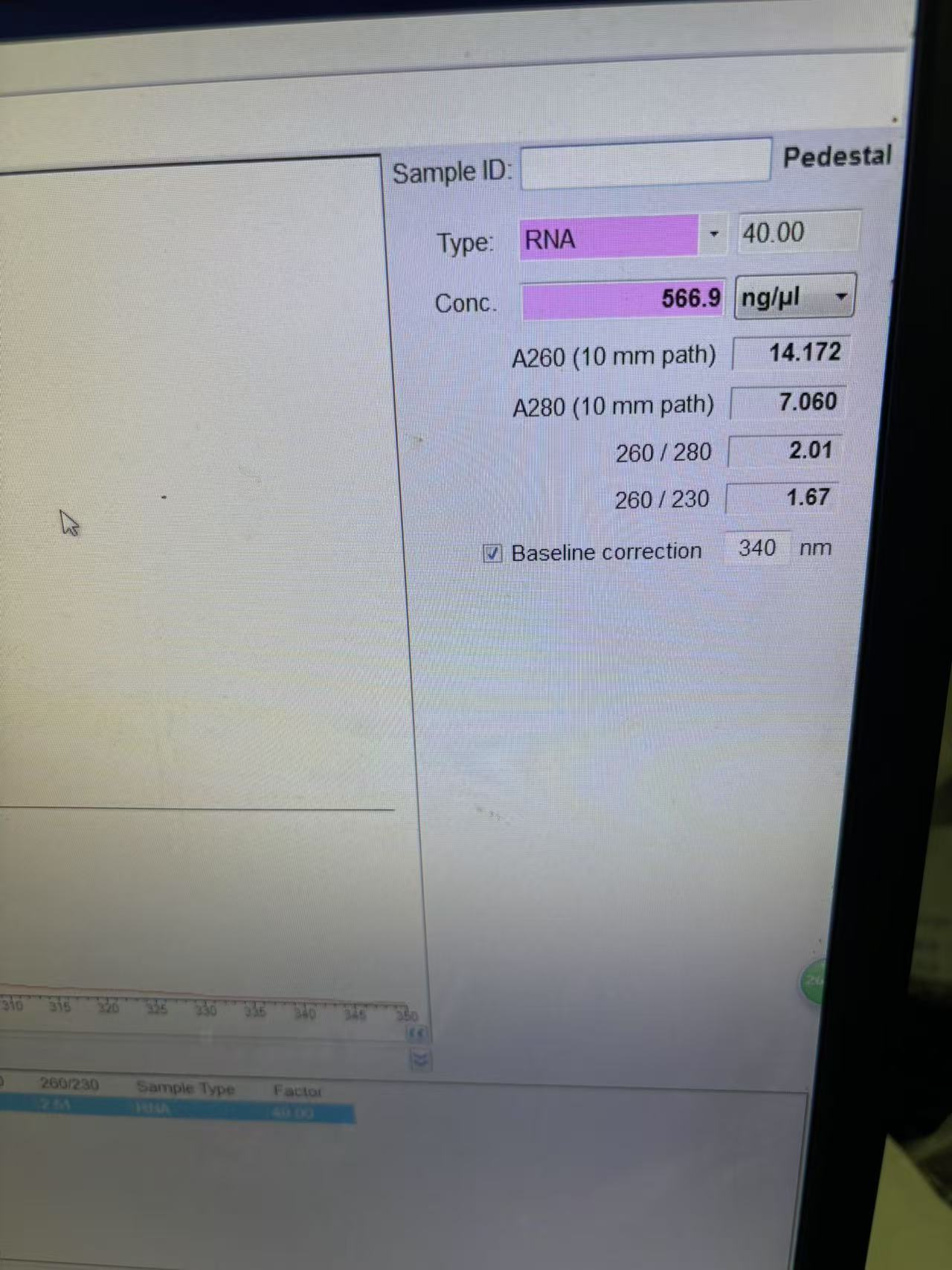
**

**KYSE30-Drp1+miR-203a-3p:**

**
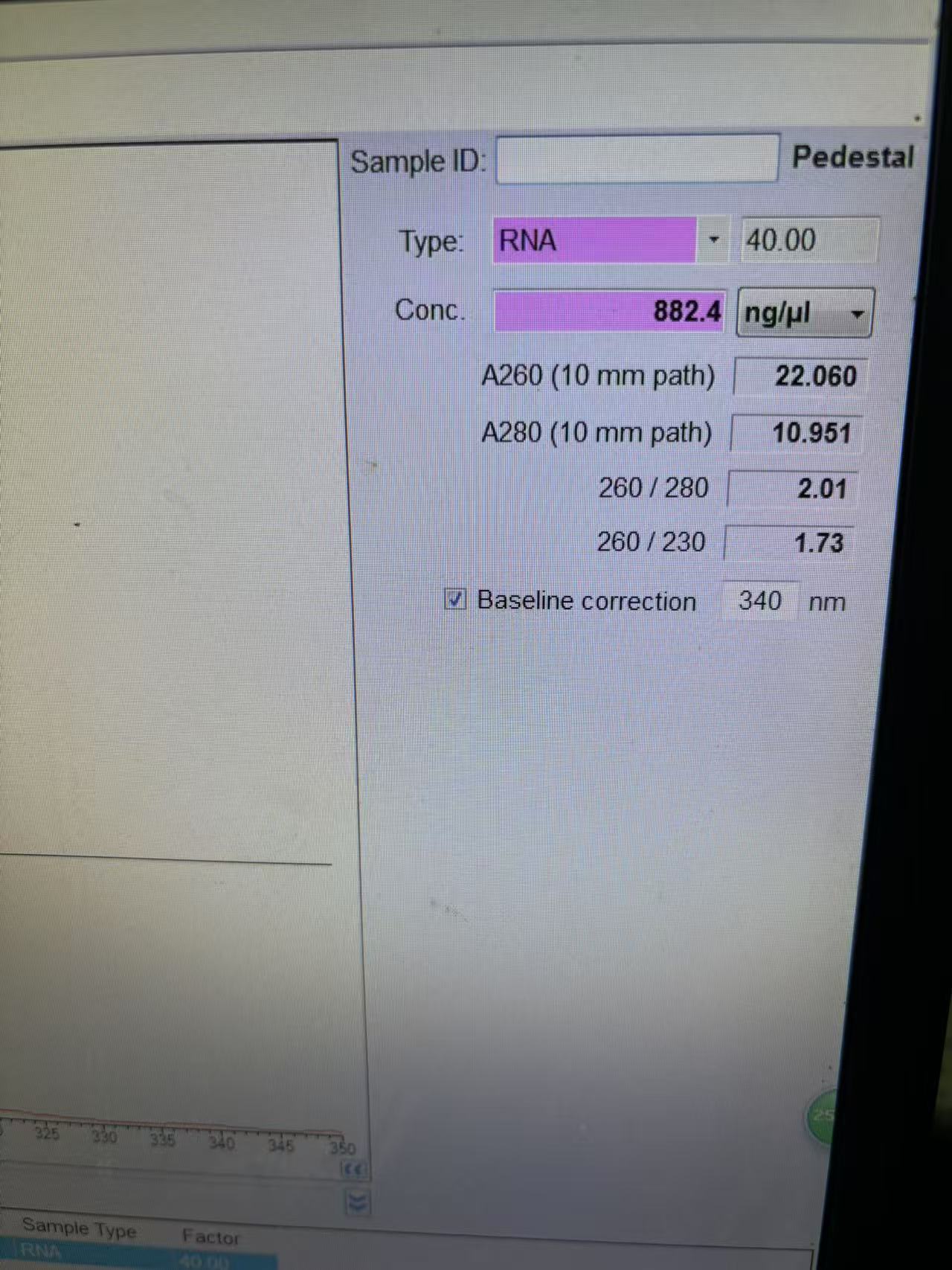
**

**EC9706-EV:**

**
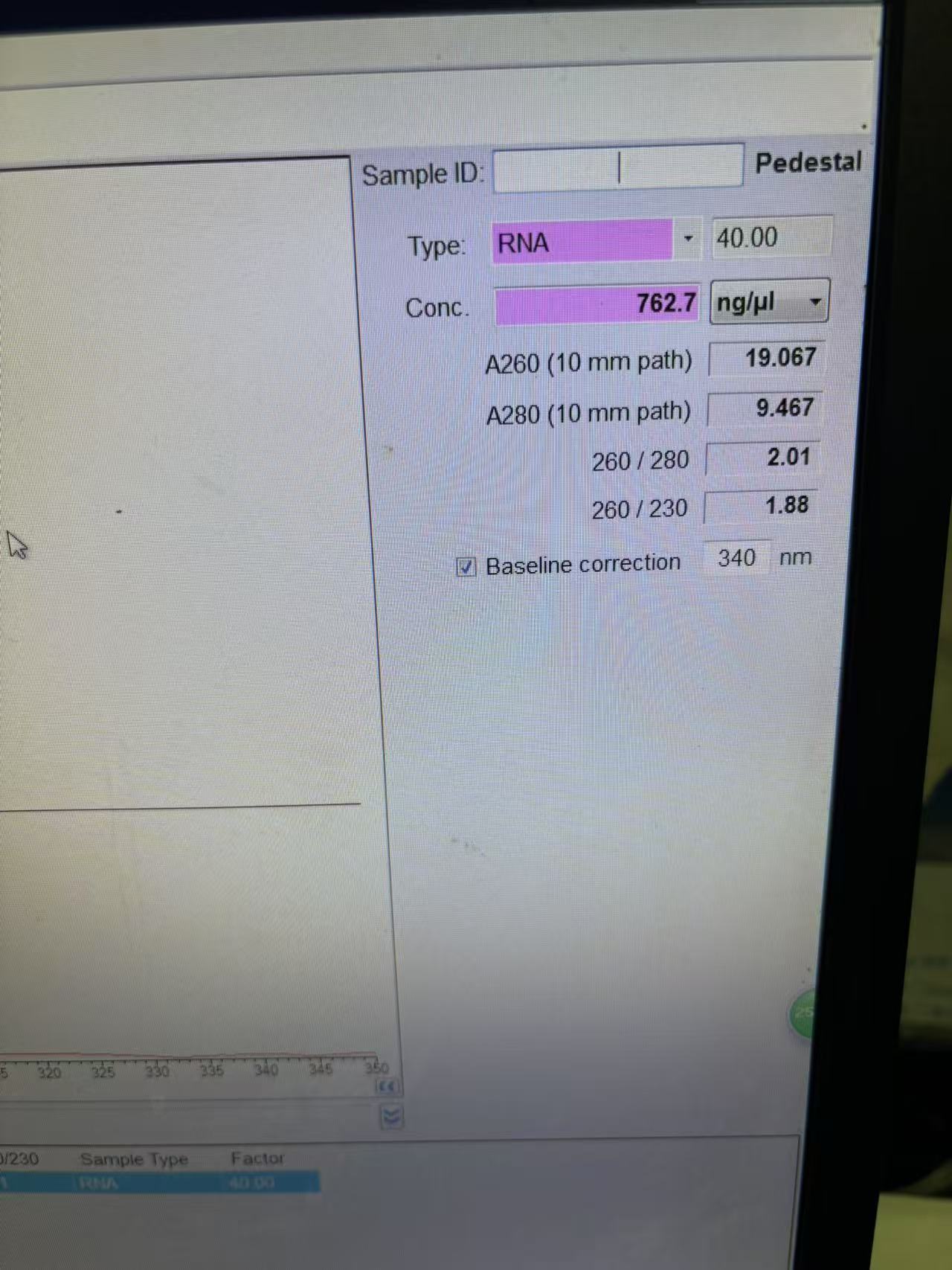
**

**EC9706-EV+miR-203a-3p:**

**
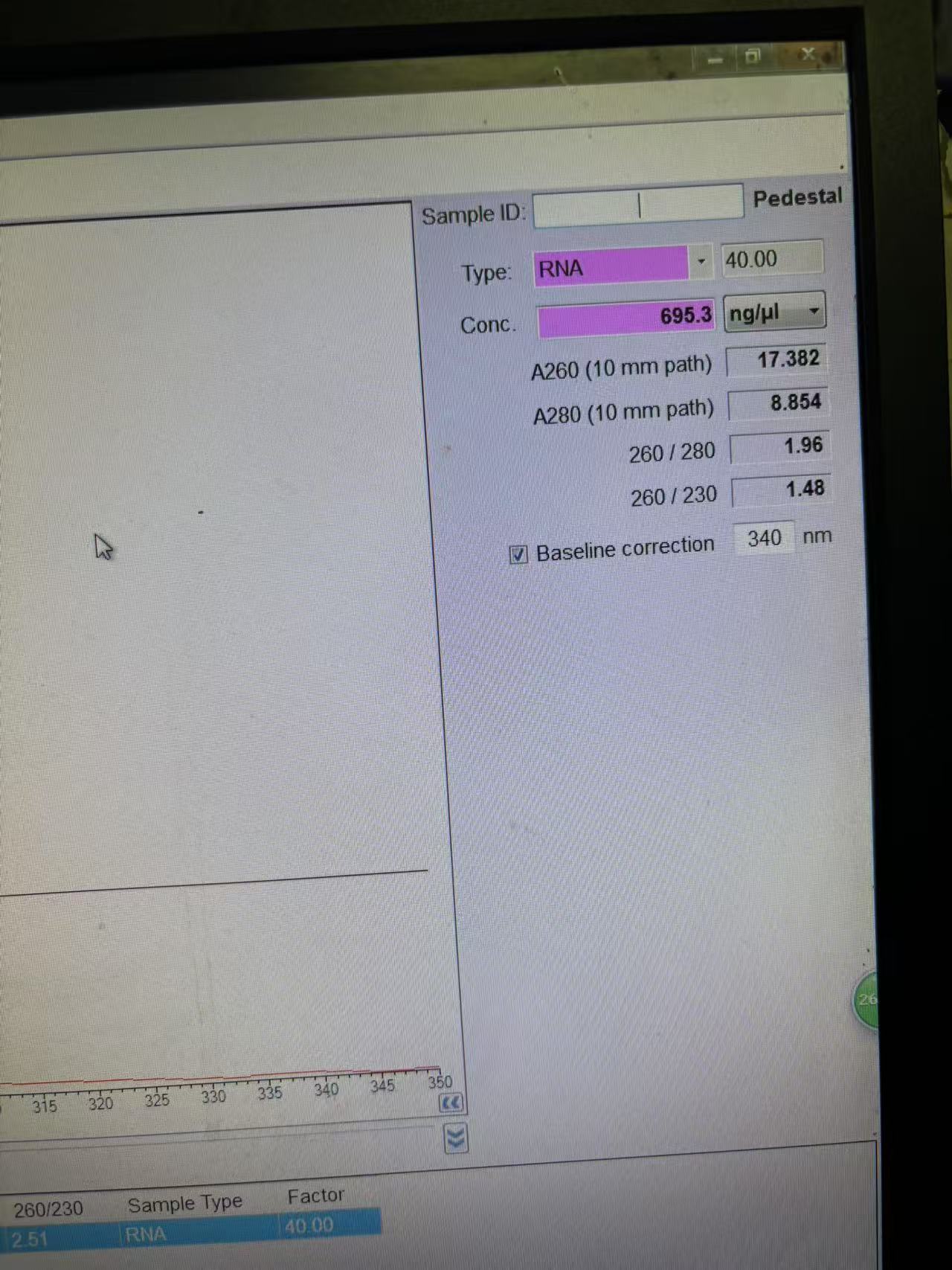
**

**EC9706-Drp1:**

**
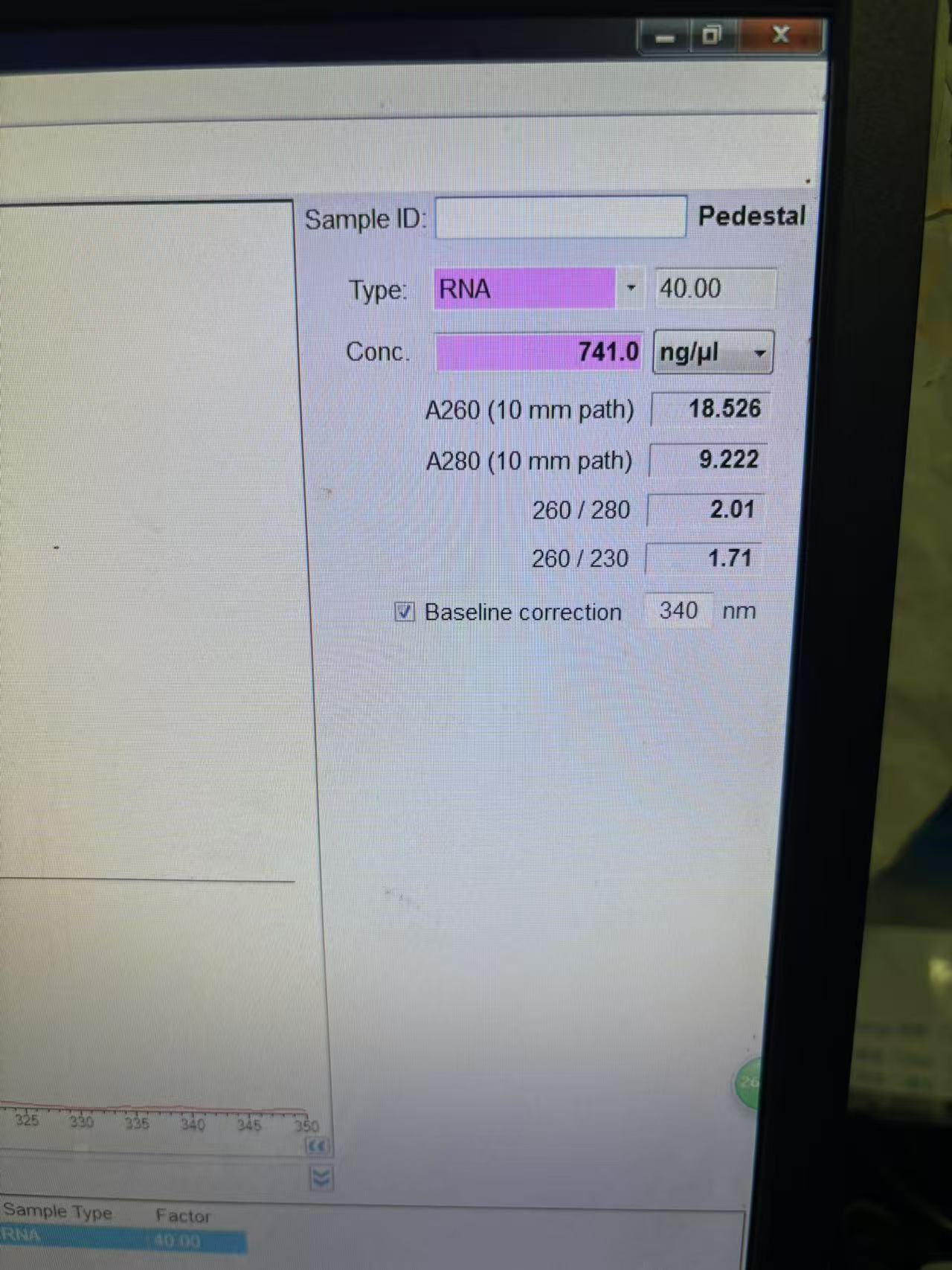
**

**EC9706-Drp1+miR-203a-3p:**

**
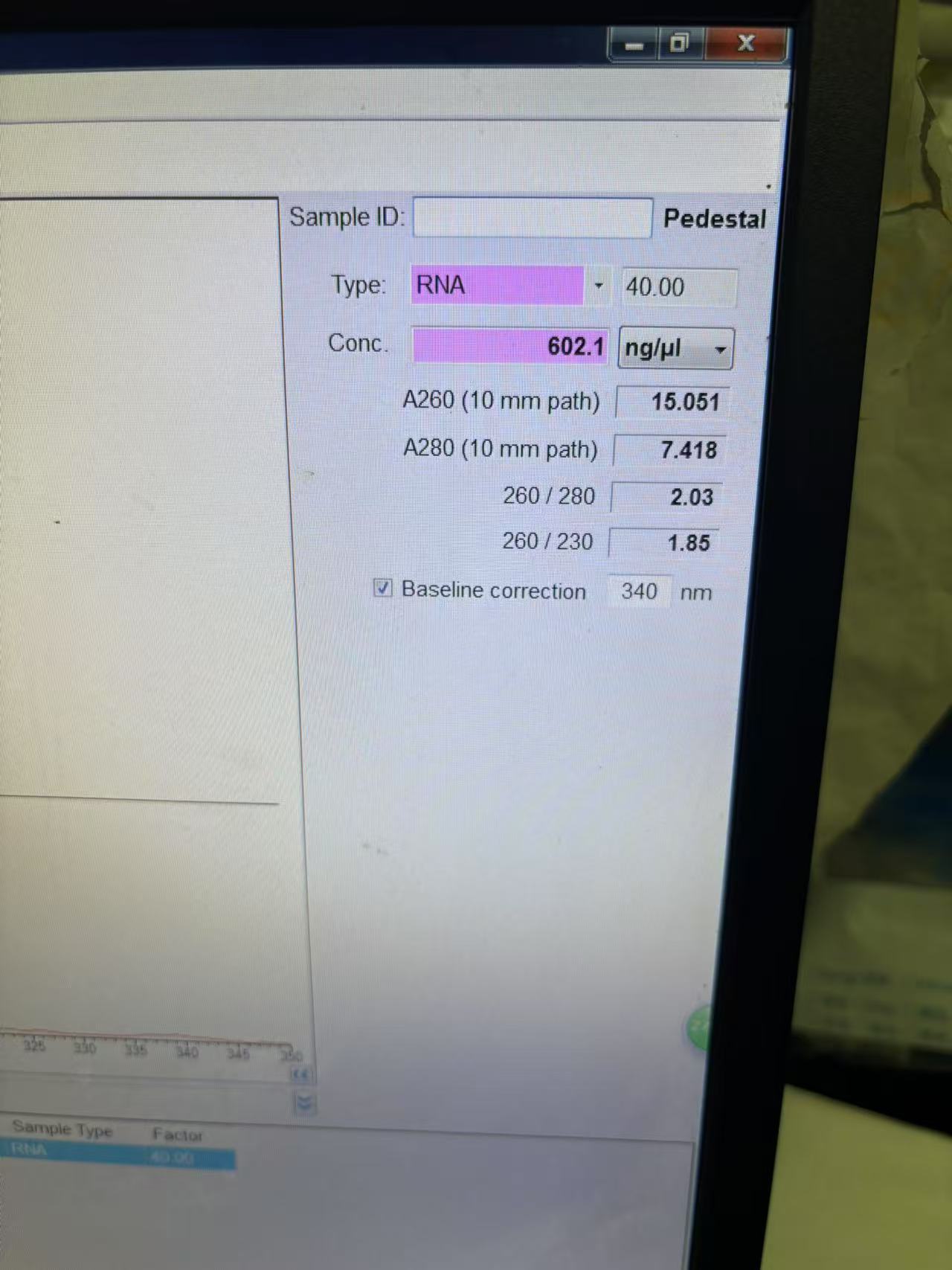
**

**the melting curves for qRT-PCR.**

**Figure 1E**


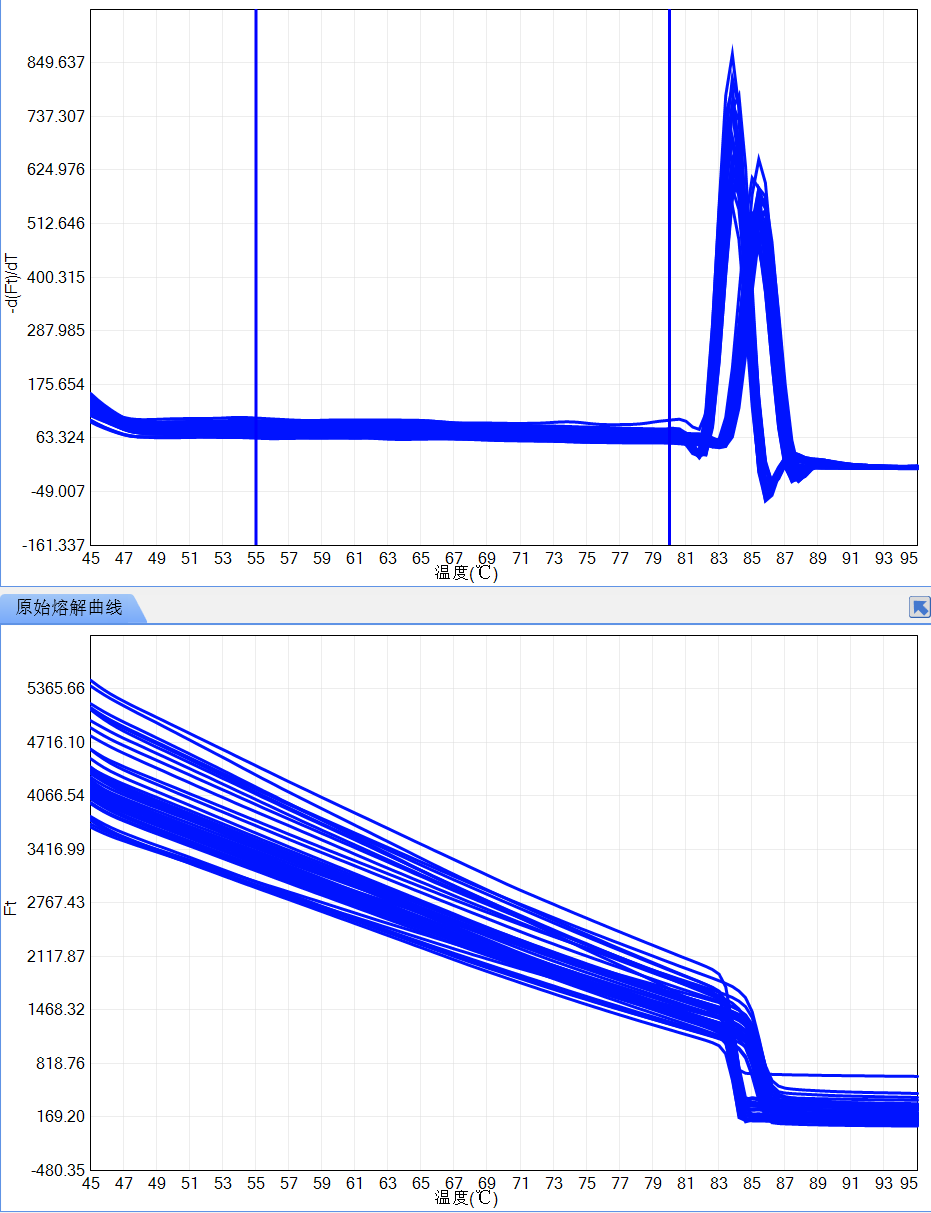


**Figure 5B**


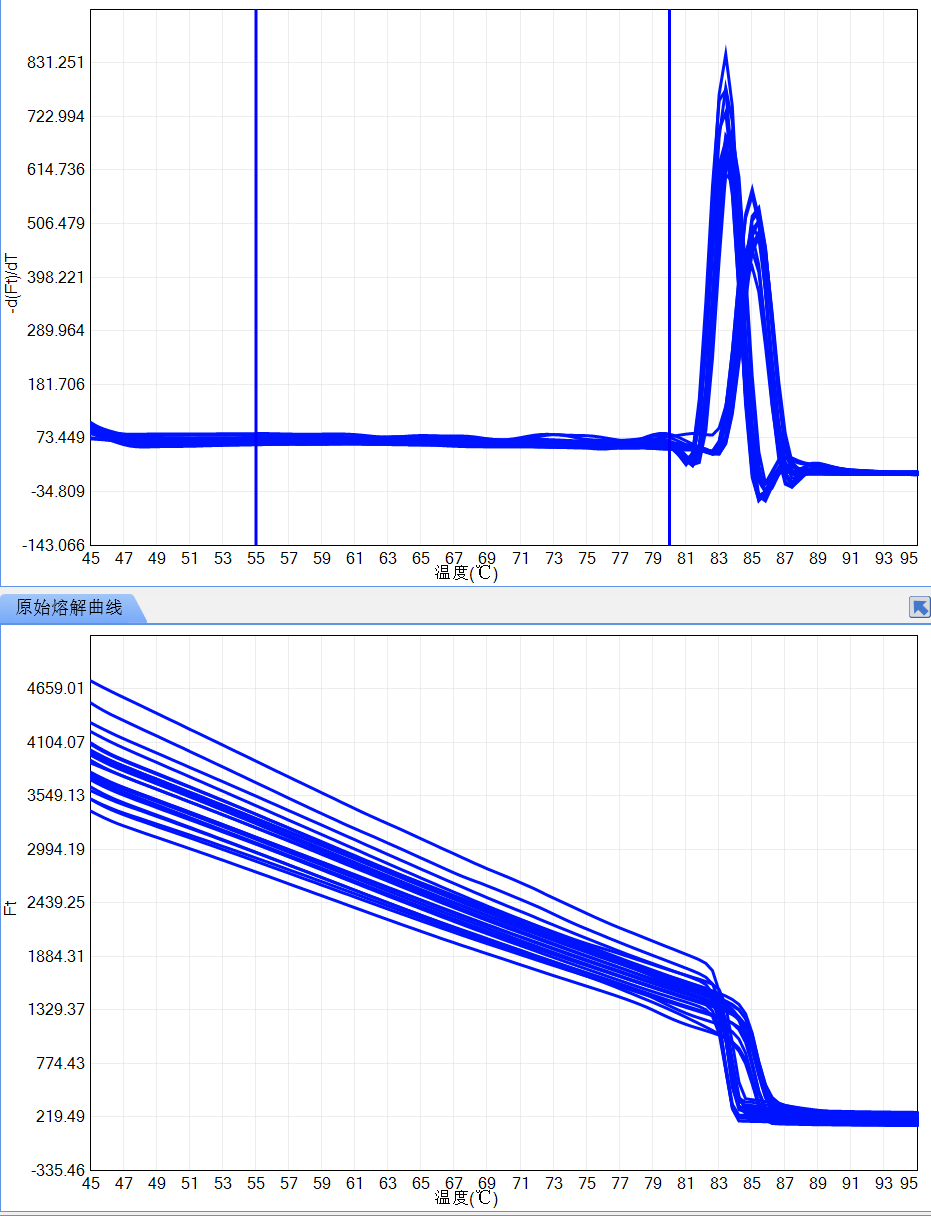


**Figure 5C**


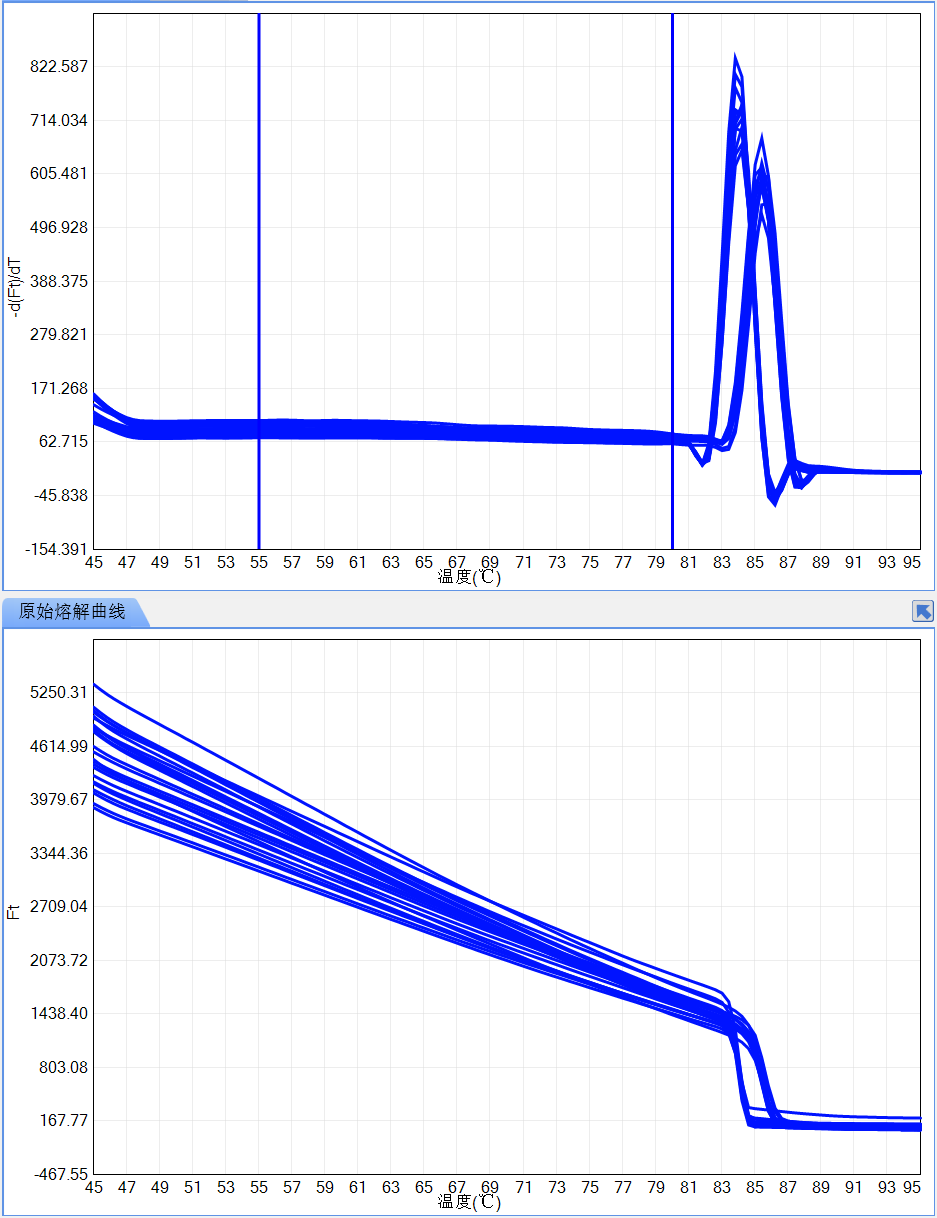

Supplement: Supplementary file 2 — Additional file 2 [file 12967_2025_6697_MOESM2_ESM.docx]
